# Heterogeneously catalyzed thioether metathesis by a supported Au–Pd alloy nanoparticle design based on Pd ensemble control†

**DOI:** 10.1039/d4sc02732a

**Published:** 2024-06-26

**Authors:** Takehiro Matsuyama, Takafumi Yatabe, Tomohiro Yabe, Kazuya Yamaguchi

**Affiliations:** a Department of Applied Chemistry, School of Engineering, The University of Tokyo 7-3-1 Hongo, Bunkyo-ku Tokyo 113-8656 Japan kyama@appchem.t.u-tokyo.ac.jp yatabe@appchem.t.u-tokyo.ac.jp +81-3-5841-7220; b Precursory Research for Embryonic Science and Technology (PRESTO), Japan Science and Technology Agency (JST) 4-1-8 Honcho, Kawaguchi Saitama 332-0012 Japan

## Abstract

C–S bond metathesis of thioethers has gained attention as a new approach to the late-stage diversification of already existing useful thioethers with molecular frameworks intact. However, direct or indirect thioether metathesis is scarcely reported, and heterogeneously catalyzed systems have not been explored. Here, we develop heterogeneously catalyzed direct thioether metathesis using a supported Au–Pd alloy nanoparticle catalyst with a high Au/Pd ratio. The Au-diluted Pd ensembles suppress the strong π-adsorption of diaryl thioethers on the nanoparticles and promote transmetalation *via* thiolate spill-over onto neighboring Au species, enabling an efficient direct thioether metathesis.

## Abstract 2

Heterogeneously catalyzed direct thioether metathesis has been developed using a supported Au–Pd alloy nanoparticle catalyst with a high Au/Pd ratio.

## Graphical abstract

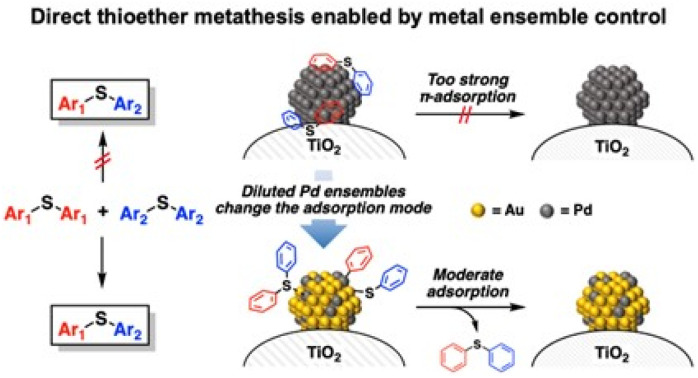

## Introduction

Multimetallic nanocatalysts can exhibit higher catalytic performance and/or selectivity than their monometallic counterparts. This high performance is usually attributed to three alloy effects—ensemble, ligand, and strain effects^1^—which are difficult to study in isolation. Nevertheless, according to some reports, the dominant effect is metal ensembles on the nanoparticle surfaces.^1*a*^ The decreased ensembles of active metal species alter the adsorption configuration of molecules^2^ or exhibit a single atom-like character,^3^ resulting in unique catalytic properties. Such catalyst designs based on ensemble control are uniquely applicable to nanoparticle catalysts and have the potential to realize novel molecular transformations.

**Scheme 1:**
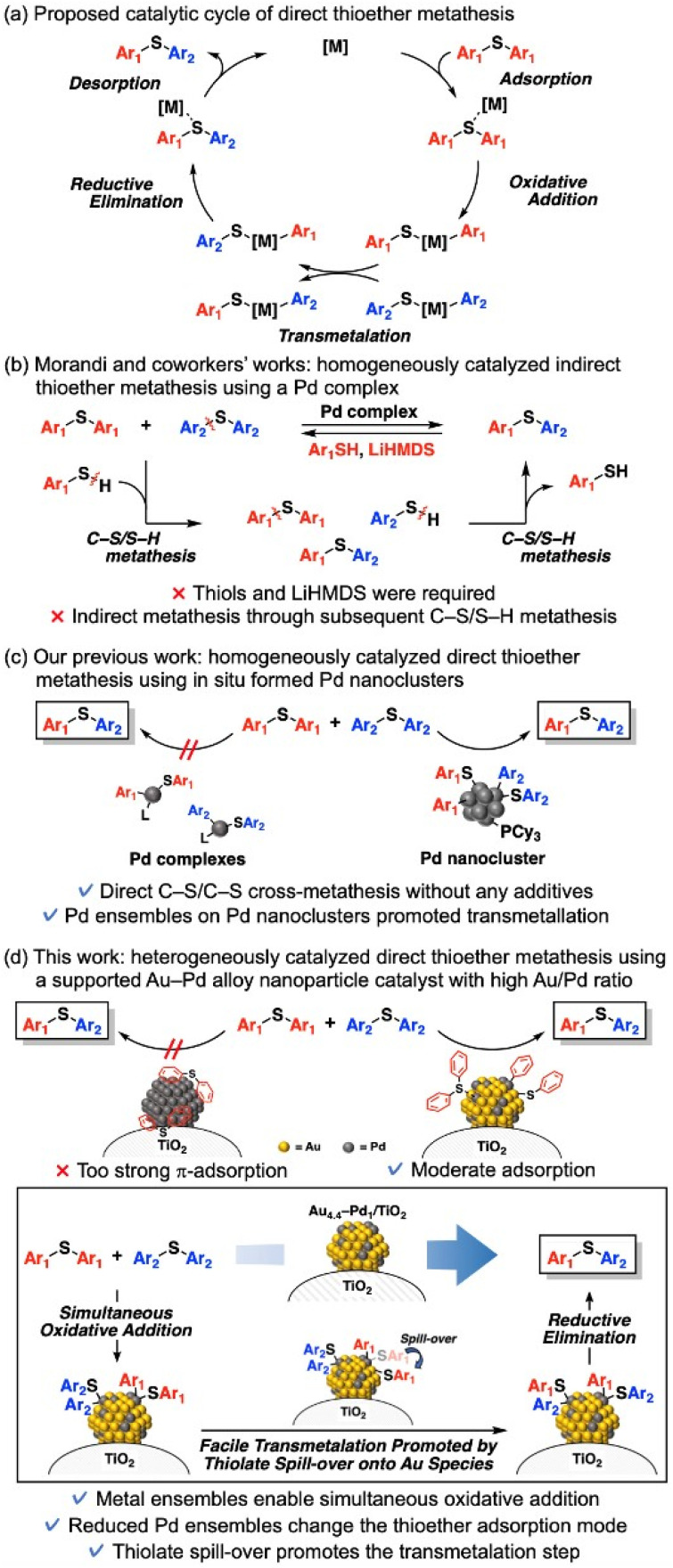
Background (a)–(c) and (d) overview of the present study.

Diaryl thioethers are widely used in polymers, natural products, bioactive compounds, and pharmaceuticals.^4*a*–*c*^ Late-stage diversification of diaryl thioethers, which enables functionalization and transformation of complex molecules without disrupting their building blocks, has therefore become an important goal in synthetic organic chemistry, medicinal science, and materials science.^4^ One desirable approach is C–S bond metathesis of diaryl thioethers, which synthesizes novel diaryl thioethers from already existing useful thioethers. The first C–S bond metathesis of diaryl thioethers was reported by Morandi *et al.*,^5*a*^ who also constructed porous organic polymers with two- and three-dimensional cores under mild reaction conditions (∼80 °C) using a homogeneous Pd complex catalyst.^5*b*^ Theoretically, direct C–S bond metathesis of diaryl thioethers can proceed through the following catalytic cycle: adsorption and oxidative addition of thioethers to Pd species, transmetalation between oxidative adducts, and then reductive elimination and desorption of thioethers from Pd species (Scheme 1a). However, the direct metathesis does not occur in their reports^5*a*,*b*^ possibly because the transmetalation step is prohibitively difficult on Pd complex catalysts. Thus, thiols and lithium bis(trimethylsilyl)amide are required for their reaction systems: indirect C–S/C–S cross-metathesis *via* C–S/S–H metathesis between diaryl thioethers and thiols (Scheme 1b).^5^ Quite recently, Audisio *et al.* reported Ni-catalyzed direct C–S/C–S cross-metathesis of various thioethers for isotope labelling without using thiols and bases;^6^ however, C–S bond metathesis of diaryl thioethers was not demonstrated. On the other hand, recently, we achieved the first example of direct C–S bond metathesis of diaryl thioethers using only Pd acetate and tricyclohexylphosphine (PCy_3_) as catalyst precursors.^7^ The active species of metathesis was confirmed as *in situ*-formed Pd nanoclusters, which likely enable direct crossover between two oxidative adducts (Scheme 1c). However, heterogeneously catalyzed C–S bond metathesis of diaryl thioethers, including indirect metathesis, is demanded for practical use and green sustainable chemistry but has not been attained. In fact, supported monometallic Pd nanoparticles hardly catalyze C–S bond metathesis between phenyl sulfide (1a) and *p*-tolyl sulfide (1b), even with PCy_3_ (Table S1†).^7^ Clearly, an additional catalyst design is required for heterogeneously catalyzed thioether metathesis.

This study proposes a heterogeneously catalyzed efficient direct C–S bond metathesis of diaryl thioethers using a TiO_2_-supported Au–Pd alloy nanoparticle catalyst (Au_4.4_–Pd_1_/TiO_2_) without any additives (Scheme 1d). This catalytic system exhibits a wide substrate scope and functional group tolerance; moreover, the catalyst can be reused a few times. Catalyst characterization and density functional theory (DFT) calculations of cluster models showed that when the Pd ensembles are diluted by Au alloying, the changed adsorption mode of thioethers on the nanoparticles lowers the adsorption/desorption energy and enables C–S bond metathesis. We also suggest that oxidative addition of thioethers produces thiolate species on the Pd species, which migrate to the Au species and promote the transmetalation step, thereby achieving efficient thioether metathesis.

## Results and discussion

**Fig. 1:**
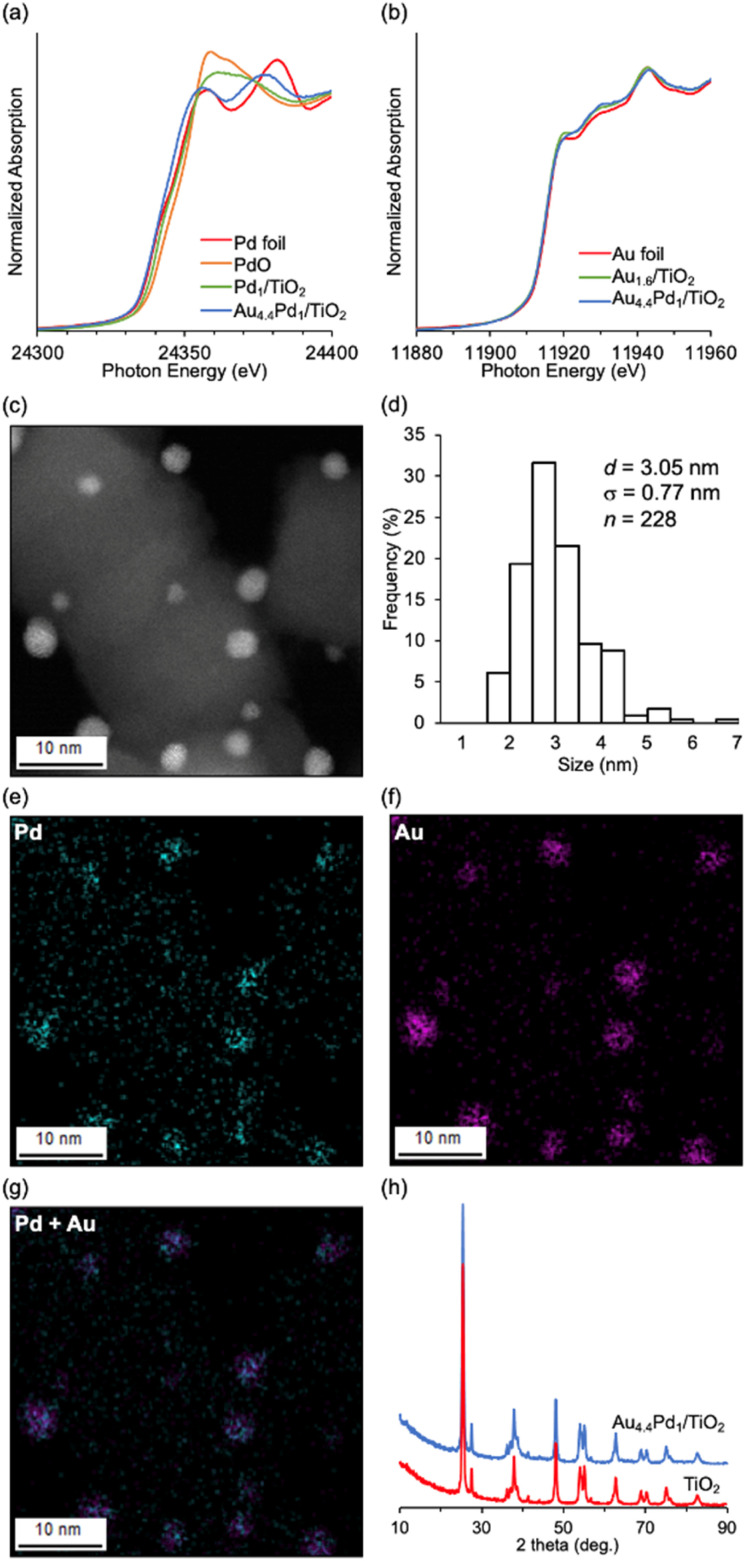
Characterization of Au_4.4_–Pd_1_/TiO_2_: (a) Pd K-edge and (b) Au L_III_-edge XANES spectra and (c) HAADF-STEM image of Au_4.4_−Pd_1_/TiO_2_; (d) size distribution of nanoparticles on Au_4.4_–Pd_1_/TiO_2_; (e)–(g) STEM-EDS mappings of Au_4.4_–Pd_1_/TiO_2_ showing the distributions of Pd [cyan, panel (e)] and Au [magenta, panel (f)]; (g) overlap of (e) and (f); (h) XRD patterns of TiO_2_ and Au_4.4_–Pd_1_/TiO_2_.

Au_4.4_–Pd_1_/TiO_2_ was prepared *via* simultaneous deposition–precipitation of Au and Pd hydroxides (Au/Pd molar ratio = 4.4) on TiO_2_, followed by reduction using NaBH_4_ in deionized water. The Pd K-edge and Au L_III_-edge X-ray absorption near edge structure (XANES) spectra of Au_4.4_–Pd_1_/TiO_2_ were similar to those of Pd foil and Au foil, respectively (Fig. 1a and b), indicating zero valence of Pd and Au, as inferred from the Pd 3d and Au 4f X-ray photoelectron spectra of Au_4.4_–Pd_1_/TiO_2_ (Fig. S1†). From high-angle annular dark-field-scanning transmission electron microscopy (HAADF-STEM) images, the mean diameter of the TiO_2_-supported metal nanoparticles was determined as 3.05 nm (*σ* = 0.77 nm; Fig. 1c and d). From the almost coincident locations of Pd and Au species in the STEM-energy-dispersive spectroscopy (EDS) mapping of Au_4.4_–Pd_1_/TiO_2_ (Fig. 1e–g) and the fitting of the Pd K-edge and Au L_III_-edge extended X-ray absorption fine structure spectra (indicating that the scatterings originated from Au–Pd bonds; see Fig. S2 and Table S2†), we inferred that Au–Pd alloy nanoparticles were supported on TiO_2_. Moreover, the X-ray diffraction (XRD) patterns of Au_4.4_–Pd_1_/TiO_2_ and TiO_2_ were comparable (Fig. 1h), confirming that the TiO_2_ structure was unchanged during the catalyst preparation.

Table 1 lists the reaction conditions for investigating the effect of Pd-based supported nanoparticle catalysts on C–S bond metathesis between 1a and 1b. A quantitative metathesis can obtain two equivalents of phenyl *p*-tolyl sulfide (1ab) on the basis of 1b. Based on our previous report,^7^ we first investigated a monometallic TiO_2_-supported Pd nanoparticle catalyst but 1ab was hardly obtained (Table 1, entry 1).‡‡Previously, we reported slight thioether metathesis between 1a and 1b in the presence of a hydroxyapatite-supported mono-metallic Pd nanoparticle catalyst^8^ but under very different reaction conditions, using much more catalyst (Pd: 20 mol%) at a higher reaction temperature (160 °C) for 24 h. Among several TiO_2_-supported Pd-based bimetallic nanoparticle catalysts, the Au–Pd alloy nanoparticle catalyst exhibited the highest catalytic performance for C–S bond metathesis (Table S3†). We therefore prepared supported Au–Pd alloy nanoparticle catalysts with various Au/Pd ratios (Au_*x*_–Pd_1_/TiO_2_, *x*: Au/Pd molar ratio). The catalytic performance of metathesis improved with increasing Au/Pd ratio in the catalyst, reaching a 1ab yield of 55% for Au_4.4_–Pd_1_/TiO_2_ (Table 1, entries 2–5). Au_1.6_/TiO_2_ hardly catalyzed the reaction and a physical mixture of Au_1.6_/TiO_2_ and Pd_1_/TiO_2_ did not improve the 1ab yield (Table 1, entries 6 and 7), indicating that Au–Pd alloy formation is essential for the present C–S bond metathesis. After optimizing the supports (Table S4†), solvents (Table S5†), reaction temperatures (Table S6†), and 1a/1b ratio (Table S7†), 1ab was efficiently produced in 77% yield (Table 1, entry 8).

**Table 1:** Effect of catalysts on the metathesis of 1a and 1b^a^

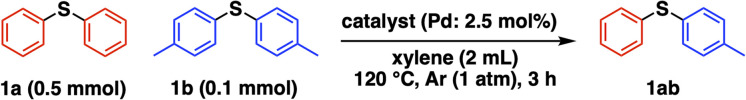				
Entry	Catalyst	Conversion (%)		Yield (%)
		1a	1b	1ab
1	Pd_1_/TiO_2_	9	11	1
2	Au_0.7_–Pd_1_/TiO_2_	9	20	11
3	Au_1.4_–Pd_1_/TiO_2_	14	36	25
4	Au_3.0_–Pd_1_/TiO_2_	19	62	52
5	Au_4.4_–Pd_1_/TiO_2_	18	66	55
6^b^	Au_1.6_/TiO_2_	10	8	<1
7^b^	Pd_1_/TiO_2_ + Au_1.6_/TiO_2_	<1	3	3
8^c^	Au_4.4_–Pd_1_/TiO_2_	21	84	77

a Conditions: 1a (0.5 mmol), 1b (0.1 mmol), catalyst (Pd: 2.5 mol%), xylene (2 mL), 120 °C, 3 h, Ar (1 atm). Conversions and yields were determined by GC.

b Au_1.6_/TiO_2_ (Au: 4.0 mol%).

c 140 °C, 24 h.

The C–S bond metathesis of 1a and 1b immediately ceased after hot filtration of Au_4.4_–Pd_1_/TiO_2_ (Fig. S3†), and inductively coupled plasma-atomic emission spectroscopy (ICP-AES) detected almost zero Pd and Au species in the filtrate (Pd: below the detection limit, Au: 0.004% of the Au used in the reaction). Therefore, the observed catalysis was truly heterogeneous. Although the catalyst could be regenerated *via* calcination in an air followed by reduction with NaBH_4_ and reused twice without significant loss of the final yield (Fig. S4†), the final 1ab yield dropped at the 4th use. The average size of Au–Pd nanoparticles in the Au_4.4_–Pd_1_/TiO_2_ after the 1st use observed by HAADF-STEM (*d* = 3.70 nm, *σ* = 1.12 nm) was a little larger than that of the fresh catalyst (*d* = 3.05 nm, *σ* = 0.77 nm) (Fig. 1c, d, and S5†), and a new peak assigned to Au(111) appeared in the XRD pattern of the catalyst after the 4th reuse (Fig. S6†), indicating that the aggregation of Au–Pd nanoparticles is one reason for the deactivation of the catalytic activity.

**Scheme 2:**
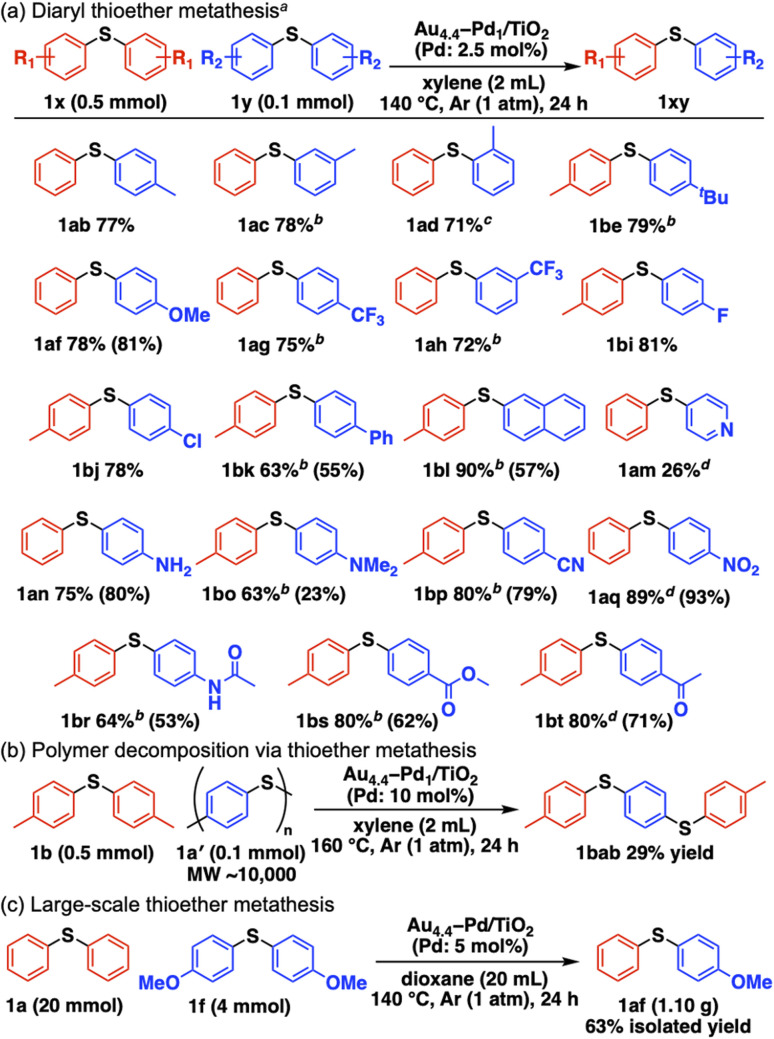
Substrate scope. ^*a*^(a) Diaryl thioether metathesis. ^*a*^Conditions: 1x (0.5 mmol), 1y (0.1 mmol), Au_4.4_–Pd_1_/TiO_2_ (Pd: 2.5 mol%), xylene (2 mL), 140 °C, 24 h, Ar (1 atm). Yields were determined by GC. Values in parentheses are isolated yields. ^*b*^Au_4.4_–Pd_1_/TiO_2_ (Pd: 5 mol%). ^*c*^Au_4.4_–Pd_1_/TiO_2_ (Pd: 10 mol%). ^*d*^Au_2.6_–Pd_1_/HAP (Pd: 10 mol%). (b) Polymer decomposition *via* thioether metathesis. Conditions are indicated in the scheme. (c) Large-scale thioether metathesis. Conditions are indicated in the scheme.

Scheme 2a summarizes the substrate scope of Au_4.4_–Pd_1_/TiO_2_-catalyzed C–S bond metathesis of diaryl thioethers.§§As for the products that are difficult to be isolated by column chromatography, GC charts after the reaction can be found in Fig. S14.† Symmetrical methyl-substituted thioethers at the *para*, *meta*, and *ortho* positions afforded the corresponding unsymmetrical thioethers in good yields (1ab–1ad). Other electron-donating groups, including 4-*tert*-butyl and 4-methoxy groups, also afforded their metathesis products (1be, 1af). This system is applicable to thioether metathesis with electron-withdrawing trifluoromethyl groups (1ag, 1ah) and halogenated thioethers with fluoro and chloro groups (1bi, 1bj). 4-Biphenyl-, 2-naphthyl-, and 4-pyridyl-substituted thioethers also served as competent metathesis partners (1bk, 1bl, 1am). Thioethers with amino (1an), *N*,*N*-dimethylamino (1bo), cyano (1bp), nitro (1aq), acetamide (1br), methyl ester (1bs), and acetyl (1bt) functional groups were converted into the corresponding unsymmetrical thioethers without disrupting the functional groups. Moreover, commercial polyphenylene sulfide (PPS, 1a′) decomposed into 1,4-bis[(4-methylphenyl)thio]benzene (1bab) *via* metathesis between 1a′ and 1b in the presence of Au_4.4_–Pd_1_/TiO_2_ (Scheme 2b). Gram-scale synthesis from 1a and 1f afforded the metathesis product 1af (1.10 g, 63% isolated yield) (Scheme 2c).

**Fig. 2:**
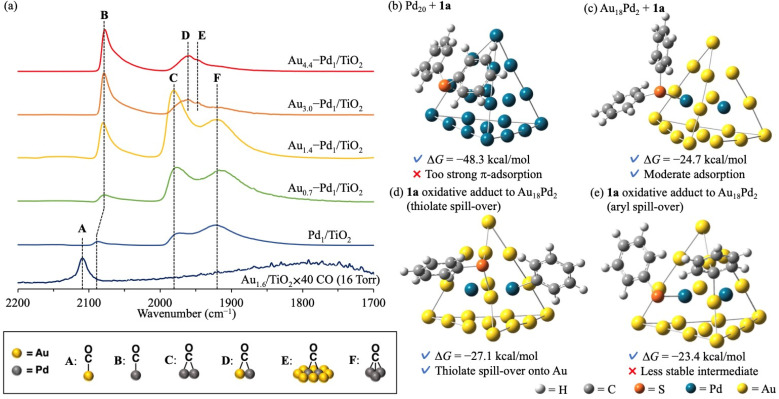
Characterization of Au–Pd alloy nanoparticle catalysts and DFT calculations of 1a adsorption using Pd_20_ and Au_18_Pd_2_ cluster models: (a) CO-DRIFT spectra of Au_1.6_/TiO_2_, Pd_1_/TiO_2_, and Au_*x*_–Pd_1_/TiO_2_; optimized structures of 1a adsorbed on (b) Pd_20_ and (c) Au_18_Pd_2_; optimized structures of 1a oxidative adduct on (d) Au_18_Pd_2_ (thiolate spill-over to Au) and (e) Au_18_Pd_2_ (aryl spill-over to Au).

The effect of alloying on the present C–S bond metathesis of thioethers was determined from diffuse reflectance infrared Fourier transform (CO-DRIFT) spectra of adsorbed CO on Au_1.6_/TiO_2_, Pd_1_/TiO_2_, and Au_*x*_–Pd_1_/TiO_2_ (Fig. 2a). In the CO-DRIFT spectra of Pd_1_/TiO_2_, Au_0.7_–Pd_1_/TiO_2_, and Au_1.4_–Pd_1_/TiO_2_, which exhibited low catalytic activity for the C–S bond metathesis (Table 1, entries 1–3), three peaks around 2070, 1980, and 1920 cm^−1^ were observed assignable to linear (B), bridged (C), and three-fold (F) CO species on zero-valent Pd species, respectively.^9^ In the CO-DRIFT spectra of Au_3.0_–Pd_1_/TiO_2_ and Au_4.4_–Pd_1_/TiO_2_ showing high catalytic activity (Table 1, entries 4 and 5), peaks C and F were absent while new peaks at 1950 and 1940 cm^−1^ were attributable to CO species bridged on Au and Pd species (D) and on Pd species surrounded by Au species (E), respectively.^9*d*^ As the Pd and Au–Pd alloy nanoparticles were similarly sized (Fig. 1c, d and S7†) and the peak of the CO species on Pd did not shift with increasing Au/Pd ratio in Au_*x*_–Pd_1_/TiO_2_ (Fig. 2a), we attributed the high catalytic activity of Au_4.4_–Pd_1_/TiO_2_ to the diluted Pd ensembles rather than to the ligand effect.

Next, the ensemble effect on the present C–S bond metathesis was investigated through DFT calculations on Pd_20_ and Au_18_Pd_2_ cluster models. Referring to previous reports, we adopted Gaussian 16 (M06 functional with SDD basis sets for Au and Pd and 6-31G(d,p) basis sets for H, C, and S)^10^ (see ESI for the calculation methods and model selection) (Fig. S8–S10).† The adsorption Gibbs energy of 1a was calculated at the center site of the Pd_20_ or Au_18_Pd_2_ cluster model.¶¶The other adsorption sites are discussed in detail in the ESI.† The adsorption Gibbs energy of 1a on Pd_20_ is very high (Δ*G* = −48.3 kcal mol^−1^), indicating that the metathesis product cannot easily desorb and the reaction is strongly inhibited (Fig. 2b). In contrast, 1a adsorbed on Au_18_Pd_2_ has a moderate Δ*G*(−24.7 kcal mol^−1^) and the reaction can proceed (Fig. 2c). The optimized structure of 1a on Pd_20_ clarifies strong π-adsorption between 1a and the Pd_20_ facets, whereas 1a on Au_18_Pd_2_ adheres *via* coordination of its S atom to the Pd center with almost no π-adsorption. Corroborating this finding, the natural-bond orbital charge of the phenyl group is higher in Pd_20_-adsorbed 1a than in free 1a, probably due to π-back donation from the Pd species to the phenyl rings of 1a,^11^ but is lower in Au_18_Pd_2_–adsorbed 1a than in free 1a, possibly due to σ-donation from the S atom to the Pd atom (Fig. S11†). Facilitated by the weak π-back donation ability of the Au species,^12^ the Pd ensembles diluted with Au alloy changed the 1a adsorption mode and lowered the adsorption energy to enable the reaction (Scheme 1d).||||Previously we identified *in situ* formed Pd nanoclusters as the active species of diaryl thioether metathesis.^7^ In that case, π-adsorption of diaryl thioethers on Pd nanoclusters was probably suppressed by moderate steric hindrance of the PCy_3_ ligand, enabling the metathesis to proceed. Moreover, as indicated in the optimized structure of 1a oxidative adducts to Au_18_Pd_2_ (Fig. S12 and S13†), the thiolate species produced by thioether oxidative addition on Pd species can transfer to the Au species without forming very stable structures (Fig. 2d), whereas the 1a oxidative adduct *via* aryl spill-over to Au species gives a slightly higher Δ*G* (Fig. 2e).¶ Considering the aforementioned indirect thioether metathesis on Pd complexes,^5^ thiolate spill-over onto the Au species probably promotes the transmetalation, enabling an efficient direct diaryl thioether metathesis with no additives (Scheme 1d).

## Conclusions

In conclusion, we achieved the first heterogeneously catalyzed direct C–S bond metathesis of diaryl thioethers with a wide substrate scope and functional group tolerance using Au_4.4_–Pd_1_/TiO_2_. Catalyst characterization and DFT calculations revealed the likely causes of the high catalytic activity of Au_4.4_–Pd_1_/TiO_2_: moderate adsorption energy of thioethers on the Au–Pd alloy nanoparticles (conferred by diluted Pd ensembles) and thiolate spillover onto Au species (which promotes subsequent transmetalation). These findings are anticipated to guide the development of novel molecular transformations using multimetallic catalysts, especially by harnessing metal ensembles.

## Data availability

The data (experimental procedures and characterization data) that support this article is available within the article and its ESI.†

## Author contributions

T. Yatabe and K. Y. conceived and supervised the project. T. M. performed most of the experiments. T. Yabe performed the XAFS measurements. All authors contributed to data analysis and discussed the results. T. M. and T. Yatabe wrote the manuscript with feedback from K. Y. and T. Yabe.

## Conflicts of interest

There are no conflicts to declare.

## Supplementary Material

SC-015-D4SC02732A-s001
