# Supplementary material for: Heterogeneously catalyzed thioether metathesis by a supported Au–Pd alloy nanoparticle design based on Pd ensemble control
Source: Chem Sci. 2024 Jun 26;15(30):11884–9. doi: 10.1039/d4sc02732a (PMC11290439; doi:10.1039/d4sc02732a)
Supplement: SC-015-D4SC02732A-s001 [file SC-015-D4SC02732A-s001.pdf]

Electronic Supplementary Material (ESI) for Chemical Science.
This journal is © The Royal Society of Chemistry 2024

Electronic Supplementary Information (ESI) for

Heterogeneously catalyzed thioether metathesis by a supported Au–Pd alloy nanoparticle design based on Pd ensemble control

Takehiro Matsuyama,[a] Takafumi Yatabe,*[ab] Tomohiro Yabe,[a] and Kazuya Yamaguchi *[a]

[a]Department of Applied Chemistry, School of Engineering, The University of Tokyo, 7-3-1 Hongo, Bunkyo-ku, Tokyo 113-8656, Japan.

[b]Precursory Research for Embryonic Science and Technology (PRESTO), Japan Science and Technology Agency (JST), 4-1-8 Honcho, Kawaguchi, Saitama 332-0012, Japan.

*e-mail: yatabe@appchem.t.u-tokyo.ac.jp, kyama@appchem.t.u-tokyo.ac.jp

Table of Contents

Methods and Spectral Data of Synthesized Substrates **S2–S12**

Spectral Data of Products **S13–S20**

Supplementary References **S21**

Supplementary Figures **S22–S31**

Supplementary Tables **S32–S35**

NMR Spectra **S36–S46**

Cartesian Coordinates of Optimized Structures **S47–S72**

Methods and Spectral Data of Synthesized Substrates

Instrumental and Reagents

Gas chromatography (GC) analyses were conducted on Shimadzu GC-2014 equipped with a flame ionization detector (FID) and an InertCap-5 (60 m) using Shimadzu C-R8A Chromatopac Data Processor for area calculations. GC mass spectrometry (GC-MS) spectra were performed by Shimadzu GCMS-QP2020 equipped with an InertCap-5 MS/NP capillary column (30 m) at an ionization voltage of 70 eV. Liquid-state NMR spectra were recorded on JEOL JNM-ECA-500. ^{1}H and ^{13}C NMR spectra were measured at 500.16 and 125.77 Hz respectively. ^{1}H and ^{13}C NMR chemical shifts were referenced to tetramethylsilane (TMS) signal ($\delta = 0$ ppm) or the solvent peak (^{13}C NMR using dimethyl sulfoxide-d_6: $\delta = 39.5$ ppm). ^{19}F NMR spectra were measured at 470.62 Hz using CF_3COOH as an external reference ($\delta = -77.0$ ppm). ICP-AES analyses were conducted by Shimadzu ICPS-8100. Scanning transmission electron microscopy (STEM) observations were performed on a JEOL JEM-ARM200F. XRD patterns were recorded on a Rigaku SmartLab diffractometer (Cu Kα, $\lambda = 1.5405$ Å, 45 kV, 20 mA). Au loadings of supported Au–Pd alloy nanoparticle catalysts were determined by X-ray fluorescence spectrometry (XRF) measurement using ZSX PrimusII (Rigaku, Japan). X-ray absorption spectroscopy (XAS) was carried out at the BL14B2 beamline of SPring-8 (Proposal No. 2023A1512). The XPS spectra were measured on an Ulvac-Phi PHI5000 VersaProbe at the Advanced Characterization Nanotechnology Platform of the University of Tokyo. The binding energies were calibrated by using the C 1s signal at 284.8 eV. Diffuse reflectance infrared Fourier transform spectroscopy (DRIFT) was performed using FT/IR-6700spectrometer (JASCO). Supported nanoparticle catalysts were exposed to 16 Torr of CO followed by evacuation for 1 min, and then, the DRIFT measurement of CO adsorbed on the catalysts was performed. Only in the case of $Au_{1.6}/TiO_2$, the DRIFT of adsorbed CO was measured under 16 Torr of CO. The DRIFT spectra measured in vacuo were subtracted as the backgrounds. Solvents and substrates were obtained from Tokyo Chemical Industry, Aldrich, Kanto Chemical, or FUJIFILM Wako Pure Chemical (reagent grade), and purified prior to being used, if necessary.

XAS Measurements and EXAFS Fitting

X-ray absorption spectroscopy (XAS) was carried out at the BL14B2 beamline of SPring-8 (Proposal No. 2023A1512). Pd K-edge and Au L_{III}-edge X-ray absorption fine structure (XAFS) measurements were conducted in transmission and fluorescence mode using a Si (311) crystal monochromator. Each sample was formed into a pellet, doubly wrapped in a plastic laminate pack, and heat-sealed in an air atmosphere. X-ray absorption near-edge structure (XANES) and extended X-ray absorption fine structure (EXAFS) data were analyzed using Athena and Artemis software (Demeter, ver. 0.9.025; Bruce Ravel). The data reduction procedure for EXAFS consisted briefly of the following steps: pre-edge subtraction, background determination, normalization, and spectra

averaging. The edge position is defined to be the first inflection point on the leading absorption peak. The energy was calibrated using Pd foil for the Pd K-edge XAS and Au foil for the Au L_{III}-edge XAS. The background in the EXAFS region was approximated using a cubic spline routine and optimized according to the criteria described by Cook and Sayers.[S1] Then, the spectra were normalized by the edge-step at 50 eV after the absorption edge. The k^3-weighted EXAFS functions were Fourier transformed, filtered, and fitted in R-space in the range of 3–13 Å^{-1} for Au and 3–12 Å^{-1} for Pd. Fourier filtering was used to isolate the contributions of specific shells and to eliminate low frequency background and high frequency noise. Fourier filtering was done by choosing a window in the Fourier transform spectrum and calculating the inverse Fourier Transform of the selected R range. The interatomic distance (R), the first nearest-neighbor coordination number (C.N.), the difference of the Debye–Waller factor from the reference (σ^2), and the correction of the threshold energy (ΔE_{j0}) were treated as free parameters during the fitting. The quality of the fit was estimated from R-factor. R-factor represented the residuals between the observed and calculated spectrum in the fitted range. Low values of R-factor indicated a good agreement between the data and model. Fitting analysis using k^3-weighted Fourier transforms was applied to obtain a unique set of C.N. and σ^2 parameters. Those parameters are highly correlated, and there are several different combinations of C.N. and σ^2 that can lead to similar fits; however, the set of combinations depends on the k^3-weight factor.[S2] Therefore, a unique set of parameters might be found by fitting on k^3-weighted Fourier transforms. To analyze the spectra, simulations of reference compounds using FEFF6 were used to calculate phase shifts and backscattering amplitude. FEFF references were obtained for Pd–Pd by utilizing crystallographic data of Pd (crystal system: cubic),[S3] for Pd–O by utilizing crystallographic data of PdO (crystal system: tetragonal),[S4] for Au–Au by utilizing crystallographic data of Au (crystal system: cubic),[S5] for Pd–Au by utilizing a model in which one of the Au or Pd atoms was substituted for Pd or Au in the crystal data model of Au or Pd.

Preparation of Supported Catalysts

$Au_{4.4}$–Pd_1/TiO_2 was prepared as follows: The aqueous solution (60 mL) of $HAuCl_4{\cdot}4H_2O$ (247.1 mg, 10.0 mM), $PdCl_2$ (17.7 mg, 1.7 mM), and KCl (29.8 mg, 6.7 mM) containing TiO_2 (2.0 g) was vigorously stirred at room temperature. After 15 min, the pH of the solution was adjusted to 9.0 by addition of an aqueous solution of NaOH (1.0 M), and the resulting slurry was stirred overnight. The solid was then filtered off, washed with a large amount of water (2 L), and dried to afford 2.0 g of $Au_{4.4}(OH)_x$–$Pd_1(OH)_x/TiO_2$ as an ocher powder. $Au_{4.4}(OH)_x$–$Pd_1(OH)_x/TiO_2$ (2.0 g) was added to water (60 mL), and then added $NaBH_4$ (240 mg) in an open air. The mixture was vigorously stirred at room temperature for 2 h. The solid was then filtered off, washed with deionized water (2 L), and dried under an Ar atmosphere to afford 2.0 g of $Au_{4.4}$–Pd_1/TiO_2 as black powder (Au content: 3.85 wt%, Pd content: 0.47 wt%). Various supported Au–Pd alloy nanoparticle catalysts, such as $Au_{5.4}$–

Pd_1/LDH (Au content: 5.4 wt%, Pd content: 0.54 wt%), $Au_{1.4}$–Pd_1/CeO_2 (Au content: 1.2 wt%, Pd content: 0.45 wt%), $Au_{2.5}$–Pd_1/ZrO_2 (Au content: 1.9 wt%, Pd content: 0.42 wt%), $Au_{3.6}$–Pd_1/Al_2O_3 (Au content: 3.2 wt%, Pd content: 0.47 wt%), and $Au_{2.6}$–Pd_1/HAP (Au content: 2.3 wt%, Pd content: 0.48 wt%) were prepared in the same method by changing the supports. Also, TiO_2-supported Au–Pd alloy nanoparticle catalysts with various Au/Pd ratios, such as $Au_{3.0}$–Pd_1/TiO_2 (Au content: 1.43 wt%, Pd content: 0.48 wt%), $Au_{1.4}$–Pd_1/TiO_2 (Au content: 0.65 wt%, Pd content: 0.46 wt%), $Au_{0.7}$–Pd_1/TiO_2 (Au content: 0.33 wt%, Pd content: 0.50 wt%), $Au_{1.6}/TiO_2$ (Au content: 0.84 wt%), and Pd_1/TiO_2 (Pd content: 0.45 wt%) were prepared in the same method by changing the amount of metal sources. The supported amounts of Pd and Au were determined by ICP-AES. As for TiO_2-supported Au–Pd alloy nanoparticle catalysts, the Au loadings were determined by XRF. Other metal catalysts, such as Ru_6–Pd_1/TiO_2, Co_6–Pd_1/TiO_2, Ni_6–Pd_1/TiO_2, and Ag_6–Pd_1/TiO_2 were prepared in the same method using $RuCl_3\cdot nH_2O$, $CoCl_2\cdot 6H_2O$, $NiCl_2\cdot 6H_2O$, and $AgNO_3$ as a metal source, respectively. As for Co_6–Pd_1/TiO_2 and Ni_6–Pd_1/TiO_2, the catalysts were reduced and stored in an Ar-filled glovebox.

A Typical Procedure for Thioether Metathesis

In an Ar-filled glovebox, $Au_{4.4}$–Pd_1/TiO_2 (Pd: 2.5 mol%), phenyl sulfide (**1a**, 0.5 mmol), *p*-tolyl sulfide (**1b**, 0.1 mmol), 1,3,5-trimethoxybenzene or dodecane (0.1 mmol, internal standard), xylene (2 mL), and a Teflon-coated magnetic stir bar were placed in a Pyrex glass reactor (volume: ~20 mL). The mixture was stirred at 120 °C for 3 h, then the mixture was cooled down to room temperature. When naphthalene was used as the internal standard, it was added to the reaction mixture after the reaction. Conversions and product yields were determined by GC analysis using 1,3,5-trimethoxybenzene, dodecane, or naphthalene as an internal standard. As for isolation of the products, an internal standard was not added. After the reaction, the catalyst was removed by simple filtration and the filtrate was concentrated by evaporation, and then subjected to silica-gel column chromatography, giving the pure product. The products were identified by GC-MS, and NMR (^{1}H, ^{13}C, and ^{19}F).

Leaching Test

A magnetic stir bar, 1,3,5-trimethoxybenzene, **1a** (0.5 mmol), **1b** (0.1 mmol), $Au_{4.4}$–Pd_1/TiO_2 (Pd: 2.5 mol%), and xylene (2 mL) were added to a Pyrex glass reactor (volume: ~20 mL), and the reaction was carried out at 140 °C in an Ar-filled glovebox. 30 min after the start of the reaction, the reaction solution was filtered by hot filtration using a disposable syringe with a PTFE membrane filter into another Pyrex glass reactor that had been previously heated to 140 °C with a magnetic stir bar, and then the reaction solution was stirred at 140 °C for additional 150 min.

As for investigation of the leaching metal amounts, the reaction was prepared as described above, and the solution 4 h after the start of the reaction was filtered using a disposable syringe with

a PTFE membrane filter. The solvent toluene was removed using a rotary evaporator before aqua regia (1 mL) was added, and the amount of Au and Pd species leached into the reaction solution was measured by ICP-AES. The measurement wavelengths were 324.270 nm (Pd) and 242.795 nm (Au).

The results showed that the reaction was immediately stopped by hot filtration, and Pd and Au species in the solution after the reaction were hardly observed by ICP-AES (Pd: below the detection limit, Au: 0.004% of the Au species used for the reaction), confirming that $Au_{4.4}$–Pd_1/TiO_2 functioned as a heterogeneous catalyst.

Reuse Experiment

A magnetic stir bar, 1,3,5-trimethoxybenzene, **1a** (0.5 mmol), **1b** (0.1 mmol), $Au_{4.4}$–Pd_1/TiO_2 (Pd: 5 mol%), and 1,4-dioxane (2 mL) were added to a Pyrex glass reactor (volume: ~20 mL), and the reaction was carried out at 140 °C for 3 h. After the decarbonylation reaction using $Au_{4.4}$–Pd_1/TiO_2, the catalyst was retrieved by filtration in an open air through washing with 100 mL of Et_2O followed by drying *in vacuo*. Then, the catalyst was calcinated at 300 °C for 3 h in an air, reduced by $NaBH_4$ in water, and then, subjected to the metathesis between **1a** and **1b** under the same reaction conditions.

Density Functional Theory (DFT) Calculation

In this work, we used Pd_{20}, Au_4Pd_{16}, and Au_2Pd_{18} models to calculate the adsorption structures and oxidative adducts of phenyl sulfide (**1a**) based on previous reports.[S6] The DFT calculations with the M06 functional were performed using Gaussian16 Rev. C as the calculation software, the Stuttgart/Dresden basis set (SDD) with the effective core potential for Au and Pd and 6-31G(d,p) as the basis sets for the other elements, including H, C, and S atoms, based on previous reports.[S6,S7] The DFT calculations using Pd_{20}, $Au_{16}Pd_4$, and $Au_{18}Pd_2$ models were performed with spin multiplicity of 1 and charge of 0. We confirmed that the optimized structures contained no imaginary frequency. All thermodynamic data were calculated at the standard state (25 °C and 1 atm). Each structure was depicted by software Avogadro and GaussView. The Cartesian coordinates of the optimized structures are given in the end of this SI. The optimized structure of **1a**, Pd_{20}, $Au_{16}Pd_4$, and $Au_{18}Pd_2$ are shown in Fig. S8.

DFT Calculations for 1a adsorbed on Pd_{20}

Considering the symmetry of Pd_{20} cluster, *S*-atom of **1a** can adsorb on three kinds of sites on Pd_{20}: center, side, and top Pd atom of Pd_{20} (Fig. S9a). We conducted the DFT calculations of the structures of **1a** adsorbed on center, side, and top Pd atom of Pd_{20}, named Pd_{20}_**1a**_C, Pd_{20}_**1a**_S, and Pd_{20}_**1a**_T structure, respectively. The optimized structures of Pd_{20}_**1a**_C, Pd_{20}_**1a**_S, and Pd_{20}_**1a**_T and the corresponding adsorption Gibbs energies and enthalpies ($\Delta G = G_{cluster\ interacted\ with\ \mathbf{1a}} - G_{cluster} -$

G_{1a}) ($\Delta H = H_{cluster\ interacted\ with\ \mathbf{1a}} - H_{cluster} - H_{1a}$) are summarized in Fig. S9b–d. Although Pd_{20}_**1a**_C and Pd_{20}_**1a**_T are almost equally stable in the viewpoint of both adsorption Gibbs energies and enthalpies among the three structures, we used Pd_{20}_**1a**_C structure for further investigation because the majority of Pd species involved in the reaction is considered to be located at the facet of Pd nanoparticles based on the fact that a mean diameter of Pd nanoparticles in Pd_1/TiO_2 is 2.97 nm (Fig. S7a).

DFT Calculations for 1a adsorbed on Au–Pd alloy nanoclusters

Based on the aforementioned results of **1a** adsorption on Pd_{20}, experimental results that TiO_2-supported Au–Pd alloy nanoparticle catalyst with high Au/Pd ratio exhibited higher catalytic performance (Table 1), and CO-DRIFT spectra of the Au–Pd alloy catalysts (Fig. 2a), DFT calculations of **1a** adsorption on the Pd atom of $Au_{16}Pd_4$ and $Au_{18}Pd_2$ cluster models which possess four or two center Pd atoms with symmetrical initial structures were investigated, respectively. As shown in Fig. S8, the adsorption Gibbs energy and enthalpy of **1a** on $Au_{16}Pd_4$ ($Au_{16}Pd_4$_**1a**_C) were comparable with those on $Au_{18}Pd_2$ ($Au_{18}Pd_2$_**1a**_C) (Fig. S10a,b), but the optimized structure of $Au_{16}Pd_4$ in $Au_{16}Pd_4$_**1a**_C was distorted while the counterpart using $Au_{18}Pd_2$ was not (Fig. S10c). Thus, we used the $Au_{18}Pd_2$ cluster model as the Au–Pd alloy nanoparticle model in the following calculation. Noteworthily, the adsorption Gibbs energy (ΔG) of $Au_{18}Pd_2$_**1a**_C is moderate, but that of Pd_{20}_**1a**_C is too large to proceed with the product desorption step in the direct metathesis reaction. In fact, the optimized structure of **1a** on Pd_{20} revealed the strong π-adsorption **1a** onto the facet of Pd_{20}, whereas the adsorption mode of **1a** on $Au_{18}Pd_2$ was totally different: almost no π-adsorption was observed while *S*-atom in **1a** was coordinated to Pd center. It was corroborated by the fact that natural bond orbital (NBO) charge of phenyl group in **1a** adsorbed on Pd_{20} increased compared with free **1a** probably due to π-back donation from Pd species to phenyl rings of **1a** but that on $Au_{18}Pd_2$ decreased possibly as the result of σ-donation from S atom to Pd atom (Fig. S11).

DFT Calculations for oxidative adducts of 1a on Au–Pd alloy nanoclusters

DFT calculations of **1a** oxidative adducts to $Au_{18}Pd_2$ were performed. As a result, even when an oxidative adduct of **1a** to the Pd center was used as an initial structure (Fig. S12a), the thiolate species moved onto Au species in a bridged adsorption manner after the geometry optimization (Fig. S12b), suggesting that spillover of thiolate or aryl species can easily occur after the oxidative addition step. Then, we calculated the four oxidative adduct structures after the spillover in bridged adsorption manners: whether it is Ph or SPh that moves onto Au and whether the Au species which a fragment moves onto are near or far from another Pd atom. The optimized structures and their ΔG were summarized in Fig. S13. The optimized structure of **1a** oxidative adduct in which SPh species moved onto Au near another Pd atom proved to be the most stable structure with a ΔG value of −27.1 kcal/mol,

which is also moderate value to proceed with the product desorption step in the direct metathesis reaction.

Synthesis of Thioethers

Condition A

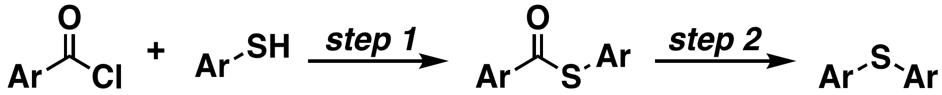

Step 1 (thioester synthesis): Acyl chlorides (6.3 mmol) and thiols (6.0 mmol) were dissolved in dehydrated dichloromethane (10 mL) in an Ar atmosphere. The solution was stirred at 0 °C for a few minutes. Triethylamine (12.0 mmol) was added to the solution in a dropwise manner followed by stirring at room temperature overnight. After the reaction, aqueous HCl solution (10 mL) was added and the organic layer was washed with brine (10 mL × 3) and deionized water (10 mL × 1) to afford analytically pure thioesters. The products were purified by silica-gel column chromatography if necessary.[S8]

Step 2 (thioester decarbonylation): Thioesters, a hydroxyapatite-supported Pd nanoparticle catalyst (Pd/HAP, Pd: 8 mol%), xylene (2 mL), and a Teflon-coated magnetic stir bar were placed in a Pyrex glass reactor (volume: ~20 mL) in an Ar atmosphere. The mixture was stirred at 160 °C for 24 h. After 24 h, Pd/HAP was removed by simple filtration. The crude thioethers were purified by silica-gel column chromatography using hexane and ethyl acetate as eluents, giving the pure products. **1b–1f**, **1h–1n** were synthesized under the Condition A.[S9]

Condition B

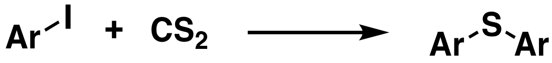

Aryl iodides (1 mmol), CuI (10 mol%), and a Teflon-coated magnetic stir bar were placed in a Pyrex glass reactor (volume: ~20 mL). In an Ar-filled glovebox, 1,8-diazabicyclo[5.4.0]undec-7-ene (DBU) (2 mmol) and toluene (1.5 mL) were added. Then, dropwise CS_2 (1 mmol) was added and heated at 100 °C for 12 h. After the reaction, ethyl acetate (5 mL) and deionized water (5 mL) were added, and the aqueous layer was extracted with brine (5 mL × 3). The crude thioethers were purified by silica-gel column chromatography using hexane and ethyl acetate as eluents, giving the pure products. **1p**, **1s**, and **1t** were synthesized under the Condition B.[S10]

Condition C

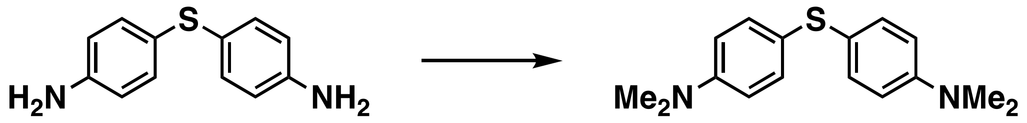

For the synthesis of bis[4-(*N*,*N*-dimethylamino)phenyl] sulfide (**1s**), *N*-methylation of bis(4-aminophenyl)sulfide (**1p**) was conducted. Bis(4-aminophenyl)sulfide and K_2CO_3 (10 eq.) were dissolved in acetone in an Ar atmosphere, then iodomethane (4.5 eq.) was added. The resulting mixture was heated under reflux conditions overnight. After the reaction, deionized water (30 mL) was added, and the products were extracted with ethyl acetate (10 mL × 3). The crude thioethers were purified by silica-gel column chromatography using hexane and ethyl acetate as eluents, giving the pure products.[S11]

Condition D

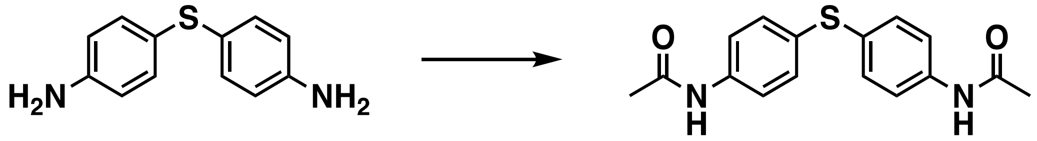

For the synthesis of *N*,*N′*-(thiodi-4,1-phenylene)bis acetamide (**1t**), *N*-amidation of bis(4-aminophenyl)sulfide (**1p**) was conducted. Bis(4-aminophenyl)sulfide was dissolved in dichloromethane (20 mL) at room temperature. Dropwise acetic anhydride (2.4 eq.) was added to afford precipitates immediately. After the reaction for 12 h, the precipitates were washed with ethyl acetate to afford the analytically pure product.[S12]

__Spectral Data of Thioether Substrates__

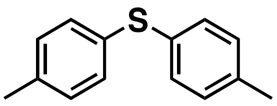

***p*-tolylsulfide** (CAS No. 6620-94-0, **1b**). 63% isolated yield. ^{1}H NMR (500 MHz, $CDCl_3$): δ 7.21–7.23 (m, 4H), 7.08–7.09 (m, 4H), 2.31 (s, 6H); ^{13}C NMR (125 MHz, $CDCl_3$): δ 137.0, 132.8, 131.2, 130.0, 21.2. MS (EI): *m/z* (%): 215 (17), 214 (100) [M^+], 213 (16), 199 (38), 198 (16), 184 (23), 181 (13), 105 (17), 91 (27), 65 (14).[S13]

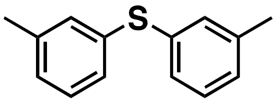

***m*-tolylsulfide** (CAS No. 3111-77-1, **1c**). >99% isolated yield. ^{1}H NMR (500 MHz, $CDCl_3$): δ 7.16–7.19 (m, 4H), 7.11–7.13 (m, 2H), 7.03–7.05 (m, 2H), 2.30 (s, 6H); ^{13}C NMR (125 MHz, $CDCl_3$): δ 139.1, 135.7, 131.7, 129.1, 128.2, 128.0, 21.4. MS (EI): *m/z* (%): 215 (16), 214 (100) [M^+], 213 (12), 199 (48), 198 (20), 197 (12), 184 (41), 165 (13), 105 (22), 91 (17), 77 (11), 65 (26), 63 (10).[S13]

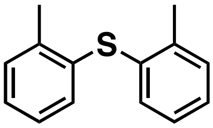

***o*-tolylsulfide** (CAS No. 4537-05-7, **1d**). 87% isolated yield. ^{1}H NMR (500 MHz, $CDCl_3$): δ 7.22–7.24 (m, 2H), 7.14–7.18 (m, 2H), 7.04–7.10 (m, 4H), 2.38 (s, 6H); ^{13}C NMR (125 MHz, $CDCl_3$): δ 138.9, 134.3, 131.1, 130.4, 127.1, 126.7, 20.4. MS (EI): *m/z* (%): 215 (16), 214 (100) [M^+], 199 (22), 197 (13), 184 (15), 166 (10), 165 (15), 123 (19), 122 (97), 121 (65), 105 (27), 92 (11), 91 (47), 89 (16), 78 (19), 77 (22), 65 (35), 63 (14), 51 (10).[S13]

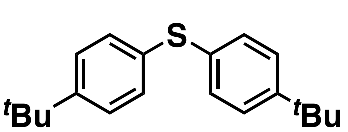

1,1′-thiobis[4-(1,1-dimethylethyl)benzene (CAS No. 52908-55-1, **1e**). 79% isolated yield. ^{1}H NMR (500 MHz, $CDCl_3$): δ 7.31–7.33 (m, 4H), 7.26–7.28 (m, 4H), 1.30 (s, 9H); ^{13}C NMR (125 MHz, $CDCl_3$): δ 152.7, 135.1, 133.3, 128.8, 37.1, 33.9. MS (EI): *m/z* (%): 299 (10), 298 (45) [M^+], 284 (21), 283 (100), 134 (12), 106 (22).[S13]

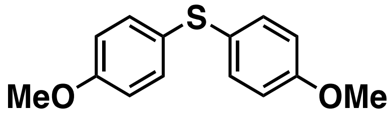

4,4'-dimethoxydiphenyl sulfide (CAS No. 3393-77-9, **1f**). 76% isolated yield. ^{1}H NMR (500 MHz, $CDCl_3$): δ 7.25–7.28 (m, 4H), 6.81–6.84 (m, 4H), 3.77 (s, 6H); ^{13}C NMR (125 MHz, $CDCl_3$): δ 159.1, 132.8, 127.5, 114.9, 55.4. MS (EI): *m/z* (%): 247 (17), 246 (100) [M^+], 231 (47), 215 (10), 214 (12), 203 (12), 199 (10), 188 (10), 171 (12), 115 (10).[S13]

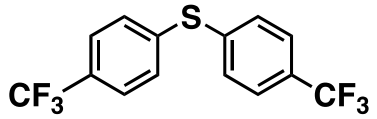

bis[(4-trifluoromethyl)phenyl]sulfide (CAS No. 90141-51-8, **1g**). 93% isolated yield. ^{1}H NMR (500 MHz, $CDCl_3$): δ 7.58–7.59 (m, 4H), 7.43–7.45 (m, 4H); ^{13}C NMR (125 MHz, $CDCl_3$): δ 139.7, 131.2, 129.8 (q, J = 32.3 Hz), 126.4 (q, J = 3.6 Hz), 124.0 (q, J = 270.6 Hz); ^{19}F NMR (470 MHz, $CDCl_3$): −61.9. MS (EI): *m/z* (%): 323 (16), 322 (100) [M^+], 303 (13), 301 (17), 253 (13), 252 (11), 233 (27), 184 (13).[S13]

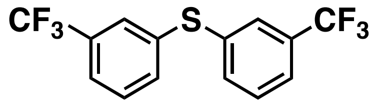

bis[(3-trifluoromethyl)phenyl]sulfide (CAS No. 1580-30-9, **1h**). 90% isolated yield. ^{1}H NMR (500 MHz, $CDCl_3$): δ 7.62 (m, 2H), 7.53–7.54 (m, 2H), 7.48–7.50 (m, 2H), 7.43–7.46 (m, 2H); ^{13}C NMR (125 MHz, $CDCl_3$): δ 136.4, 134.4, 132.1 (q, J = 33.4 Hz), 130.0, 127.8 (q, J = 3.6 Hz), 124.5 (q, J = 3.5 Hz), 123.7 (q, J = 270.6 Hz); ^{19}F NMR (470 MHz, $CDCl_3$): −62.1. MS (EI): *m/z* (%): 323 (15), 322 (100) [M^+], 303 (12), 301 (15), 233 (32), 184 (12).[S13]

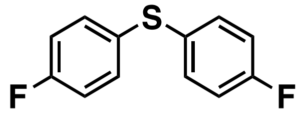

bis(4-fluorophenyl)sulfide (CAS No. 405-31-2, **1i**). 84% isolated yield. ^{1}H NMR (500 MHz, $CDCl_3$): δ 7.28–7.32 (m, 4H), 6.98–7.03 (m, 4H); ^{13}C NMR (125 MHz, $CDCl_3$): δ 162.2 (d, J = 246.8 Hz), 133.0 (d, J = 7.1 Hz), 131.1 (d, J = 3.6 Hz), 116.4 (d, J = 22.8 Hz); ^{19}F NMR (470 MHz, $CDCl_3$): −113.6. MS (EI): *m/z* (%): 224 (5), 223 (16), 222 (100) [M^+], 221 (49), 220 (20), 202 (18), 201 (15), 83 (23), 75 (14).[S13]

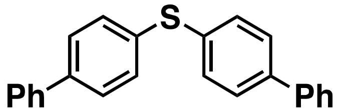

4-([1,1′-biphenyl]-4-ylthio)-1,1′-biphenyl (CAS No. 64554-57-0, **1k**). 72% isolated yield. ^{1}H NMR (500 MHz, $CDCl_3$): *δ* 7.54–7.59 (m, 8H), 7.42–7.46 (m, 8H), 7.34–7.37 (m, 2H); ^{13}C NMR (125 MHz, $CDCl_3$): *δ* 140.3, 140.1, 134.8, 131.4, 128.9, 127.9, 127.5, 127.0. MS (EI): *m/z* (%): 340 (8), 339 (26), 338 (100) [*M*$^+$], 337 (9), 321 (5), 306 (7), 261 (8), 260 (5), 184 (8), 169 (6), 152 (15), 115 (5).[S13]

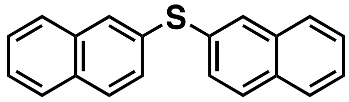

2-napthylsulfide (CAS No. 613-81-0, **1l**). 78% isolated yield. ^{1}H NMR (500 MHz, $CDCl_3$): *δ* 7.87 (m, 2H), 7.71–7.81 (m, 6H), 7.42–7.49 (m, 6H); ^{13}C NMR (125 MHz, $CDCl_3$): *δ* 134.0, 133.2, 132.5, 129.9, 129.0, 128.8, 127.9, 127.6, 126.8, 126.4. MS (EI): *m/z* (%): 287 (23), 286 (100) [*M*$^+$], 285 (54), 284 (28), 253 (20), 252 (27), 143 (10), 142 (14), 126 (14), 115 (12).[S13]

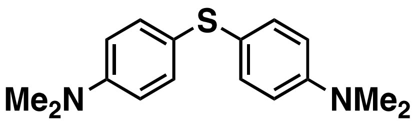

4,4′-thiobis[*N*,*N*-dimethylbenzenamine] (CAS No. 13604-44-9, **1n**). >99% isolated yield. ^{1}H NMR (500 MHz, $CDCl_3$): *δ* 7.22–7.25 (m, 4H), 6.63–6.66 (m, 4H), 2.92 (s, 12H); ^{13}C NMR (125 MHz, $CDCl_3$): *δ* 149.6, 132.6, 123.0, 113.1, 40.5. MS (EI): *m/z* (%): 273 (19), 272 (100) [*M*$^+$], 241 (19), 240 (88), 225 (18), 224 (13), 152 (19), 136 (17), 120 (15), 119 (11).[S13]

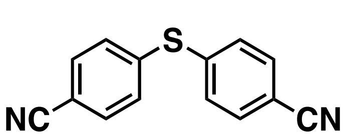

4,4′-thiobis[benzonitrile] (CAS No. 46836-99-1, **1o**). 53% isolated yield. ^{1}H NMR (500 MHz, $CDCl_3$): *δ* 7.61–7.63 (m, 4H), 7.41–7.43 (m, 4H); ^{13}C NMR (125 MHz, $CDCl_3$): *δ* 140.6, 133.0, 131.2, 118.2, 111.4. MS (EI): *m/z* (%): 237 (18), 236 (100) [*M*$^+$], 235 (46), 209 (15), 208 (18), 75 (12).[S13]

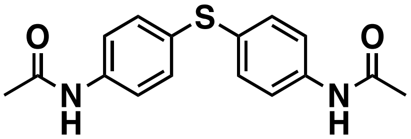

***N,N′*-(thiodi-4,1-phenylene)bis[acetamide]** (CAS No. 7355-56-8, **1q**). >99% isolated yield. ^{1}H NMR (500 MHz, dimethyl sulfoxide-d_6): δ 10.11 (s, 2H), 7.56–7.59 (m, 4H), 7.22–7.25 (m, 4H), 2.03 (s, 6H); ^{13}C NMR (125 MHz, dimethyl sulfoxide-d_6): δ 168.4, 138.7, 131.4, 128.6, 119.9, 24.0. MS (EI): *m/z* (%): 301 (21), 300 (100) [M^+], 259 (10), 258 (52), 217 (21), 216 (71), 215 (15), 184 (22), 183 (11), 124 (17).[S13]

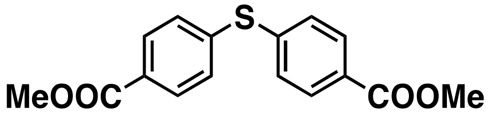

1,1′-dimethyl-4,4′-thiobis[benzoate] (CAS No. 14387-31-6, **1r**). 45% isolated yield. ^{1}H NMR (500 MHz, $CDCl_3$): δ 7.96–8.00 (m, 4H), 7.37–7.40 (m, 4H), 3.92 (s, 6H); ^{13}C NMR (125 MHz, $CDCl_3$): δ 166.5, 140.8, 130.5, 130.4, 129.1, 52.3. MS (EI): *m/z* (%): 303 (18), 302 (100) [M^+], 272 (17), 271 (93), 184 (40), 120 (19), 92 (15).[S13]

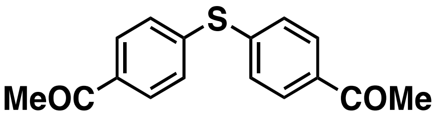

1,1′-(thiodi-4,1-phenylene)bis[ethanone] (CAS No. 2615-09-0, **1s**). 35% isolated yield. ^{1}H NMR (500 MHz, $CDCl_3$): δ 7.88–7.91 (m, 4H), 7.39–7.42 (m, 4H), 2.59 (s, 6H); ^{13}C NMR (125 MHz, $CDCl_3$): δ 197.1, 141.0, 135.9, 130.6, 129.2, 26.6. MS (EI): *m/z* (%): 271 (11), 270 (59) [M^+], 256 (17), 255 (100), 185 (13), 184 (18), 120 (11).[S13]

Spectral Data of Products

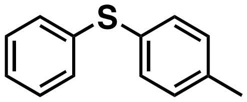

phenyl *p*-tolyl sulfide (CAS No. 3699-01-2, **1ab**): 77% GC yield. GC conditions and analysis: InertCap5 capillary column, 0.25 mm × 60 m, GL Science Inc.; carrier gas (N_2) flow rate, 1.7 mL·min^{-1}; initial column temp., 80 °C final column temp., 280 °C, progress rate, 20 °C·min^{-1} (10 min), 280 °C for 10 min, injection temp., 280 °C detection temp., 280 °C; retention time, 10.3 min; relative sensitivity for quantification (vs naphthalene, internal standard), 1.43 (calculated by calibration curve). MS (EI): *m/z* (%): 202 (5), 201 (16), 200 (100) [M^+], 199 (26), 186 (6), 185 (37), 184 (33), 167 (12), 165 (8), 152 (7), 99 (12), 91 (24), 77 (9), 65 (12), 51 (9), 45 (5), 39 (7).[S13]

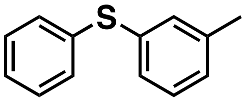

phenyl *m*-tolyl sulfide (CAS No. 13865-48-0, **1ac**): 78% GC yield. GC conditions and analysis: InertCap5 capillary column, 0.25 mm × 60 m, GL Science Inc.; carrier gas (N_2) flow rate, 1.7 mL·min^{-1}; initial column temp., 80 °C final column temp., 280 °C, progress rate, 20 °C·min^{-1} (10 min), 280 °C for 10 min, injection temp., 280 °C detection temp., 280 °C; retention time, 10.2 min; relative sensitivity for quantification (vs naphthalene, internal standard), 1.43 (estimated to be equal to that of **1ab**). MS (EI): *m/z* (%): 202 (5), 201 (15), 200 (100) [M^+], 199 (24), 186 (8), 185 (44), 184 (41), 167 (6), 165 (8), 152 (8), 99 (12), 91 (10), 77 (9), 65 (17), 63 (5), 51 (11).[S13]

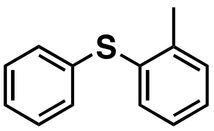

phenyl *o*-tolyl sulfide (CAS No. 13963-35-4, **1ad**): 71% GC yield. GC conditions and analysis: InertCap5 capillary column, 0.25 mm × 60 m, GL Science Inc.; carrier gas (N_2) flow rate, 1.7 mL·min^{-1}; initial column temp., 80 °C final column temp., 280 °C, progress rate, 20 °C·min^{-1} (10 min), 280 °C for 10 min, injection temp., 280 °C detection temp., 280 °C; retention time, 10.1 min; relative sensitivity for quantification (vs naphthalene, internal standard), 1.43 (estimated to be equal to that of **1ab**). MS (EI): *m/z* (%): 202 (6), 201 (16), 200 (100) [M^+], 199 (12), 197 (5), 185 (22), 184 (18), 167 (10), 166 (5), 165 (13), 152 (7), 123 (6), 122 (38), 121 (26), 99 (13), 91 (27), 89 (9), 78 (8), 77 (10), 65 (18), 63 (6), 51 (12).[S13]

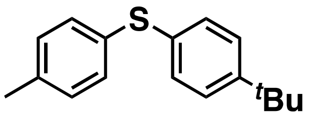

1-(1,1-dimethylethyl)-4-[(4-methylphenyl)thio]benzene (CAS No. 875713-05-6, **1be**): 79% GC yield. GC conditions and analysis: InertCap5 capillary column, 0.25 mm × 60 m, GL Science Inc.; carrier gas (N_2) flow rate, 1.7 mL·min^{-1}; initial column temp., 80 °C final column temp., 280 °C, progress rate, 20 °C·min^{-1} (10 min), 280 °C for 10 min, injection temp., 280 °C detection temp., 280 °C; retention time, 12.4 min; relative sensitivity for quantification (vs 1,3,5-trimethoxybenzene, internal standard), 2.77 (estimated by **1ab** and the effective carbon number concept). MS (EI): *m/z* (%): 257 (10), 256 (50) [M^+], 243 (6), 242 (18), 241 (100), 213 (7), 163 (5), 123 (21), 118 (6), 117 (11), 115 (6), 106 (9), 105 (5), 91 (8), 79 (5).[S13]

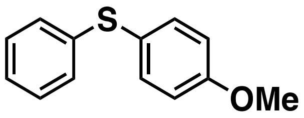

1-methoxy-4-(phenylthio)benzene (CAS No. 5633-57-8, **1af**): 78% GC yield (81% isolated yield). GC conditions and analysis: InertCap5 capillary column, 0.25 mm × 60 m, GL Science Inc.; carrier gas (N_2) flow rate, 1.7 mL·min^{-1}; initial column temp., 80 °C final column temp., 280 °C, progress rate, 20 °C·min^{-1} (10 min), 280 °C for 10 min, injection temp., 280 °C detection temp., 280 °C; retention time, 12.0 min; relative sensitivity for quantification (vs naphthalene, internal standard), 1.33 (estimated by **1ab** and the effective carbon number concept). ^{1}H NMR (500 MHz, $CDCl_3$): δ 7.38–7.43 (m, 2H), 7.19–7.25 (m, 2H), 7.10–7.18 (m, 3H), 6.86–6.91 (m, 2H), 3.81 (s, 3H); ^{13}C NMR (125 MHz, $CDCl_3$): δ 159.8, 138.6, 135.3, 128.9, 128.2, 125.7, 124.3, 115.0, 55.4. MS (EI): *m/z* (%): 218 (6), 217 (15), 216 (100) [M^+], 215 (11), 202 (8), 201 (55), 200 (5), 185 (8), 184 (7), 173 (8), 172 (5), 171 (8), 139 (5), 129 (15), 128 (6), 77 (10), 51 (9).[S13]

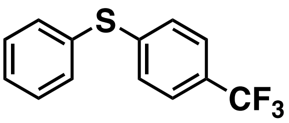

1-(phenylthio)-4-(trifluoromethyl)benzene (CAS No. 53451-90-4, **1ag**): 75% GC yield. GC conditions and analysis: InertCap5 capillary column, 0.25 mm × 60 m, GL Science Inc.; carrier gas (N_2) flow rate, 1.7 mL·min^{-1}; initial column temp., 80 °C final column temp., 280 °C, progress rate, 10 °C·min^{-1} (20 min), 280 °C for 10 min, injection temp., 280 °C detection temp., 280 °C; retention time, 14.1 min; relative sensitivity for quantification (vs naphthalene, internal standard), 1.33 (estimated by **1ab** and the effective carbon number concept). MS (EI): *m/z* (%): 256 (6), 255 (15), 254

(100) [M^+], 253 (15), 235 (7), 233 (23), 186 (6), 185 (36), 184 (28), 109 (5), 77 (14), 69 (6), 65 (6), 51 (17), 50 (5).[S13]

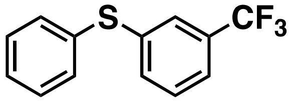

1-(phenylthio)-3-(trifluoromethyl)benzene (CAS No. 2715-07-3, **1ah**): 72% GC yield. GC conditions and analysis: InertCap5 capillary column, 0.25 mm × 60 m, GL Science Inc.; carrier gas (N_2) flow rate, 1.7 mL·min^{-1}; initial column temp., 80 °C final column temp., 280 °C, progress rate, 10 °C·min^{-1} (20 min), 280 °C for 10 min, injection temp., 280 °C detection temp., 280 °C; retention time, 13.8 min; relative sensitivity for quantification (vs naphthalene, internal standard), 1.33 (estimated by **1ab** and the effective carbon number concept). MS (EI): *m/z* (%): 256 (6), 255 (16), 254 (100) [M^+], 253 (13), 235 (6), 234 (6), 233 (25), 186 (5), 185 (31), 184 (25), 109 (5), 77 (15), 65 (6), 51 (16).[S13]

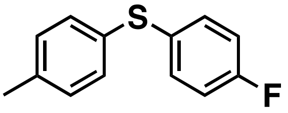

1-fluoro-4-[(4-methylphenyl)thio]benzene (CAS No. 42917-47-5, **1bi**): 81% GC yield. GC conditions and analysis: InertCap5 capillary column, 0.25 mm × 60 m, GL Science Inc.; carrier gas (N_2) flow rate, 1.7 mL·min^{-1}; initial column temp., 80 °C final column temp., 280 °C, progress rate, 20 °C·min^{-1} (10 min), 280 °C for 10 min, injection temp., 280 °C detection temp., 280 °C; retention time, 10.1 min; relative sensitivity for quantification (vs dodecane, internal standard), 1.06 (estimated by **1ab** and the effective carbon number concept). MS (EI): *m/z* (%): 220 (5), 219 (15), 218 (100) [M^+], 217 (22), 204 (5), 203 (32), 202 (26), 185 (17), 183 (10), 108 (11), 98 (6), 91 (27), 83 (6), 77 (5), 65 (13), 63 (5).[S13]

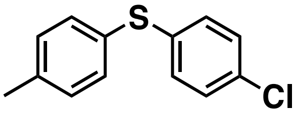

1-chloro-4-[(4-methylphenyl)thio]benzene (CAS No. 22865-55-0, **1bj**): 78% GC yield. GC conditions and analysis: InertCap5 capillary column, 0.25 mm × 60 m, GL Science Inc.; carrier gas (N_2) flow rate, 1.7 mL·min^{-1}; initial column temp., 80 °C final column temp., 280 °C, progress rate, 20 °C·min^{-1} (10 min), 280 °C for 10 min, injection temp., 280 °C detection temp., 280 °C; retention time, 11.4 min; relative sensitivity for quantification (vs naphthalene, internal standard), 1.41 (estimated by **1ab** and the effective carbon number concept). MS (EI): *m/z* (%): 237 (5), 236 (36), 235

(17), 234 (100) [M^+], 233 (8), 219 (7), 201 (14), 200 (6), 199 (31), 198 (17), 197 (10), 185 (6), 184 (43), 166 (7), 165 (10), 116 (5), 108 (6), 99 (7), 98 (13), 91 (29), 89 (5), 77 (7), 75 (5), 65 (15), 63 (7), 51 (5), 45 (7), 39 (8).[S14]

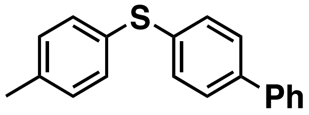

4-[(4-methylphenyl)thio]-1,1′-biphenyl (CAS No. 1361950-30-2, **1bk**): 63% GC yield (55% isolated yield). GC conditions and analysis: InertCap5 capillary column, 0.25 mm × 60 m, GL Science Inc.; carrier gas (N_2) flow rate, 1.7 mL·min^{-1}; initial column temp., 80 °C final column temp., 280 °C, progress rate, 20 °C·min^{-1} (10 min), 280 °C for 10 min, injection temp., 280 °C detection temp., 280 °C; retention time, 17.0 min; relative sensitivity for quantification (vs 1,3,5-trimethoxybenzene, internal standard), 3.10 (estimated by **1ab** and the effective carbon number concept). ^{1}H NMR (500 MHz, $CDCl_3$): δ 7.52–7.56 (m, 2H), 7.46–7.50 (m, 2H), 7.38–7.43 (m, 2H), 7.29–7.36 (m, 5H), 7.12–7.17 (m, 2H), 2.34 (s, 3H); ^{13}C NMR (125 MHz, $CDCl_3$): δ 140.4, 139.3, 137.7, 136.3, 132.4, 131.2, 130.1, 130.0, 128.8, 127.7, 127.3, 126.9, 21.1. MS (EI): *m/z* (%): 278 (7), 277 (22), 276 (100) [M^+], 275 (14), 261 (20), 260 (16), 244 (5), 243 (5), 228 (6), 184 (8), 152 (12), 115 (5), 91 (9), 77(5), 65 (5).[S13]

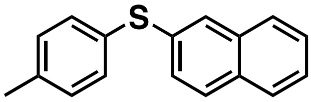

2-[(4-methylphenyl)thio]naphthalene (CAS No. 52258-16-9, **1bl**): 90% GC yield (57% isolated yield). GC conditions and analysis: InertCap5 capillary column, 0.25 mm × 60 m, GL Science Inc.; carrier gas (N_2) flow rate, 1.7 mL·min^{-1}; initial column temp., 80 °C final column temp., 280 °C, progress rate, 20 °C·min^{-1} (10 min), 280 °C for 10 min, injection temp., 280 °C detection temp., 280 °C; retention time, 14.6 min; relative sensitivity for quantification (vs 1,3,5-trimethoxybenzene, internal standard), 2.74 (estimated by **1ab** and the effective carbon number concept). ^{1}H NMR (500 MHz, $CDCl_3$): δ 7.67–7.79 (m, 4H), 7.40–7.47 (m, 2H), 7.30–7.37 (m, 3H), 7.12–7.16 (m, 2H), 2.35 (s, 3H); ^{13}C NMR (125 MHz, $CDCl_3$): δ 137.6, 134.3, 133.8, 132.1, 132.0, 131.4, 130.1, 128.7, 128.4, 127.9, 127.7, 127.4, 126.5, 125.9, 21.1. MS (EI): *m/z* (%): 252 (6), 251 (20), 250 (100) [M^+], 249 (19), 236 (8), 235 (39), 234 (38), 217 (8), 215 (8), 202 (13), 127 (5), 126 (5), 125 (5), 124 (10), 117 (8), 115 (12), 91 (6), 77 (5).[S14]

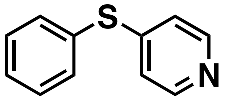

4-(phenylthio)pyridine (CAS No. 33399-48-3, **1am**): 26% GC yield. GC conditions and analysis: InertCap5 capillary column, 0.25 mm × 60 m, GL Science Inc.; carrier gas (N_2) flow rate, 1.7 mL·min^{-1}; initial column temp., 80 °C final column temp., 280 °C, progress rate, 20 °C·min^{-1} (10 min), 280 °C for 10 min, injection temp., 280 °C detection temp., 280 °C; retention time, 10.1 min; relative sensitivity for quantification (vs 1,3,5-trimethoxybenzene, internal standard), 1.65 (calculated by calibration curve). ^{1}H NMR (500 MHz, $CDCl_3$): δ 8.33–8.36 (m, 2H), 7.54–7.58 (m, 2H), 7.44–7.48 (m, 3H), 6.92–6.95 (m, 2H); ^{13}C NMR (125 MHz, $CDCl_3$): δ 150.3, 149.5, 135.2, 129.9, 129.7, 129.4, 120.8. MS (EI): *m/z* (%): 189 (5), 188 (16), 187 (100) [M^+], 186 (73), 160 (7), 154 (7), 115 (15), 109 (6), 92 (5), 78 (8), 77 (9), 65 (6), 51 (34), 50 (5), 39 (6).[S13]

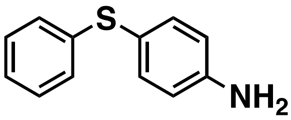

4-(phenylthio)benzenamine (CAS No. 1135-14-4, **1an**): 75% GC yield (80% isolated yield). GC conditions and analysis: InertCap5 capillary column, 0.25 mm × 60 m, GL Science Inc.; carrier gas (N_2) flow rate, 1.7 mL·min^{-1}; initial column temp., 80 °C final column temp., 280 °C, progress rate, 20 °C·min^{-1} (10 min), 280 °C for 10 min, injection temp., 280 °C detection temp., 280 °C; retention time, 12.1 min; relative sensitivity for quantification (vs naphthalene, internal standard), 1.30 (estimated by **1ab** and the effective carbon number concept). ^{1}H NMR (500 MHz, $CDCl_3$): δ 7.28–7.33 (m, 2H), 7.17–7.22 (m, 2H), 7.06–7.14 (m, 3H), 6.63–6.68 (m, 2H), 3.68 (brs, 2H); ^{13}C NMR (125 MHz, $CDCl_3$): δ 147.0, 139.7, 136.1, 128.8, 127.3, 125.2, 120.4, 115.9. MS (EI): *m/z* (%): 203 (5), 202 (16), 201 (100) [M^+], 200 (53), 199 (8), 184 (6), 169 (19), 168 (5), 167 (5), 124 (16), 99 (5), 80 (17), 65 (8), 51 (7), 39 (6).[S13]

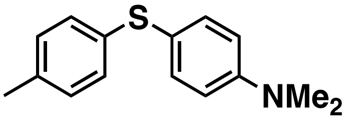

***N*,*N*-dimethyl-4-[(4-methylphenyl)thio]benzenamine** (CAS No. 2849-63-2, **1bo**): 63% GC yield (23% isolated yield). GC conditions and analysis: InertCap5 capillary column, 0.25 mm × 60 m, GL Science Inc.; carrier gas (N_2) flow rate, 1.7 mL·min^{-1}; initial column temp., 80 °C final column temp., 280 °C, progress rate, 20 °C·min^{-1} (10 min), 280 °C for 10 min, injection temp., 280 °C detection temp., 280 °C; retention time, 13.6 min; relative sensitivity for quantification (vs 1,3,5-trimethoxybenzene, internal standard), 2.39 (estimated by **1ab** and the effective carbon number

concept). ^{1}H NMR (500 MHz, $CDCl_3$): δ 7.33–7.38 (m, 2H), 7.00–7.07 (m, 4H), 6.66–6.70 (m, 2H), 2.96 (s, 6H), 2.27 (s, 3H); ^{13}C NMR (125 MHz, $CDCl_3$): δ 150.4, 136.2, 135.5, 135.1, 129.4, 127.8, 118.7, 113.0, 40.3, 20.9. MS (EI): *m/z* (%): 245 (6), 244 (18), 243 (100) [M^+], 242 (14), 228 (8), 227 (10), 226 (6), 212 (12), 211 (38), 210 (33), 195 (9), 184 (10), 152 (11), 136 (5), 121 (7), 120 (6), 105 (5), 91 (5), 77 (5), 65 (5).[S13]

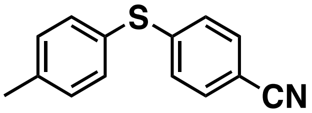

4-[(4-methylphenyl)thio]benzonitrile (CAS No. 104128-50-9, **1bp**): 80% GC yield (79% isolated yield). GC conditions and analysis: InertCap5 capillary column, 0.25 mm × 60 m, GL Science Inc.; carrier gas (N_2) flow rate, 1.7 mL·min^{-1}; initial column temp., 80 °C final column temp., 280 °C, progress rate, 20 °C·min^{-1} (10 min), 280 °C for 10 min, injection temp., 280 °C detection temp., 280 °C; retention time, 12.7 min; relative sensitivity for quantification (vs 1,3,5-trimethoxybenzene, internal standard), 2.17 (calculated by calibration curve). ^{1}H NMR (500 MHz, $CDCl_3$): δ 7.43–7.47 (m, 2H), 7.39–7.43 (m, 2H), 7.22–7.26 (m, 2H), 7.09–7.14 (m, 2H), 2.40 (s, 3H); ^{13}C NMR (125 MHz, $CDCl_3$): δ 146.6, 139.9, 134.9, 132.3, 130.7, 126.8, 126.7, 118.9, 108.3, 22.0. MS (EI): *m/z* (%): 227 (6), 226 (17), 225 (100) [M^+], 224 (23), 211 (5), 210 (32), 209 (18), 192 (22), 190 (6), 165 (5), 111 (10), 91 (39), 89 (6), 77 (8), 65 (19), 63 (8), 51 (6).[S13]

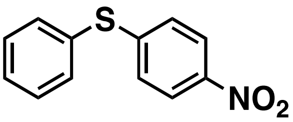

4-nitrophenyl phenyl sulfide (CAS No. 1952-97-6, **1aq**): 89% GC yield (93% isolated yield). GC conditions and analysis: InertCap5 capillary column, 0.25 mm × 60 m, GL Science Inc.; carrier gas (N_2) flow rate, 1.7 mL·min^{-1}; initial column temp., 80 °C final column temp., 280 °C, progress rate, 20 °C·min^{-1} (10 min), 280 °C for 10 min, injection temp., 280 °C detection temp., 280 °C; retention time, 12.6 min; relative sensitivity for quantification (vs naphthalene, internal standard), 1.22 (estimated by **1ab** and the effective carbon number concept). ^{1}H NMR (500 MHz, $CDCl_3$): δ 8.03–8.09 (m, 2H), 7.51–7.57 (m, 2H), 7.43–7.48 (m, 3H), 7.15–7.20 (m, 2H); ^{13}C NMR (125 MHz, $CDCl_3$): δ 148.5, 145.3, 134.7, 130.4, 130.0, 129.7, 126.7, 124.0. MS (EI): *m/z* (%): 233 (6), 232 (14), 231 (100) [M^+], 201 (20), 186 (5), 185 (17), 184 (64), 183 (5), 152 (13), 139 (6), 115 (6), 109 (6), 92 (6), 77 (6), 69 (6), 65 (8), 51 (8), 50 (5), 45 (7).[S13]

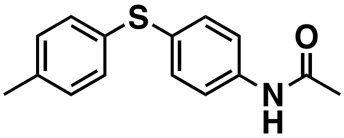

***N*-[4-[(4-methylphenyl)thio]phenyl]acetamide** (CAS No. 339096-10-5, **1br**): 64% GC yield (53% isolated yield). GC conditions and analysis: InertCap5 capillary column, 0.25 mm × 60 m, GL Science Inc.; carrier gas (N_2) flow rate, 1.7 mL·min^{-1}; initial column temp., 80 °C final column temp., 280 °C, progress rate, 20 °C·min^{-1} (10 min), 280 °C for 10 min, injection temp., 280 °C detection temp., 280 °C; retention time, 16.6 min; relative sensitivity for quantification (vs 1,3,5-trimethoxybenzene, internal standard), 2.54 (estimated by **1ab** and the effective carbon number concept). ^{1}H NMR (500 MHz, $CDCl_3$): δ 7.70 (brs, 1H), 7.40–7.46 (m, 2H), 7.23–7.28 (m, 2H), 7.19–7.23 (m, 2H), 7.07–7.12 (m, 2H), 2.31 (s, 3H), 2.14 (s, 3H); ^{13}C NMR (125 MHz, $CDCl_3$): δ 168.6, 137.1, 136.9, 132.3, 131.7, 131.4, 131.1, 130.0, 120.7, 24.5, 21.1. MS (EI): *m/z* (%): 259 (6), 258 (18), 257 (100) [M^+], 217 (5), 216 (17), 215 (78), 214 (26), 200 (23), 199 (10), 184 (5), 183 (18), 182 (8), 124 (7), 91 (6), 65 (8).[S13]

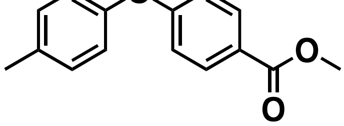

methyl 4-[(4-methylphenyl)thio]benzoate (CAS No. 1818399-49-3, **1bs**): 80% GC yield (62% isolated yield). GC conditions and analysis: InertCap5 capillary column, 0.25 mm × 60 m, GL Science Inc.; carrier gas (N_2) flow rate, 1.7 mL·min^{-1}; initial column temp., 80 °C final column temp., 280 °C, progress rate, 20 °C·min^{-1} (10 min), 280 °C for 10 min, injection temp., 280 °C detection temp., 280 °C; retention time, 13.3 min; relative sensitivity for quantification (vs 1,3,5-trimethoxybenzene, internal standard), 2.27 (estimated by **1ab** and the effective carbon number concept). ^{1}H NMR (500 MHz, $CDCl_3$): δ 7.85–7.88 (m, 2H), 7.38–7.42 (m, 2H), 7.19–7.23 (m, 2H), 7.12–7.16 (m, 2H), 3.88 (s, 3H), 2.39 (s, 3H); ^{13}C NMR (125 MHz, $CDCl_3$): δ 166.7, 145.4, 139.2, 134.4, 130.5, 130.0, 128.1, 127.0, 126.7, 52.0, 21.3. MS (EI): *m/z* (%): 260 (6), 259 (16), 258 (100) [M^+], 228 (10), 227 (59), 225 (5), 199 (20), 198 (7), 197 (7), 185 (6), 184 (43), 165 (5), 113 (5), 91 (9), 79 (5), 77 (5), 65 (6).[S13]

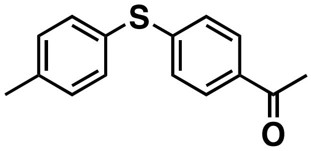

1-[4-[(4-methylphenyl)thio]phenyl]ethanone (CAS No. 99433-27-9, **1bt**): 80% GC yield (71% isolated yield) (The isolated yield was shown after the subtraction of the amounts of residual solvents calculated from ^{1}H NMR). GC conditions and analysis: InertCap5 capillary column, 0.25 mm × 60 m,

GL Science Inc.; carrier gas (N_2) flow rate, 1.7 mL·min^{-1}; initial column temp., 80 °C final column temp., 280 °C, progress rate, 20 °C·min^{-1} (10 min), 280 °C for 10 min, injection temp., 280 °C detection temp., 280 °C; retention time, 12.4 min; relative sensitivity for quantification (vs 1,3,5-trimethoxybenzene, internal standard), 2.27 (calculated by calibration curve). ^{1}H NMR (500 MHz, $CDCl_3$): δ 7.77–7.81 (m, 2H), 7.38–7.43 (m, 2H), 7.20–7.24 (m, 2H), 7.13–7.17 (m, 2H), 2.53 (s, 3H), 2.39 (s, 3H); ^{13}C NMR (125 MHz, $CDCl_3$): δ 197.1, 145.9, 139.3, 134.5, 134.1, 130.5, 128.8, 127.9, 126.7, 26.4, 21.3. MS (EI): *m/z* (%): 243 (12), 242 (72) [M^+], 229 (6), 228 (16), 227 (100), 199 (9), 197 (6), 185 (7), 184 (47), 165 (6), 152 (5), 113 (5), 79 (5), 77 (5), 65 (6).[S14]

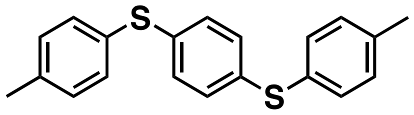

1,4-bis[(4-methylphenyl)thio]benzene (CAS No. 55709-45-0, **1bab**): 29% GC yield. GC conditions and analysis: InertCap5 capillary column, 0.25 mm × 60 m, GL Science Inc.; carrier gas (N_2) flow rate, 1.7 mL·min^{-1}; initial column temp., 80 °C final column temp., 280 °C, progress rate, 20 °C·min^{-1} (10 min), 280 °C for 20 min, injection temp., 280 °C detection temp., 280 °C; retention time, 22.4 min; relative sensitivity for quantification (vs 1,3,5-trimethoxybenzene, internal standard), 3.20 (estimated by **1ab** and the effective carbon number concept). MS (EI): *m/z* (%): 324 (12), 323 (23), 322 (100) [M^+], 201 (5), 200 (13), 199 (86), 198 (9), 197 (13), 184 (23), 166 (7), 165 (8), 91 (7), 65 (8).[S13]

Supplementary References

(S1) J. W. Cook Jr. and D. E. Sayers, *J. Appl. Phys.*, 1981, **52**, 5024.

(S2) A. L. Ankudinov and J. J. Rehr, *Phys. Rev. B*, 1997, **56**, R1712.

(S3) Y. Sakamoto, F. L. Chen and R. McNicholl, *J. Alloys Compd.*, 1993, **192**, 145.

(S4) A. G. Christy and S. M. Clark, *Phys. Rev. B*, 1995, **52**, 9259.

(S5) M. Ellner, K. Kolatschek and B. Predel, *J. Less-Common Met.*, 1991, **170**, 171.

(S6) (a) R. N. Dhital, C. Kamonsatikul, E. Somsook, K. Bobuatong, M. Ehara, S. Karanjit and H. Sakurai, *J. Am. Chem. Soc.*, 2012, **134**, 20250; (b) X.-F. Lang, P.-G. Yin, T.-T. You and L. Guo, *ChemPhysChem*, 2012, **13**, 237.

(S7) (a) R. Miyazaki, X. Jin, D. Yoshii, T. Yatabe, T. Yabe, N. Mizuno, K. Yamaguchi and J. Hasegawa, *Catal. Sci. Technol.*, 2021, **11**, 3333; (b) D. Takei, T. Yatabe, T. Yabe, R. Miyazaki, J. Hasegawa and K. Yamaguchi, *JACS Au*, 2022, **2**, 394.

(S8) H. Cao, X. Liu, F. Bie, Y. Shi, Y. Han, P. Yan, M. Szostak and C. Liu, *J. Org. Chem.*, 2021, **86**, 10829.

(S9) T. Matsuyama, T. Yatabe and K. Yamaguchi, *Org. Biomol. Chem.*, 2024, **22**, 579.

(S10) P. Zhao, H. Yin, H. Gao and C. Xi, *J. Org. Chem.*, 2013, **78**, 5001.

(S11) P. An, Z.-F. Shi, W. Dou, X.-P. Cao and H.-L. Zhang, *Org. Lett.*, 2010, **12**, 4364.

(S12) B. Wang, C. Jiang, J. Qian, S. Zhang, X. Jia and Y. Yuan, *Org. Biomol. Chem.*, 2018, **16**, 101.

(S13) T. Matsuyama, T. Yatabe, T. Yabe and K. Yamaguchi, *Catal. Sci. Technol.*, 2024, **14**, 76.

(S14) D. Liu, H.-X. Ma, P. Fang and T.-S. Mei, *Angew. Chem. Int. Ed.*, 2019, **58**, 5033.

Supplementary Figures

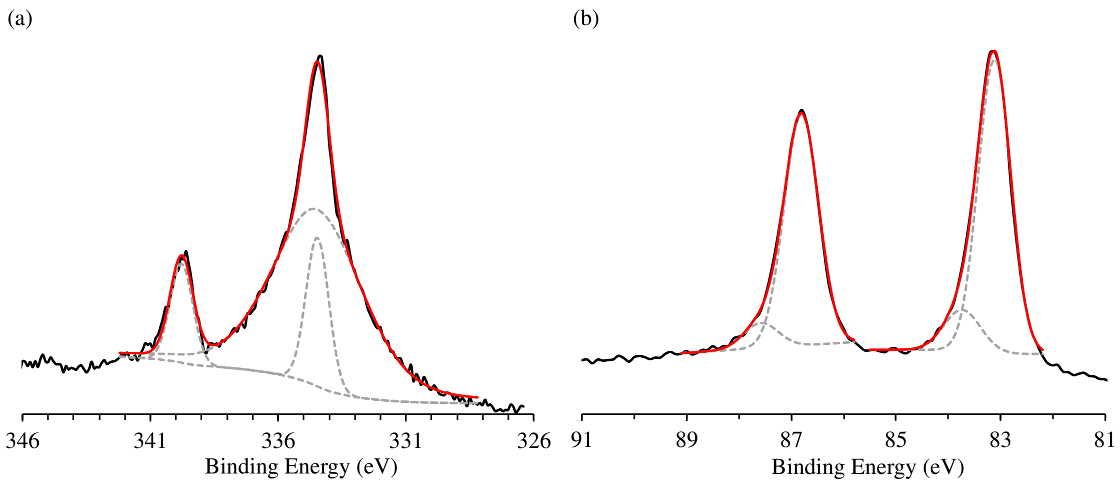

Fig. S1 (a) Pd 3d and (b) Au 4f XPS spectra of $Au_{4.4}$–Pd_1/TiO_2. Gray broken lines and red broken lines indicate the deconvoluted signals and the sum of these lines. Black lines indicate the data plots.

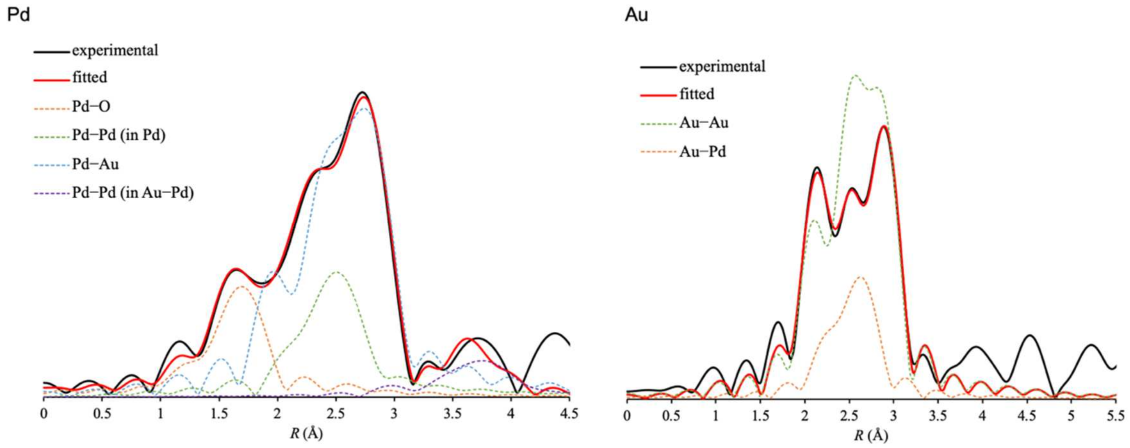

Fig. S2 Fourier-transformed k^3-weighted (a) Pd K-edge and (b) Au L_{III}-edge EXAFS spectra of $Au_{4.4}$–Pd_1/TiO_2. Experimental data and fitted data are shown. All R-space spectra are shown without phase correction.

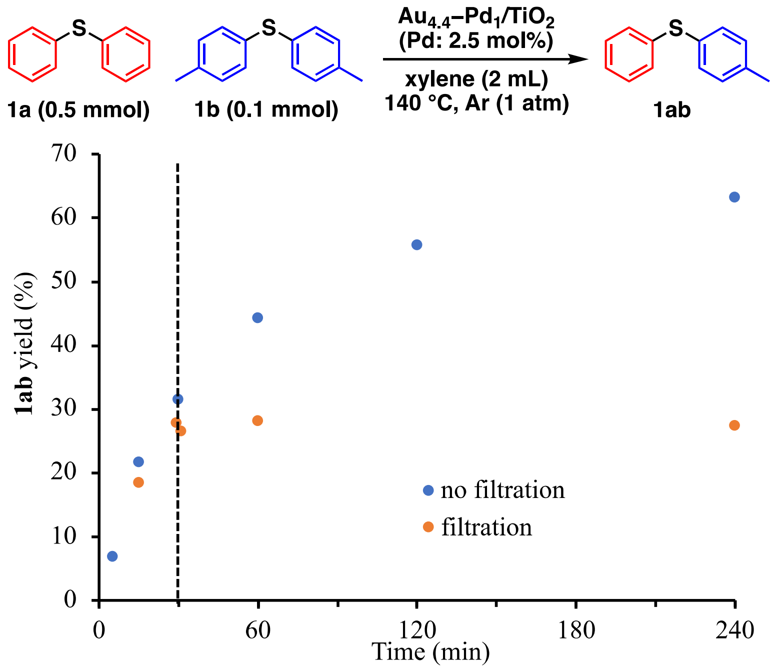

Fig. S3 Effect of removal of $Au_{4.4}$–Pd_1/TiO_2 catalyst on the metathesis between **1a** and **1b**. Reaction conditions: **1a** (0.5 mmol), **1b** (0.1 mmol), $Au_{4.4}$–Pd_1/TiO_2 (Pd: 2.5 mol%), xylene (2 mL), Ar (1 atm), 140 °C. Yields were determined by GC analysis using 1,3,5-trimethoxybenzene as an internal standard.

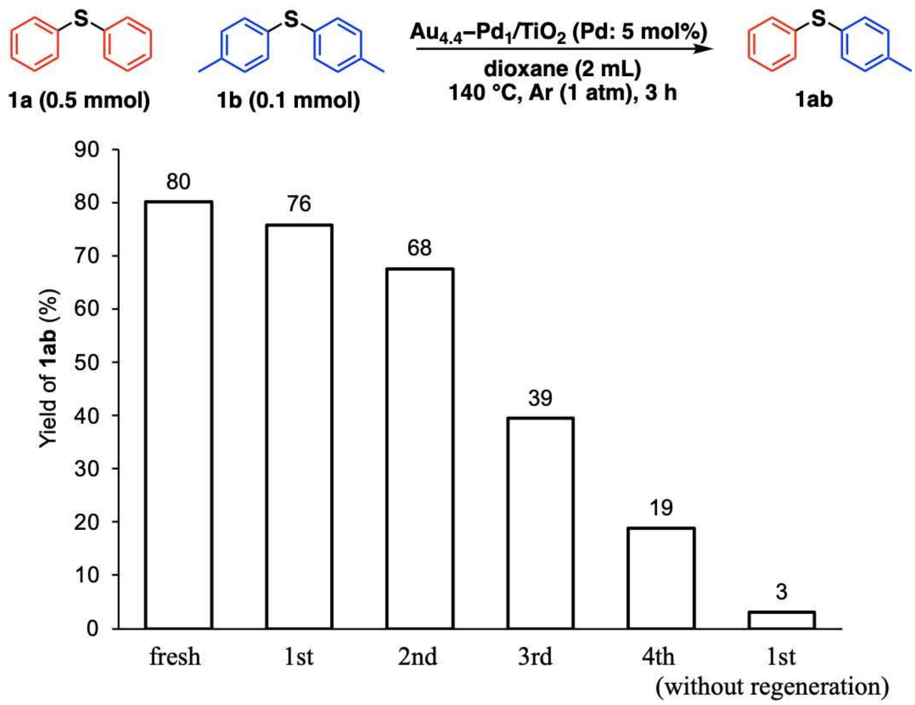

Fig. S4 $Au_{4.4}$–Pd_1/TiO_2 reuse experiments. The yields after 3 h are shown. The reaction conditions are indicated in this figure.

(a) (b)

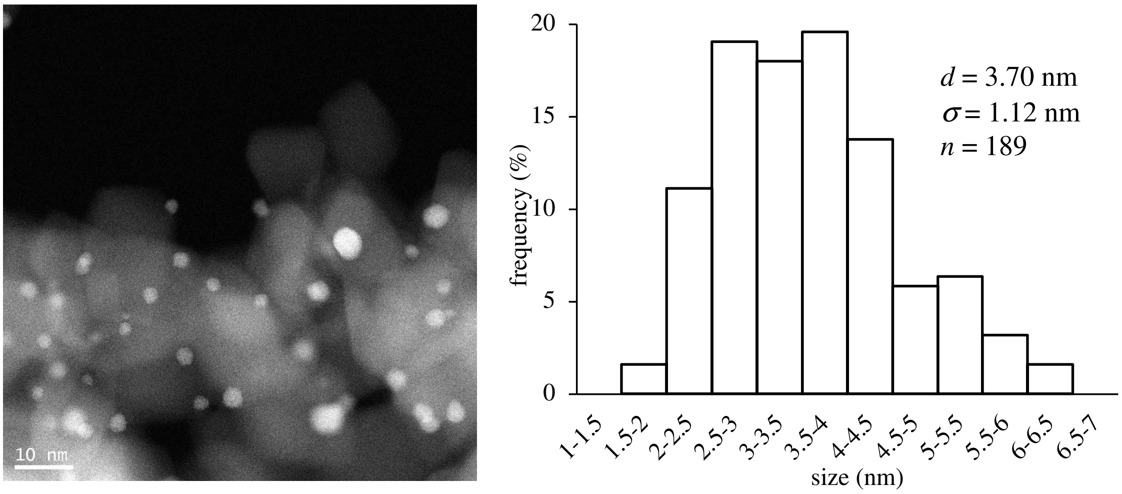

Fig. S5 (a) HAADF-STEM image of $Au_{4.4}$–Pd_1/TiO_2 after the 1st use and (b) particle size distribution of $Au_{4.4}$–Pd_1/TiO_2 after the 1st use.

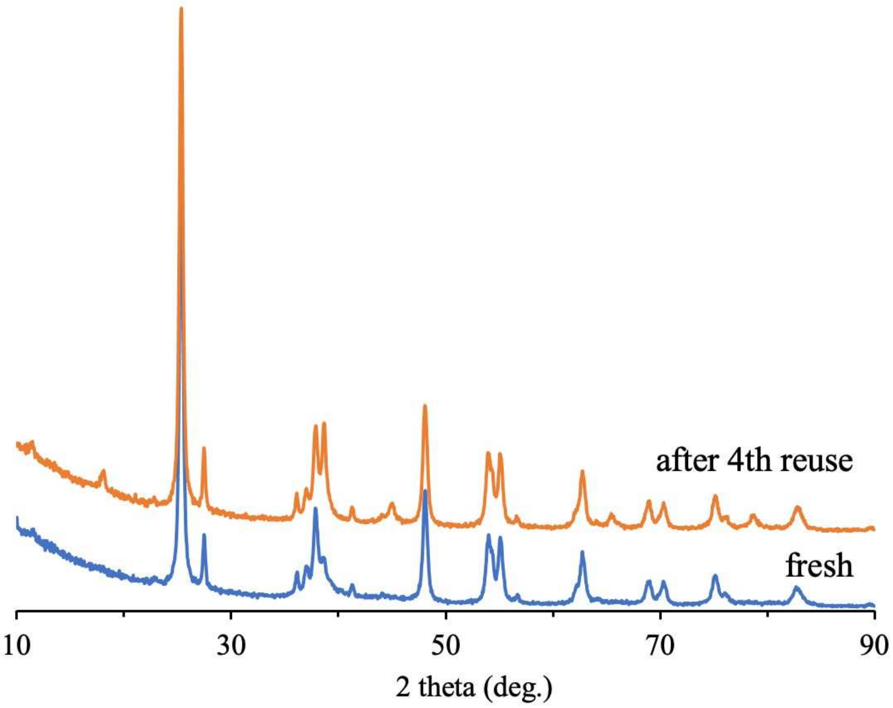

Fig. S6 XRD patterns of $Au_{4.4}$–Pd_1/TiO_2 before use and after the 4th reuse.

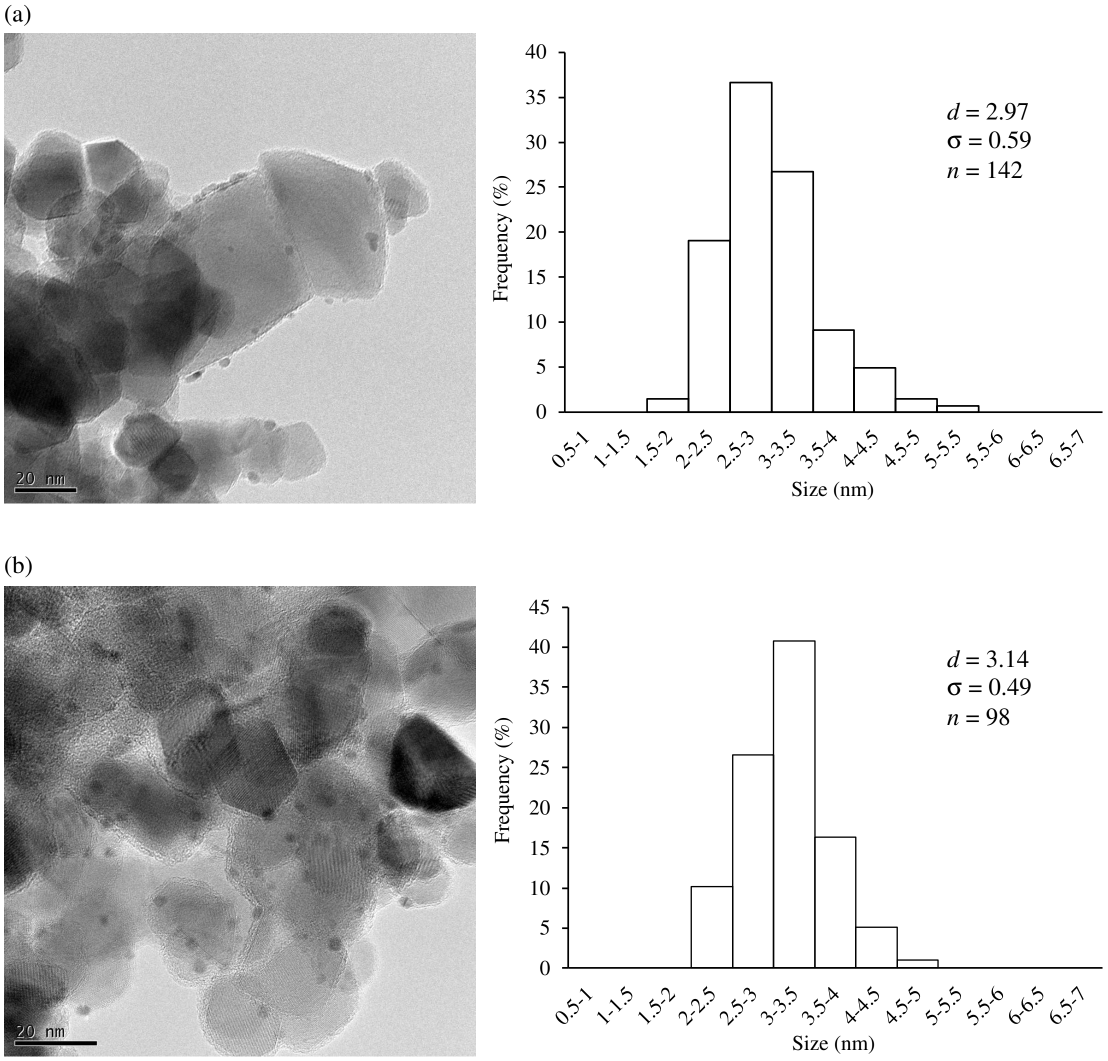

Fig. S7 TEM images and particle size distributions of (a) Pd_1/TiO_2 and (b) $Au_{1.4}$–Pd_1/TiO_2.

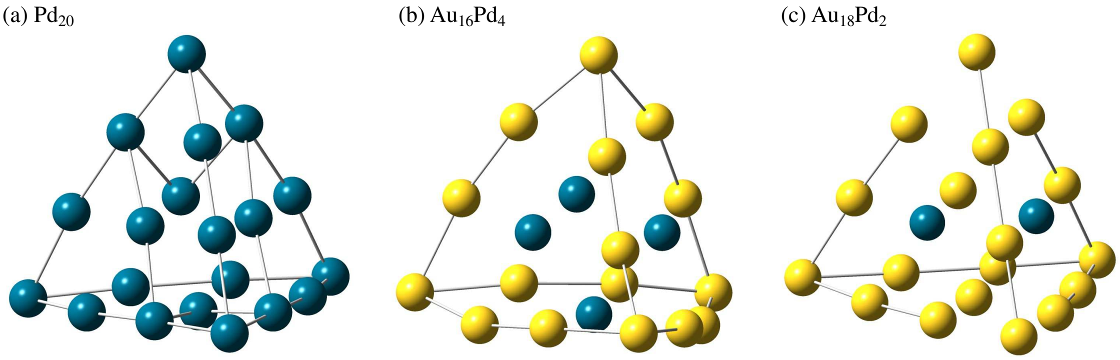

Fig. S8 Optimized structures of (a) Pd_{20}, (b) $Au_{16}Pd_4$, and (c) $Au_{18}Pd_2$ cluster models

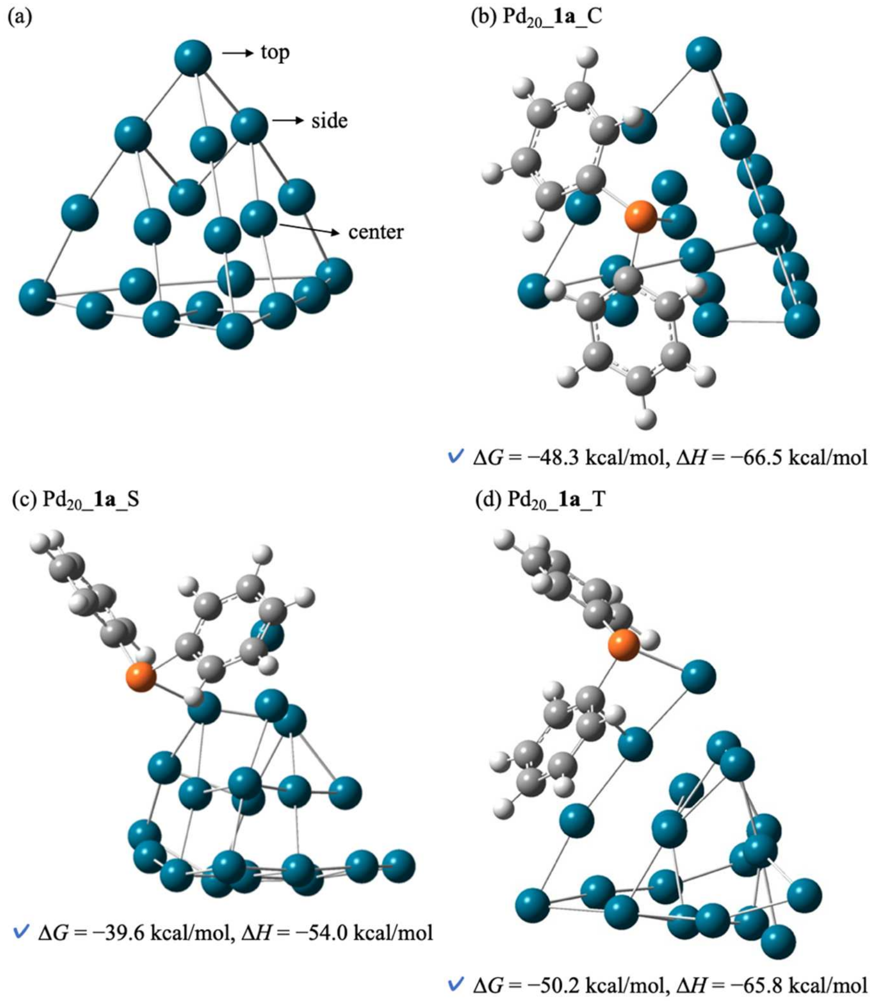

Fig. S9 Summary of adsorption energies and optimized structures of (a) Pd_{20}, (b) Pd_{20}_**1a**_C, (c) Pd_{20}_**1a**_S, and (d) Pd_{20}_**1a**_T.

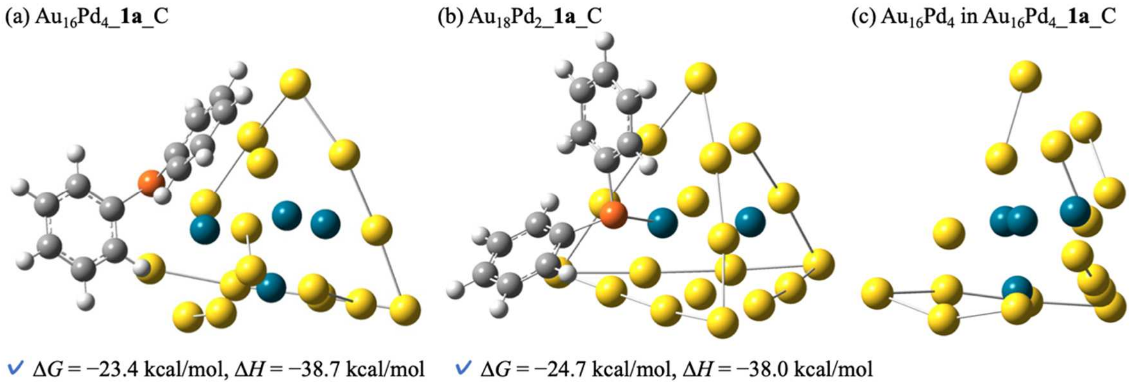

Fig. S10 Summary of adsorption energies and optimized structures of (a) $Au_{16}Pd_4$_**1a**_C, (b) $Au_{18}Pd_2$_**1a**_C, and (c) $Au_{18}Pd_2$ in $Au_{18}Pd_2$_**1a**_C.

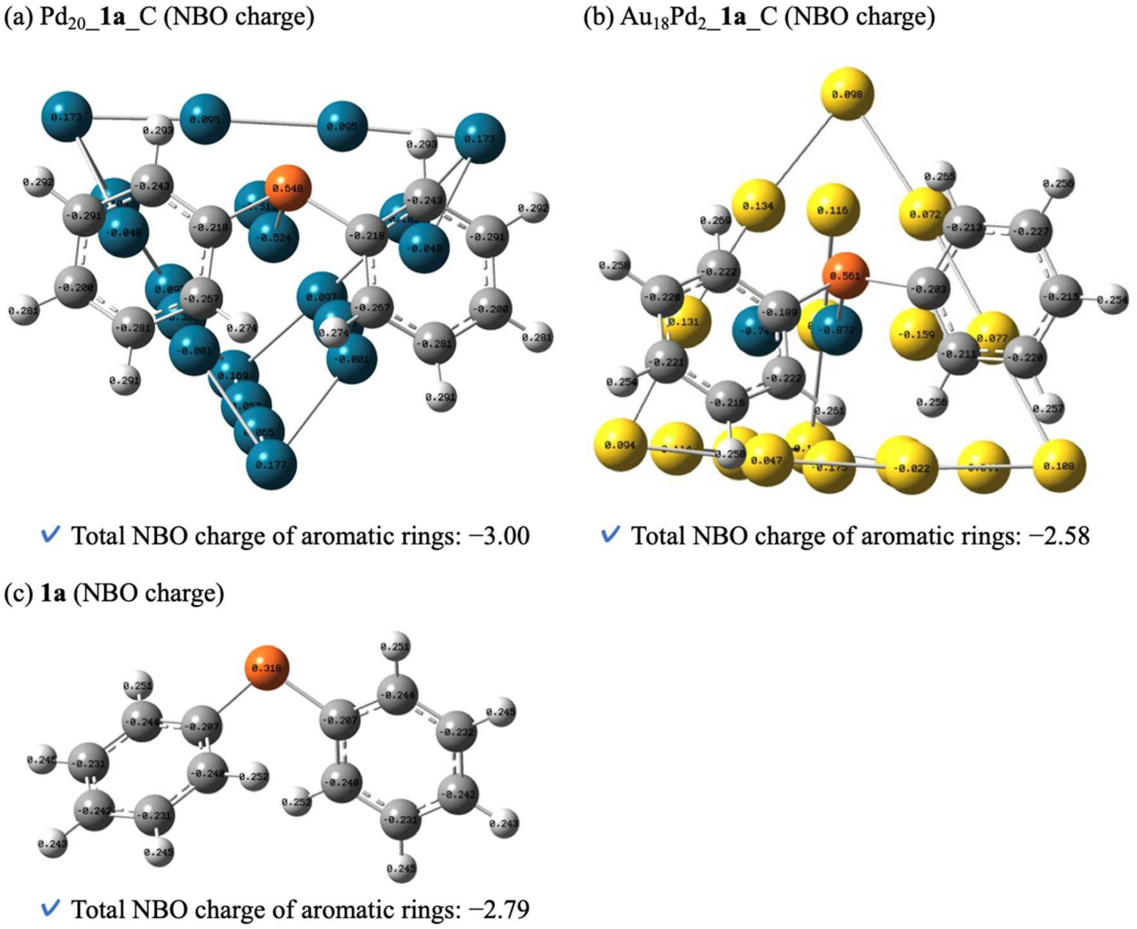

Fig. S11 NBO charge in (a) Pd_{20}_**1a**_C, (b) $Au_{18}Pd_2$_**1a**_C, and (c) free **1a**.

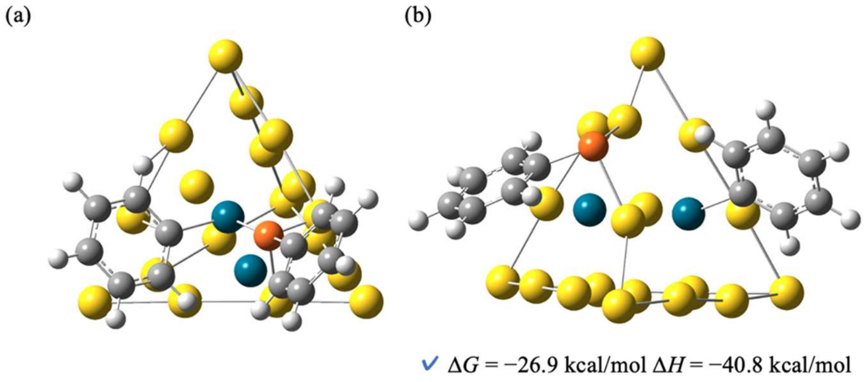

Fig. S12 (a) An initial structure of the **1a** oxidative adduct only on the Pd atom of $Au_{18}Pd_2$ and (b) the optimized structure after the DFT calculation using the initial structure with the adsorption energy.

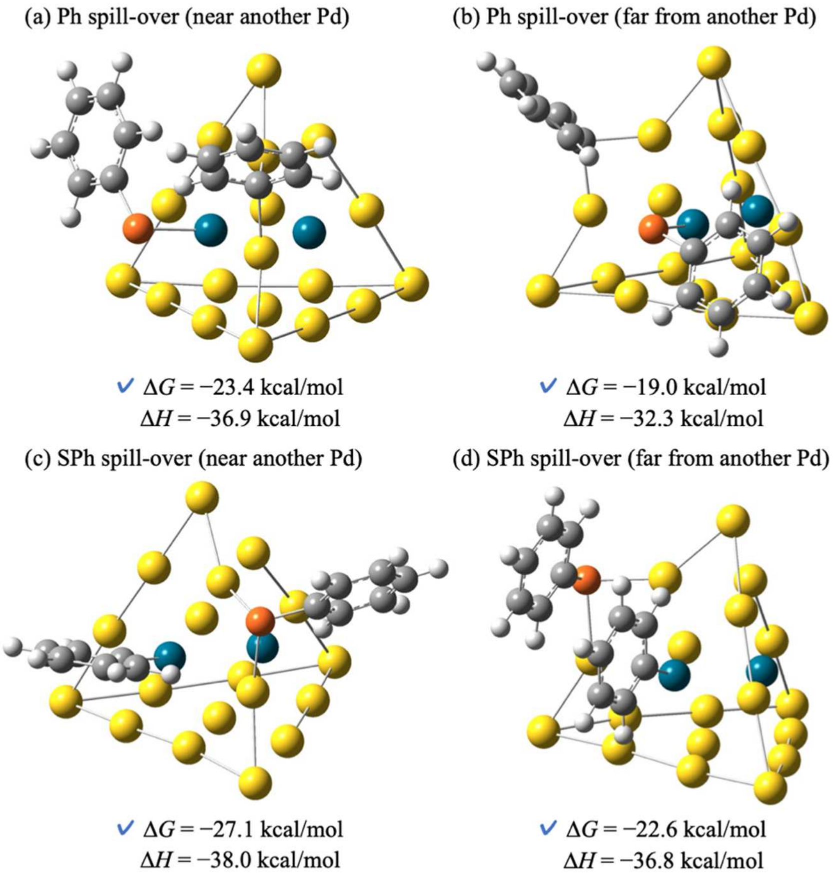

Fig. S13 Summary and adsorption energies of optimized structures of **1a** oxidative adducts on $Au_{18}Pd_2$.

(a)

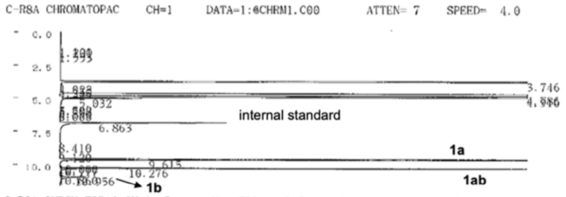

C-R8A CHROMATOPAC CH=1 Report No.=533 DATA=1:@CHRM1.C00 22/12/16 20:32:24

** CALCULATION REPORT **

CH	PKNO	TIME	AREA	HEIGHT	MK	IDNO	CONC	NAME
1	4	3.746	11561026	5764271	S		35.4517	
	8	4.786	14894512	3608831	V		45.6738	
	9	4.916	4896794	1964451	SV		15.0159	
	10	5.032	34473	21204	T		0.1057	
	15	6.863	126299	45592			0.3873	internal standard
	18	9.615	752294	286536	S		2.3069	**1a**
	22	10.276	311171	143029	V		0.9542	**1ab**
	24	10.956	34079	7094	V		0.1045	**1b**

(b)

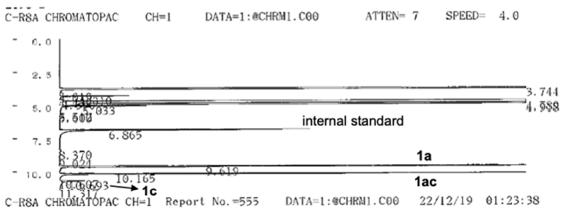

C-R8A CHROMATOPAC CH=1 Report No.=555 DATA=1:@CHRM1.C00 22/12/19 01:23:38

** CALCULATION REPORT **

CH	PKNO	TIME	AREA	HEIGHT	MK	IDNO	CONC	NAME
1	1	3.744	12538800	5920016	S		36.4358	
	3	4.31	43810	19013	T		0.1273	
	6	4.789	15327731	3639278	V		44.5401	
	7	4.918	5082576	2105554	SV		14.7692	
	8	5.033	37434	22699	T		0.1088	
	11	6.865	166219	68684			0.483	internal standard
	14	9.619	828072	314963			2.4063	**1a**
	15	10.165	355301	157324	SV		1.0324	**1ac**
	17	10.693	33421	6799	T		0.0971	**1c**

(c)

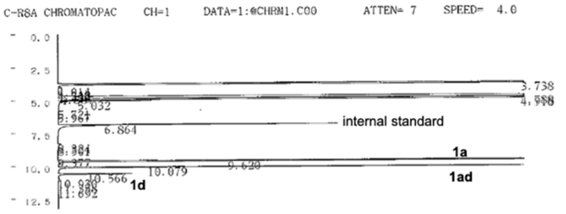

C-R8A CHROMATOPAC CH=1 Report No.=554 DATA=1:@CHRM1.C00 22/12/19 01:06:00

** CALCULATION REPORT **

CH	PKNO	TIME	AREA	HEIGHT	MK	IDNO	CONC	NAME
1	1	3.738	13969779	6400407	S		38.1086	
	7	4.788	15988555	3713256	V		43.6157	
	8	4.918	5252868	2117943	SV		14.3295	
	9	5.032	38611	24564	T		0.1053	
	12	6.864	175056	76880			0.4775	internal standard
	13	8.324	4490	756			0.0122	
	16	9.62	846293	323870			2.3086	**1a**
	17	10.079	328817	159953	V		0.897	**1ad**
	18	10.566	53359	20342	SV		0.1456	**1d**

(d)

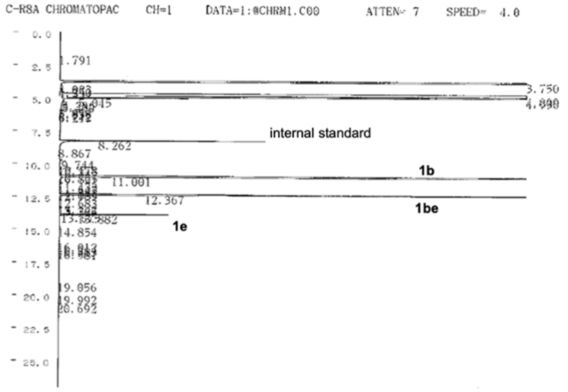

C-R8A CHROMATOPAC CH=1 Report No.=820 DATA=1:@CHRM1.C00 23/02/13 20:02:00

** CALCULATION REPORT **

CH	PKNO	TIME	AREA	HEIGHT	MK	IDNO	CONC	NAME
1	2	3.75	12269550	5468488	S		29.0827	
	5	4.334	1177	440	T		0.0028	
	6	4.809	20989778	4221428	V		49.7524	
	7	4.936	6821214	2601410	V		16.1684	
	8	5.045	64064	35260	V		0.1519	
	9	5.286	8980	1066	V		0.0213	
	10	5.45	6646	438	V		0.0158	
	12	5.933	1215	160	V		0.0029	
	15	8.262	121853	56421	S		0.2888	internal standard
	17	9.744	3271	703			0.0078	
	20	10.761	1079	497			0.0026	
	22	11.001	1236750	353946	SV		2.9315	**1b**
	28	12.367	584187	197510			1.3847	**1be**
	29	12.683	1060	362	V		0.0025	
	34	13.882	77703	29969			0.1842	**1e**

Fig. S14 GC charts of the reaction solution after $Au_{4.4}$–Pd_1/TiO_2-catalyzed thioether metathesis between (a) **1a** and **1b**, (b) **1a** and **1c**, (c) **1a** and **1d**, and (d) **1b** and **1e**. The reaction conditions are shown in Scheme 2.

(e)

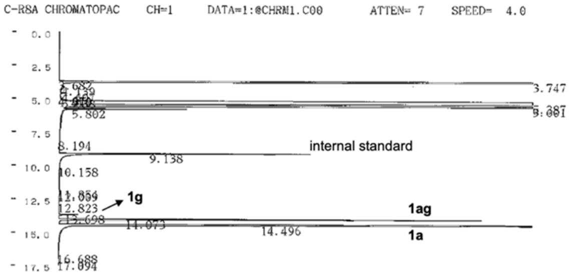

C-R8A CHROMATOPAC CH=1 Report No.=4 DATA=1:@CHRM1.C00 24/05/27 19:36:52

** CALCULATION REPORT **

CH	PKNO	TIME	AREA	HEIGHT	MK	IDNO	CONC	NAME
1	1	3.682	2062	1231			0.0049	
	2	3.747	16872482	7345162	SV		40.0991	
	4	4.607	4953	1592	T		0.0118	
	8	5.387	17420452	2936001	V		41.4014	
	9	5.601	6061988	1732020	SV		14.4069	
	10	5.802	60057	33153	T		0.1427	
	12	9.138	215548	67897	S		0.5123	internal standard
	15	12.009	5633	352	V		0.0134	
	17	13.698	15914	5209			0.0378	**1g**
	18	14.073	349040	114359			0.8295	**1ag**
	19	14.496	1066833	249631	V		2.5354	**1a**
	21	17.094	2049	126			0.0049	

(f)

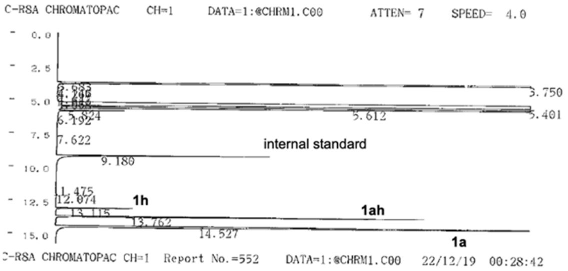

C-R8A CHROMATOPAC CH=1 Report No.=552 DATA=1:@CHRM1.C00 22/12/19 00:28:42

** CALCULATION REPORT **

CH	PKNO	TIME	AREA	HEIGHT	MK	IDNO	CONC	NAME
1	2	3.75	13603000	6191031	SV		40.4277	
	8	5.401	14061443	2585021	V		41.7901	
	9	5.612	4697720	1452652	SV		13.9615	
	10	5.824	33808	17590	T		0.1005	
	13	9.18	168804	57688			0.5017	internal standard
	16	13.115	50772	20847			0.1509	**1h**
	17	13.762	254646	99578			0.7568	**1ah**
	18	14.527	777559	209059			2.3109	**1a**

(g)

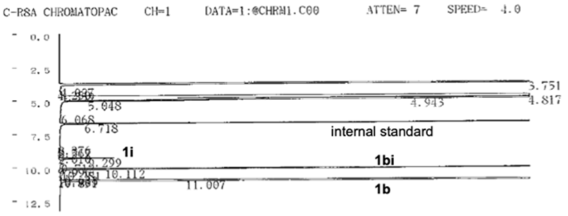

C-R8A CHROMATOPAC CH=1 Report No.=725 DATA=1:@CHRM1.C00 23/02/02 20:23:46

** CALCULATION REPORT **

CH	PKNO	TIME	AREA	HEIGHT	MK	IDNO	CONC	NAME
1	1	3.751	11382245	4967585	S		24.7758	
	4	4.339	1406	487	T		0.0031	
	5	4.817	24313580	4387002	V		52.9235	
	6	4.943	7878320	2851154	V		17.1488	
	7	5.048	85211	40759	SV		0.1855	
	9	6.718	322499	165709			0.702	internal standard
	13	9.299	46399	14830	S		0.101	**1i**
	14	9.712	3795	1018	T		0.0083	
	16	10.112	492204	210903	SV		1.0714	**1bi**
	19	10.769	1026	456	T		0.0022	
	20	10.831	1080	490	TV		0.0023	
	21	11.007	1413281	382743	V		3.0763	**1b**

(h)

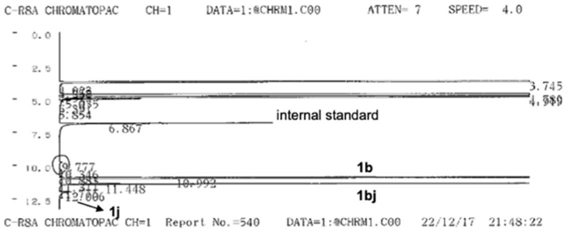

C-R8A CHROMATOPAC CH=1 Report No.=540 DATA=1:@CHRM1.C00 22/12/17 21:48:22

** CALCULATION REPORT **

CH	PKNO	TIME	AREA	HEIGHT	MK	IDNO	CONC	NAME
1	1	3.745	11047504	5824337	S		34.2081	
	6	4.789	14875900	3623365	V		46.0626	
	7	4.919	4904591	2065557	V		15.1869	
	8	5.035	49812	21908	SV		0.1542	
	11	6.867	154404	58099			0.4781	internal standard
	12	9.777	3948	788			0.0122	
	16	10.992	923001	299719	V		2.858	**1b**
	18	11.448	314548	132075	SV		0.974	**1bj**
	20	12.006	21248	3334	T		0.0658	**1j**

Fig. S14 (continued) GC charts of the reaction solution after $Au_{4.4}$–Pd_1/TiO_2-catalyzed thioether metathesis between (e) **1a** and **1g**, (f) **1a** and **1h**, (g) **1b** and **1i**, and (h) **1b** and **1j**. The reaction conditions are shown in Scheme 2.

(i)

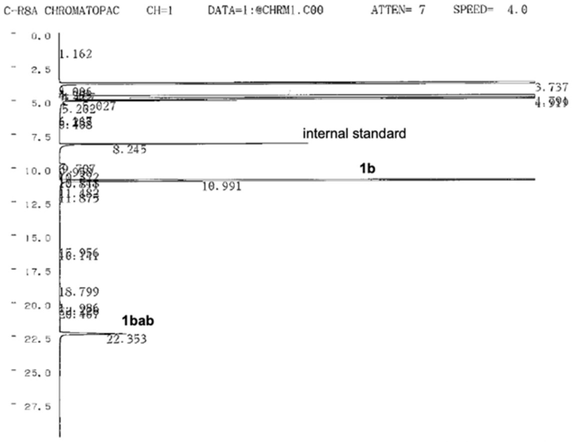

C-R8A CHROMATOPAC CH=1 Report No.=839 DATA=1:@CHRM1.C00 24/01/19 03:27:02

** CALCULATION REPORT **

CH	PKNO	TIME	AREA	HEIGHT	MK	IDNO	CONC	NAME
1	2	3.737	18187096	6490680	S		37.7758	
	5	4.321	2830	823	T		0.0059	
	6	4.407	2569	758	TV		0.0053	
	7	4.791	21006210	4109252	V		43.6312	
	8	4.919	7102985	2671030	V		14.7533	
	9	5.027	84854	47810	V		0.1762	
	10	5.262	18441	1590	V		0.0383	
	14	8.245	155888	66942			0.3238	internal standard
	15	9.707	7239	1556	S		0.015	
	20	10.991	1445285	382897	SV		3.0019	**1b**
	29	22.353	131504	18011			0.2731	**1bab**

Fig. S14 (continued) GC charts of the reaction solution after Au$_{4.4}$–Pd$_1$/TiO$_2$-catalyzed thioether metathesis between (i) **1a′** and **1b**. The reaction conditions are shown in Scheme 2.

Supplementary Tables

Table S1 Effect of Pd catalysts on the metathesis between **1a** and **1b**.[a]

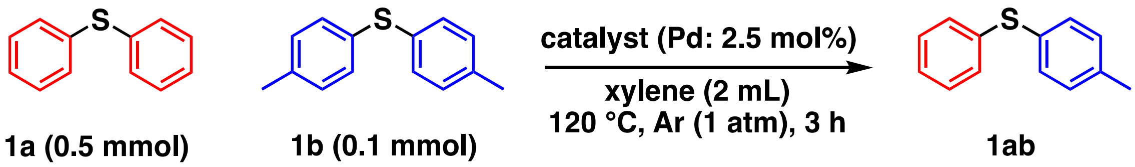

entry	catalyst	conv. (%)		yield (%)
		**1a**	**1b**	**1ab**
1	Pd_1/TiO_2	9	11	1
2	Pd_3/TiO_2	13	16	2
3	Pd_5/TiO_2	12	13	2
4[b]	Pd_1/TiO_2 + PCy_3	<1	3	<1

[a]Reaction conditions: **1a** (0.5 mmol), **1b** (0.1 mmol), catalyst (Pd: 2.5 mol%), xylene (2 mL), 120 °C, 3 h in test tube. Conversions and yields were determined by GC analysis using 1,3,5-trimethoxybenzene as an internal standard. [b]PCy_3 (2.5 mol%). PCy_3 = tricyclohexylphosphine.

Table S2 Fitted parameters from (a) Pd K-edge and (b) Au L_{III}-edge EXAFS spectra of $Au_{4.4}$–Pd_1/TiO_2.

(a)

Shell	C.N.	*R* (Å)	ΔE_{j0} (eV)	$\sigma^2 \times 10^2$ (Å^2)	*R*-factor (%)
Pd–O	0.9±0.2	2.04	10 (fixed)	0.577	0.38
Pd–Pd (in Pd)	0.7±0.2	2.82	-10 (fixed)	0.506	
Pd–Au	5.0±0.6	2.83	-1.72 (fixed)	0.773	
Pd–Pd (in Au–Pd)	1.1±1.0	3.94	-8.62 (fixed)	0.634	

(b)

Shell	C.N.	*R* (Å)	ΔE_{j0} (eV)	$\sigma^2 \times 10^2$ (Å^2)	*R*-factor (%)
Au–Pd	1.1±0.3	2.80	3.51 (fixed)	0.751	0.17
Au–Au	8.2±0.5	2.82	4.39 (fixed)	0.930	

Table S3 Effect of the second metals with Pd on the metathesis between **1a** and **1b**.[a]

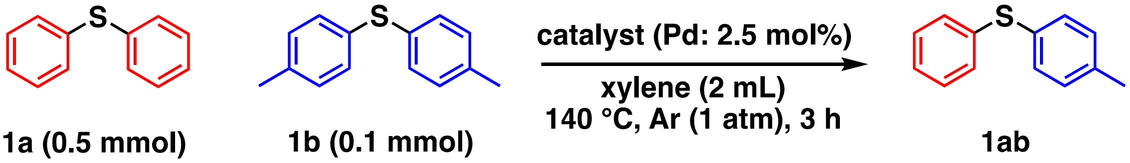

entry	catalyst	conv. (%)		yield (%)
		**1a**	**1b**	**1ab**
1	$Au_{4.4}$–Pd_1/TiO_2	23	80	69
2	Ru_6–Pd_1/TiO_2	6	30	28
3	Co_6–Pd_1/TiO_2	4	20	19
4	Ni_6–Pd_1/TiO_2	3	5	1
5	Ag_6–Pd_1/ZrO_2	8	8	2

[a]Reaction conditions: **1a** (0.5 mmol), **1b** (0.1 mmol), catalyst (Pd: 2.5 mol%), xylene (2 mL), 140 °C, 3 h in test tube. Conversions and yields were determined by GC analysis using 1,3,5-trimethoxybenzene as an internal standard.

Table S4 Effect of the supports on the metathesis between **1a** and **1b**.[a]

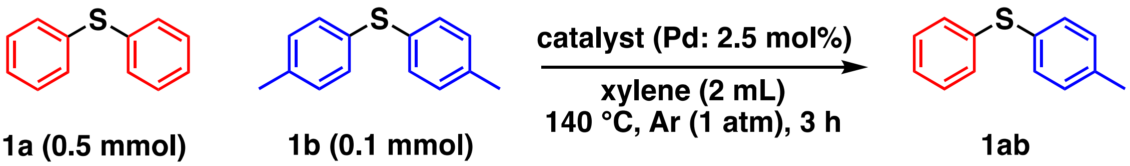

entry	catalyst	conv. (%)		yield (%)
		**1a**	**1b**	**1ab**
1	$Au_{4.4}$–Pd_1/TiO_2	23	80	69
2	$Au_{1.4}$–Pd_1/CeO_2	24	70	57
3	$Au_{5.4}$–Pd_1/LDH	18	51	39
4	$Au_{3.6}$–Pd_1/Al_2O_3	13	34	24
5	$Au_{2.6}$–Pd_1/HAP	14	33	21
6	$Au_{2.5}$–Pd_1/ZrO_2	12	19	8

[a]Reaction conditions: **1a** (0.5 mmol), **1b** (0.1 mmol), catalyst (Pd: 2.5 mol%), xylene (2 mL), 140 °C, 3 h in test tube. Conversions and yields were determined by GC analysis using 1,3,5-trimethoxybenzene as an internal standard.

Table S5 Effect of the solvents on the metathesis between **1a** and **1b** [a]

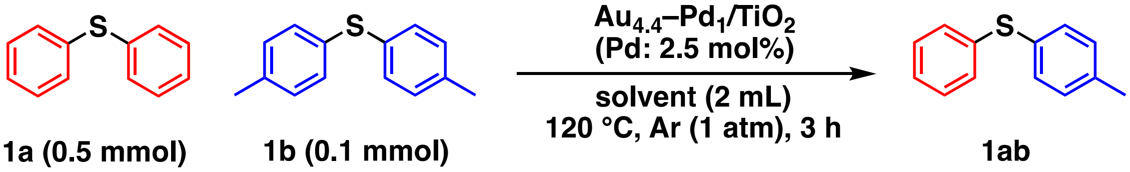

entry	solvent	conv. (%)		yield (%)
		**1a**	**1b**	**1ab**
1	xylene	18	66	55
2	1,4-dioxane	12	45	44
3	heptane	12	41	34
4	NMP	15	37	33
5	MCH	10	34	22
6	DMA	10	24	17

[a]Reaction conditions: **1a** (0.5 mmol), **1b** (0.1 mmol), $Au_{4.4}$–Pd_1/TiO_2 (Pd: 2.5 mol%), solvent (2 mL), 140 °C, 3 h in test tube. Conversions and yields were determined by GC analysis using 1,3,5-trimethoxybenzene as an internal standard. NMP = *N*-methylpyrrolidone, MCH = methylcyclohexane, DMA = *N*,*N*-dimethylacetamide.

Table S6 Effect of the reaction temperatures on the metathesis between **1a** and **1b** [a]

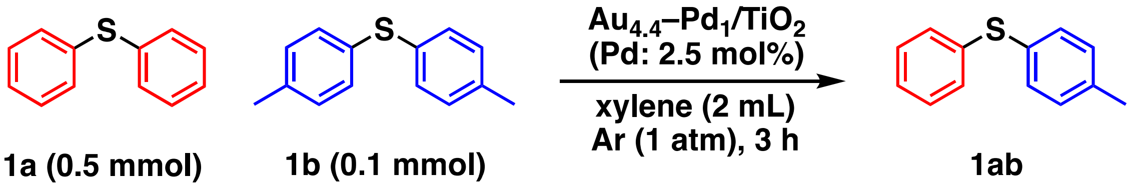

entry	Temperature (°C)	conv. (%)		yield (%)
		**1a**	**1b**	**1ab**
1	140	23	80	69
2	120	18	66	55
3	100	12	25	17
4[b]	140	21	84	77
5[b]	120	22	80	70
6[b]	100	19	62	51

[a]Reaction conditions: **1a** (0.5 mmol), **1b** (0.1 mmol), $Au_{4.4}$–Pd_1/TiO_2 (Pd: 2.5 mol%), xylene (2 mL), 3 h in test tube. Conversions and yields were determined by GC analysis using 1,3,5-trimethoxybenzene as an internal standard. [b]24 h.

Table S7 Effect of **1a** amounts on the metathesis between **1a** and **1b**[a]

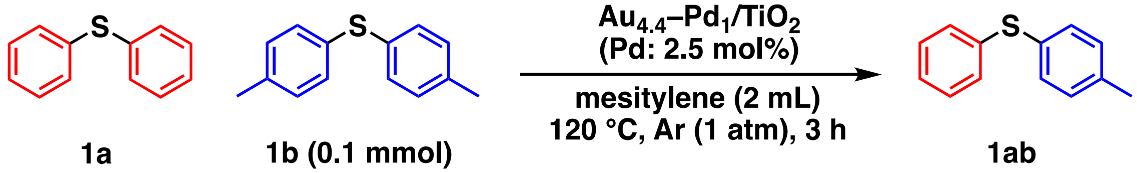

entry	**1a** amount (mmol)	conv. (%)		yield (%)
		**1a**	**1b**	**1ab**
1	0.1	52	54	46
2	0.3	28	69	60
3	0.5	18	66	55
4	0.7	8	28	21
5[b]	0.1	52	58	48
6[b]	0.3	26	74	68
7[b]	0.5	21	84	77
8[b]	0.7	11	56	49

[a]Reaction conditions: **1a** (0.5 mmol), **1b** (0.1 mmol), $Au_{4.4}$–Pd_1/TiO_2 (Pd: 2.5 mol%), xylene (2 mL), 120 °C, 3 h in test tube. Conversions and yields were determined by GC analysis using 1,3,5-trimethoxybenzene as an internal standard. [b]140 °C, 24 h.

NMR Spectra

^{1}H NMR spectrum (500 MHz, $CDCl_3$) of **1af**

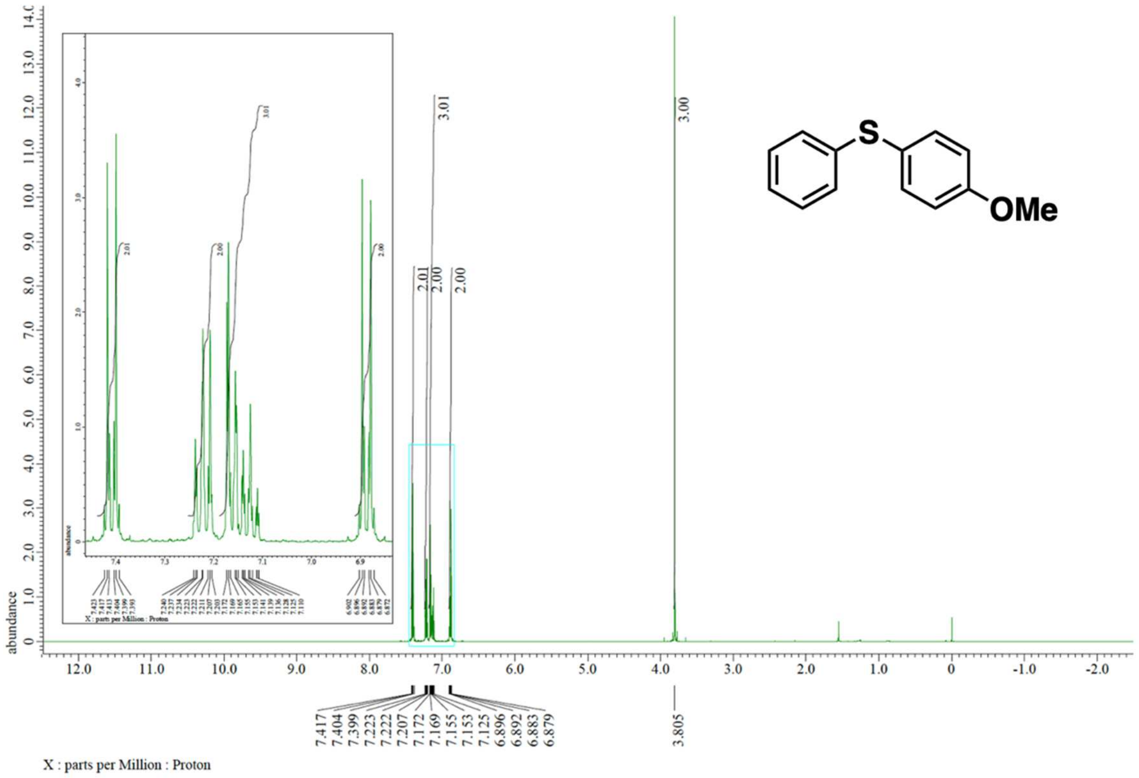

^{13}C NMR spectrum (125 MHz, $CDCl_3$) of **1af**

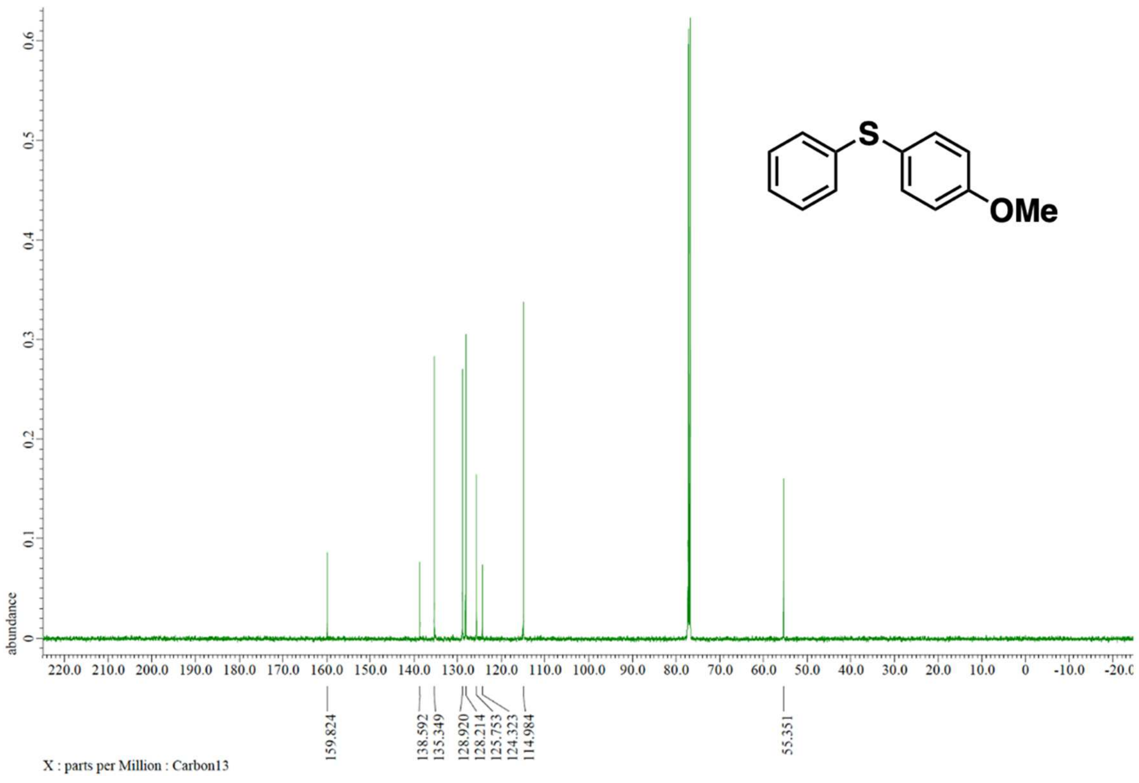

^{1}H NMR spectrum (500 MHz, $CDCl_3$) of **1bk**

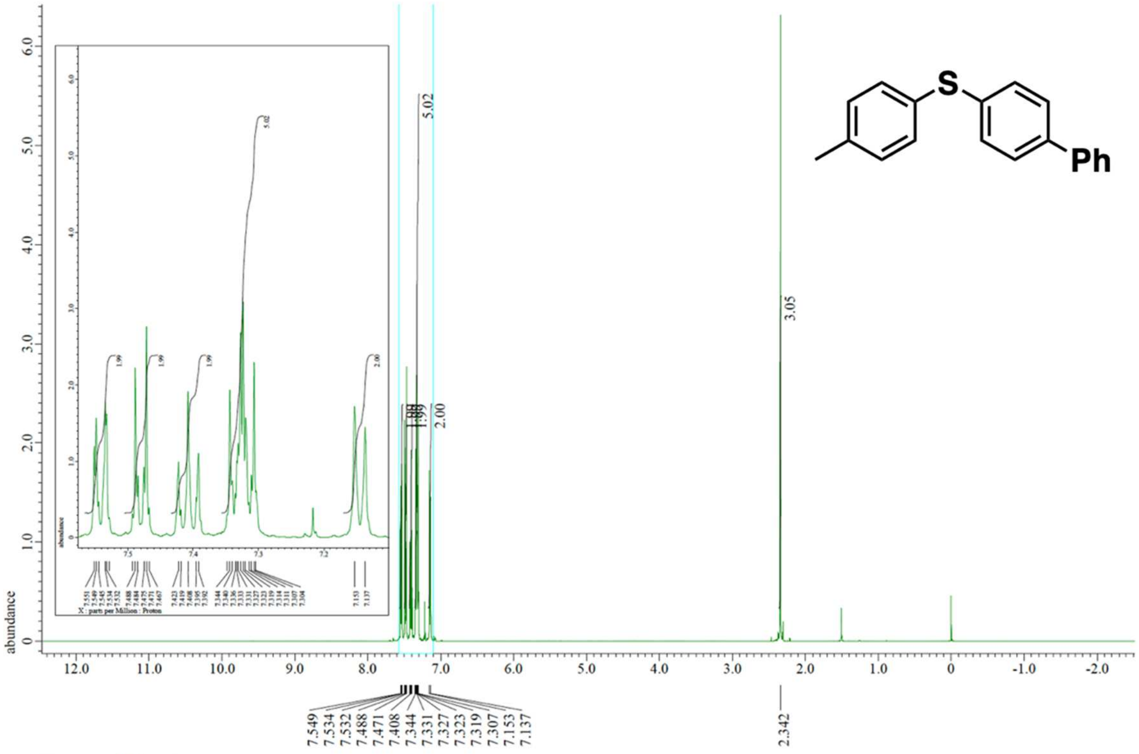

^{13}C NMR spectrum (125 MHz, $CDCl_3$) of **1bk**

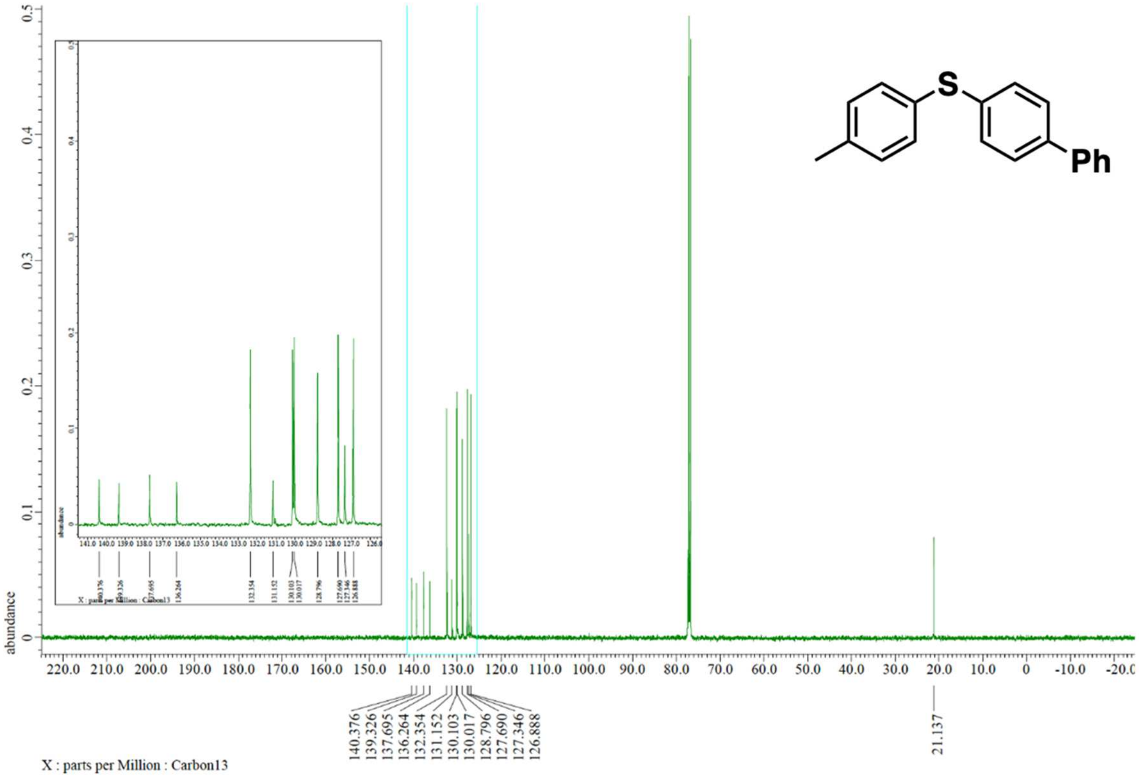

^{1}H NMR spectrum (500 MHz, $CDCl_3$) of **1bl**

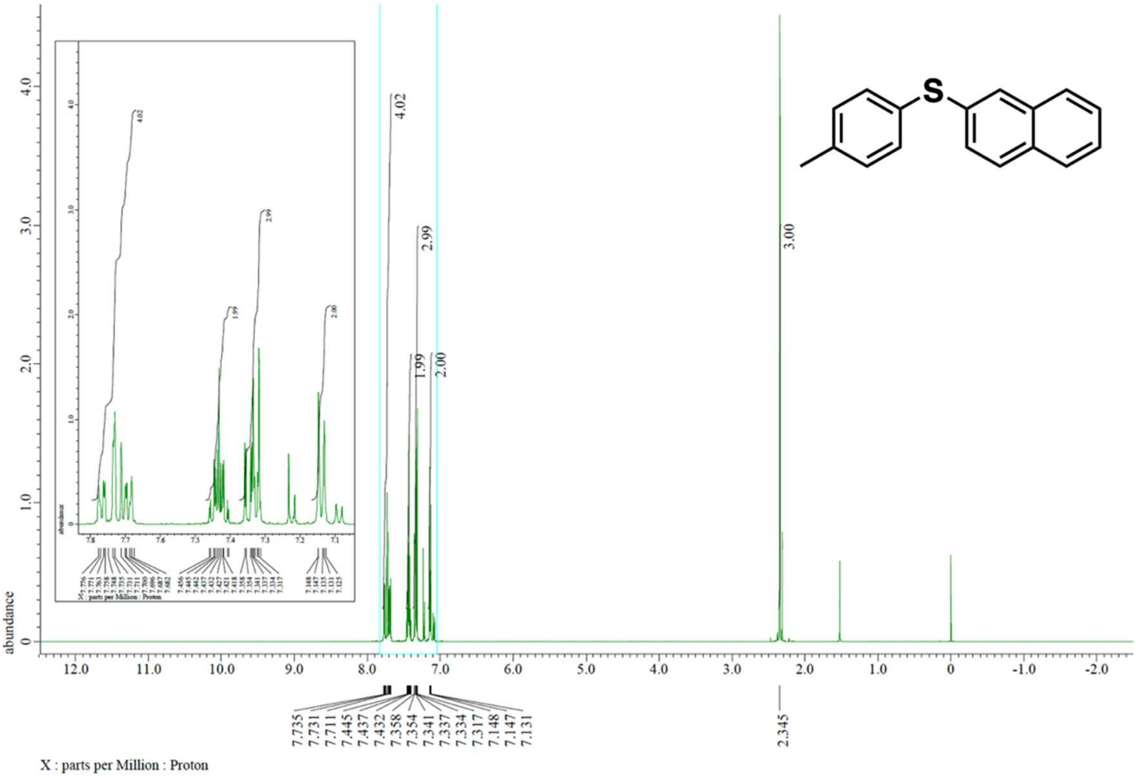

^{13}C NMR spectrum (125 MHz, $CDCl_3$) of **1bl**

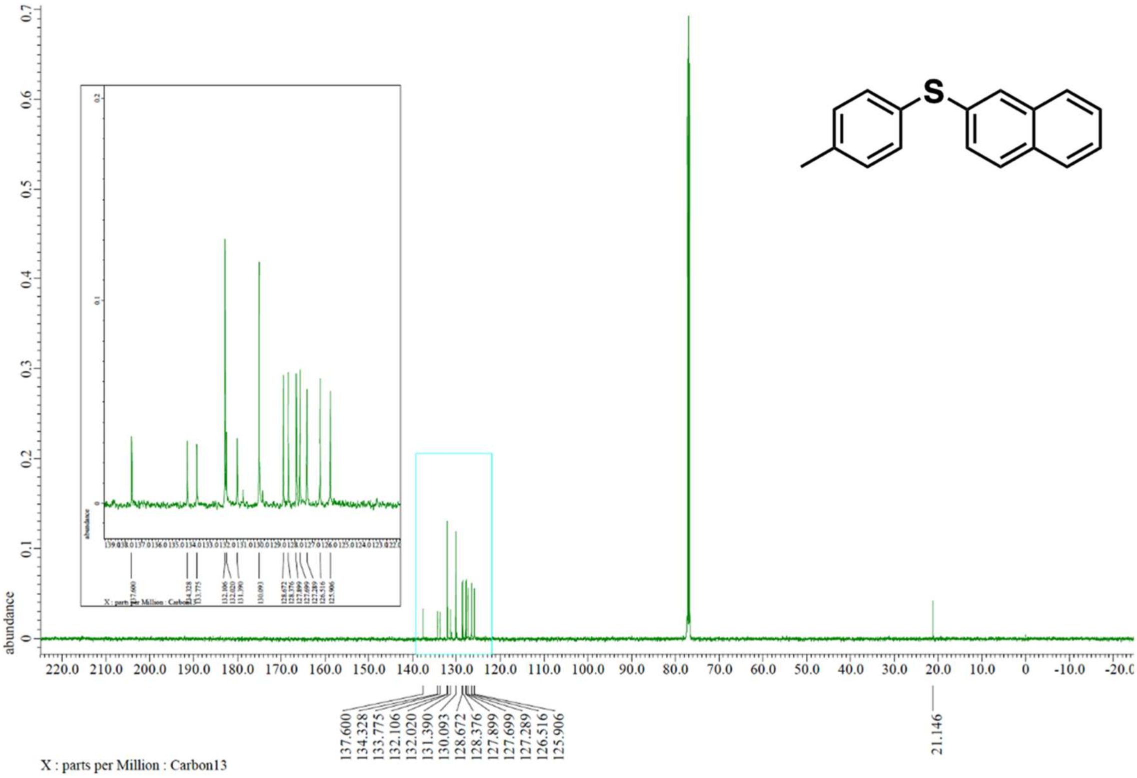

^{1}H NMR spectrum (500 MHz, $CDCl_3$) of **1am**

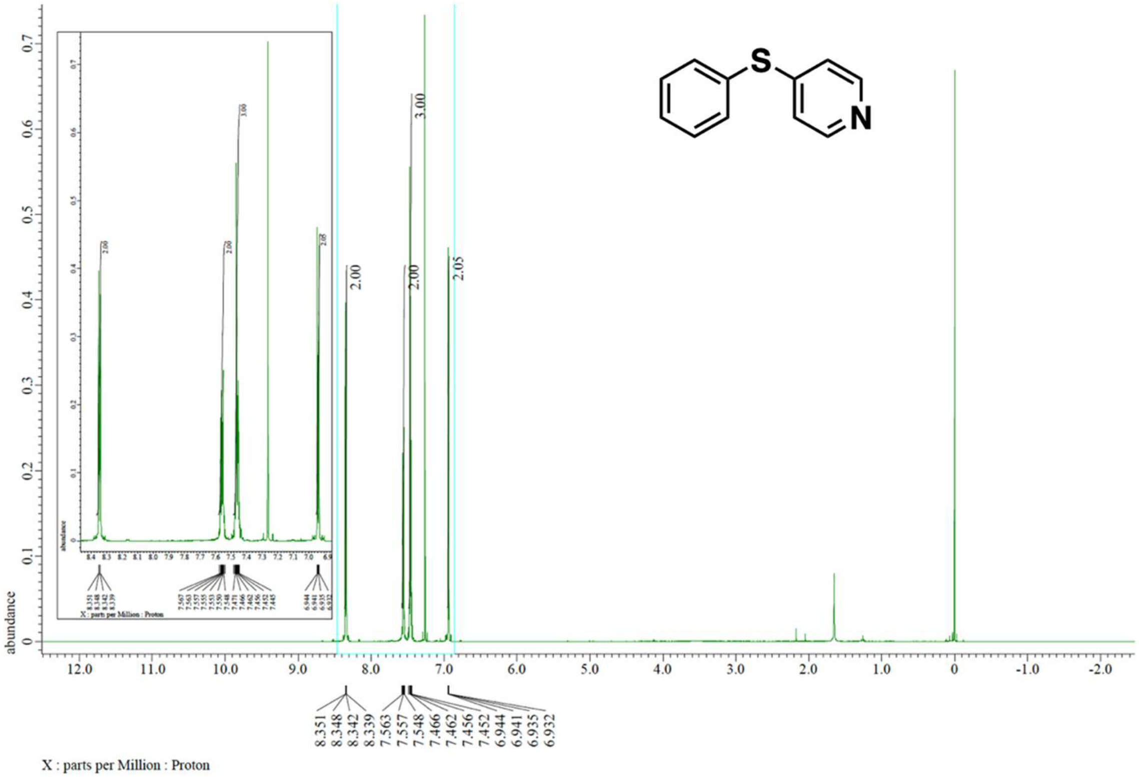

^{13}C NMR spectrum (125 MHz, $CDCl_3$) of **1am**

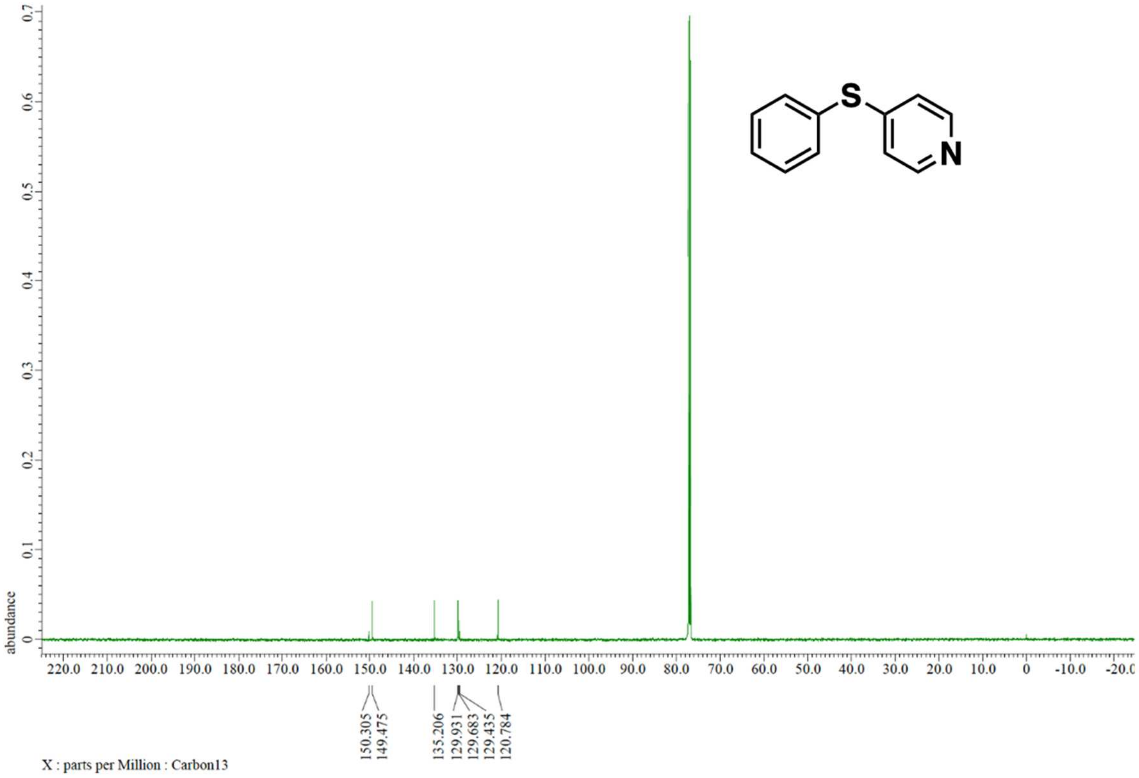

^{1}H NMR spectrum (500 MHz, $CDCl_3$) of **1an**

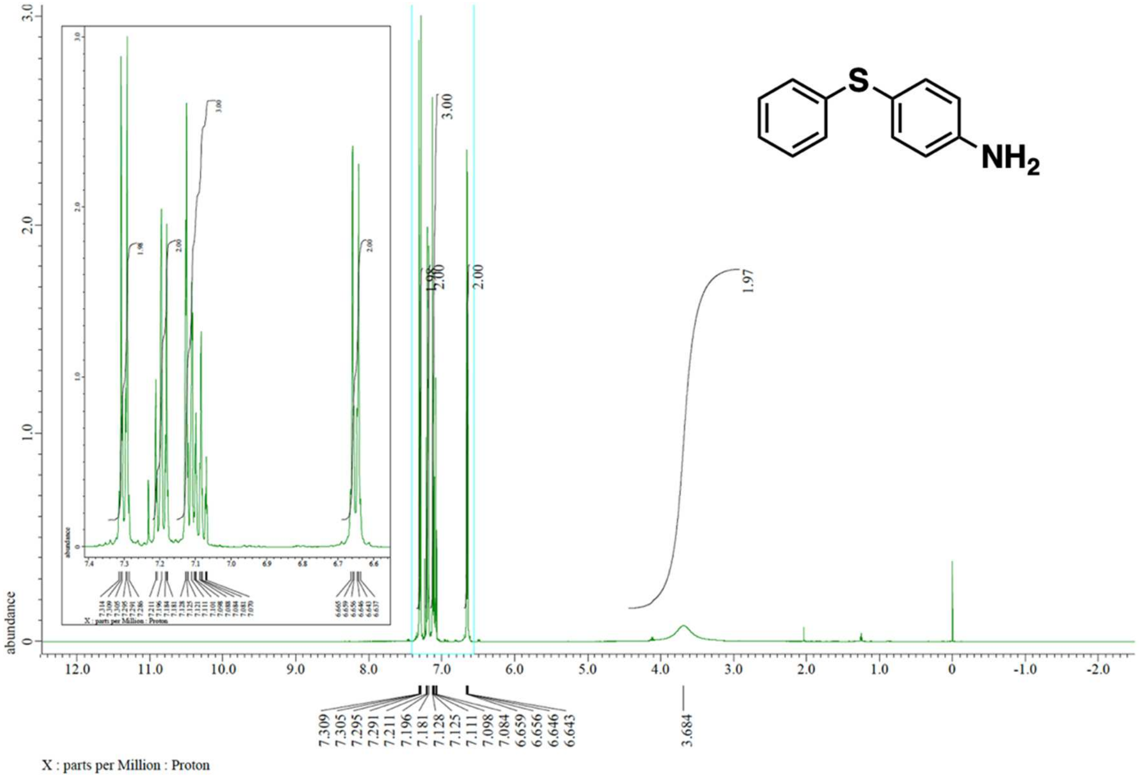

^{13}C NMR spectrum (125 MHz, $CDCl_3$) of **1an**

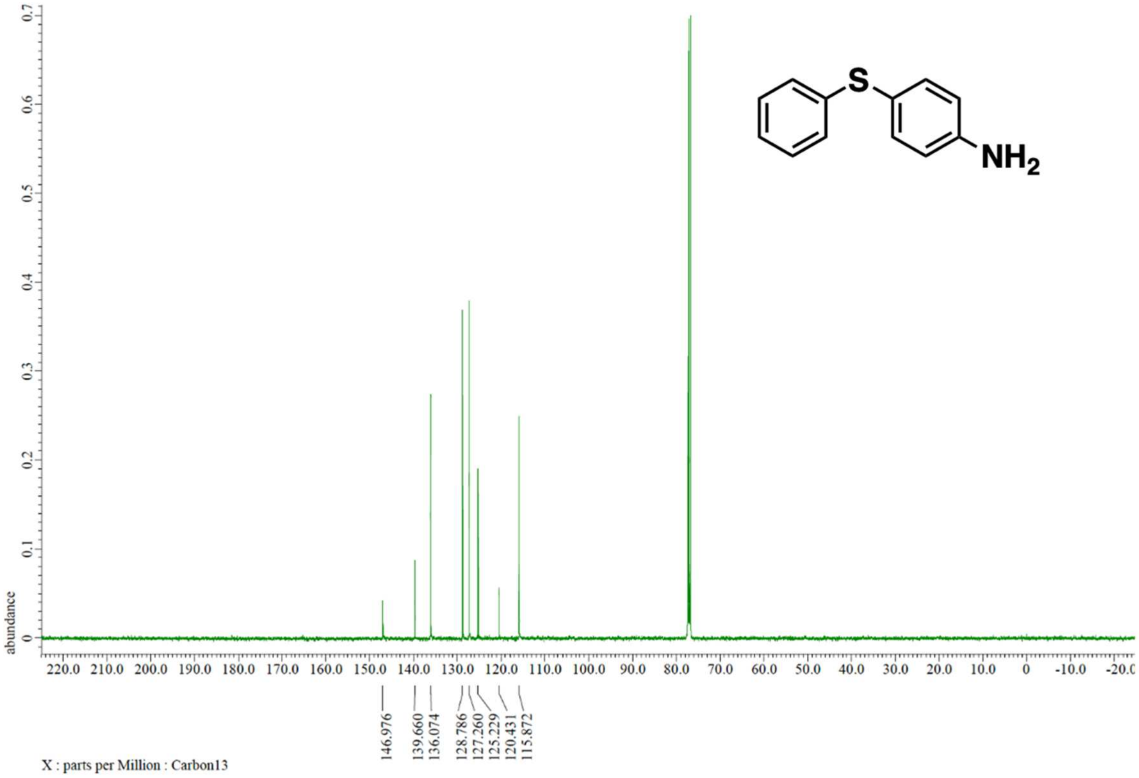

^{1}H NMR spectrum (500 MHz, $CDCl_3$) of **1bo**

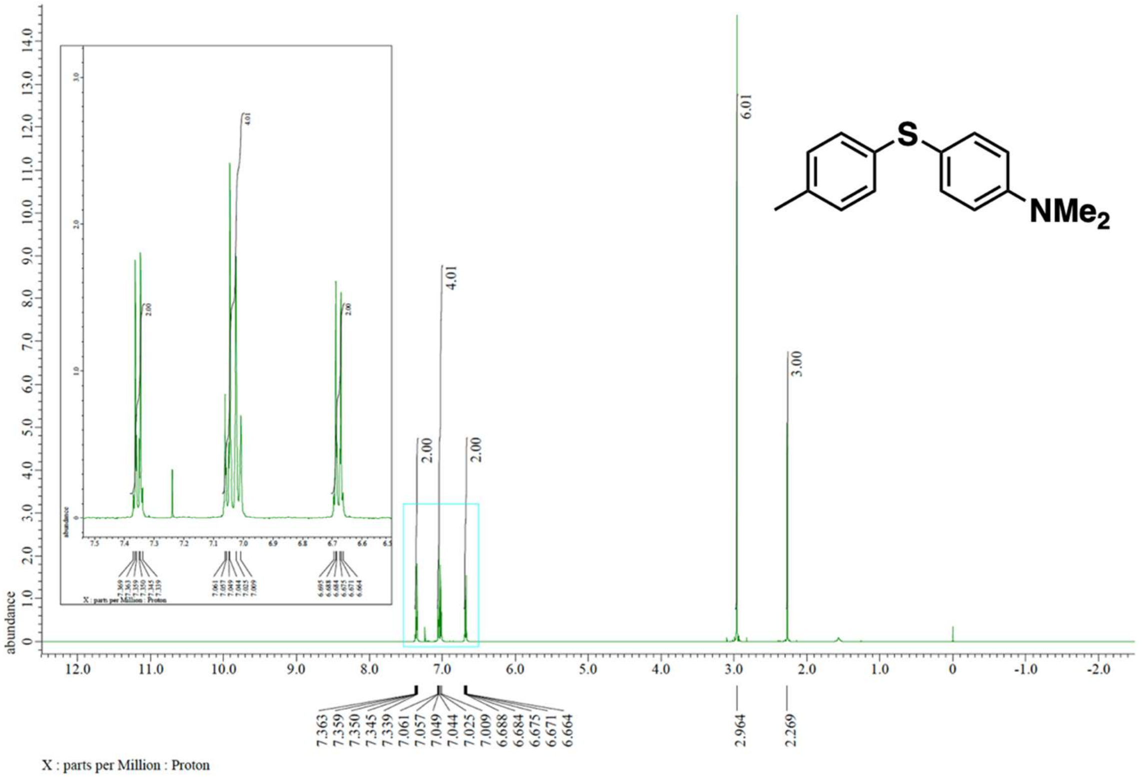

^{13}C NMR spectrum (125 MHz, $CDCl_3$) of **1bo**

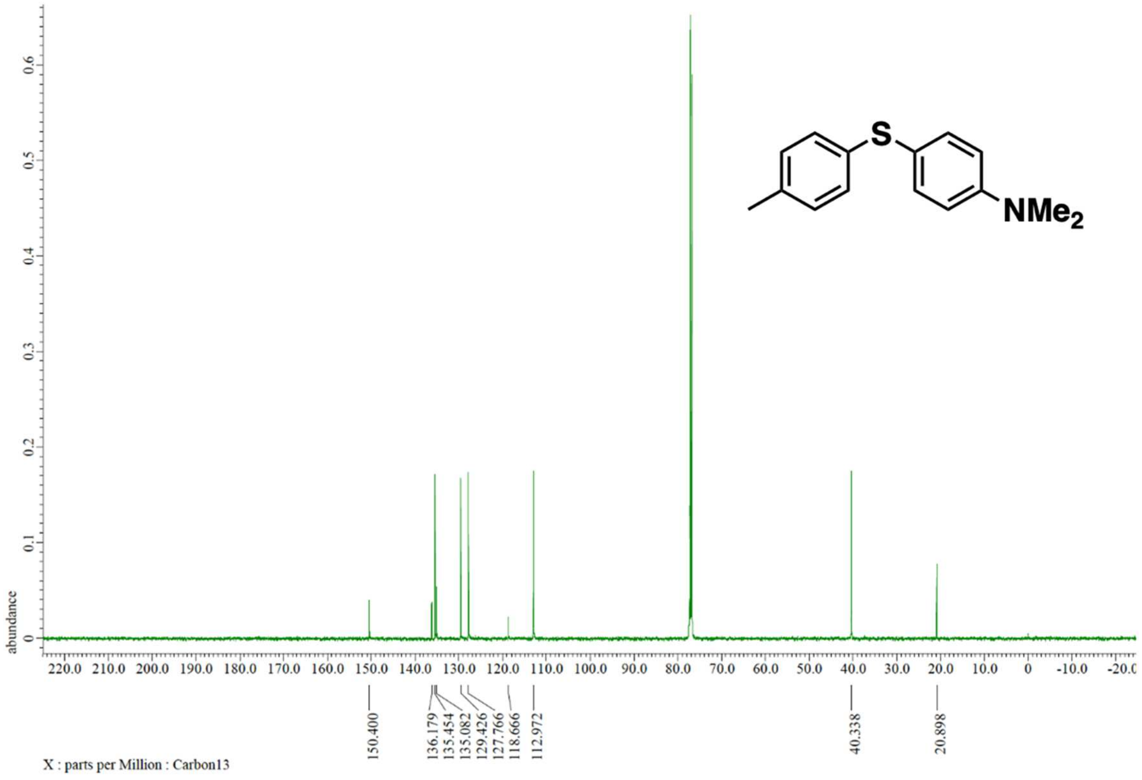

^{1}H NMR spectrum (500 MHz, $CDCl_3$) of **1bp**

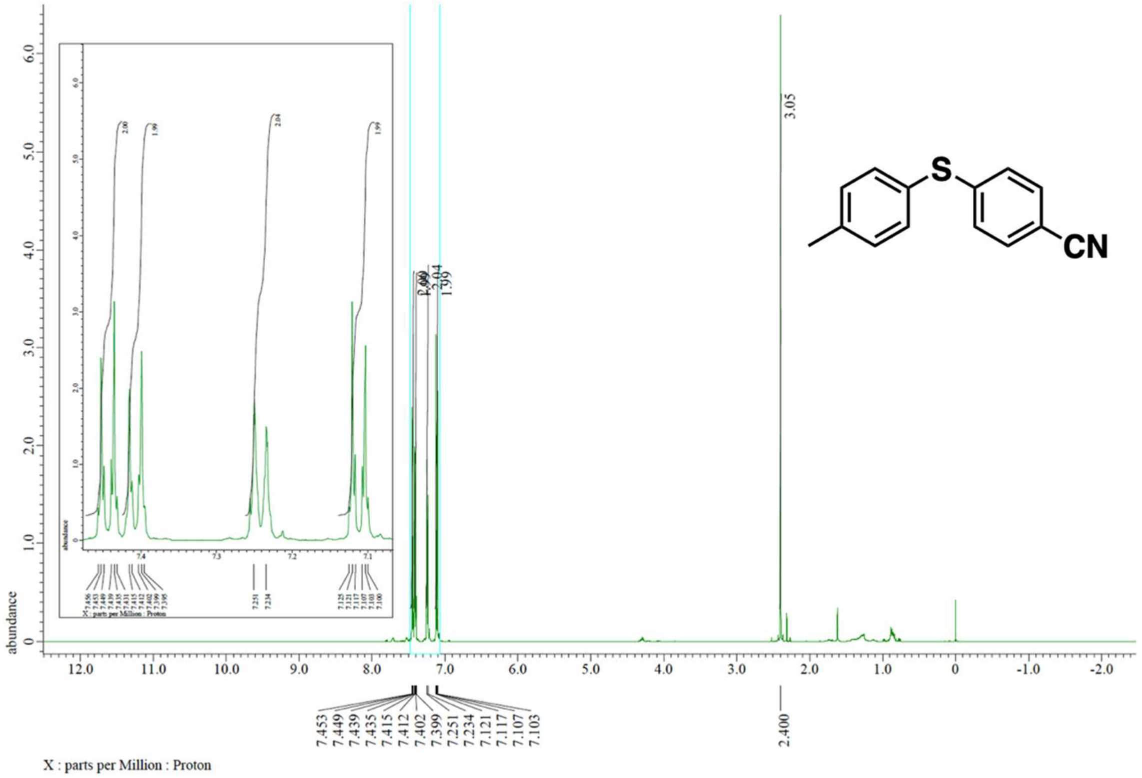

^{13}C NMR spectrum (125 MHz, $CDCl_3$) of **1bp**

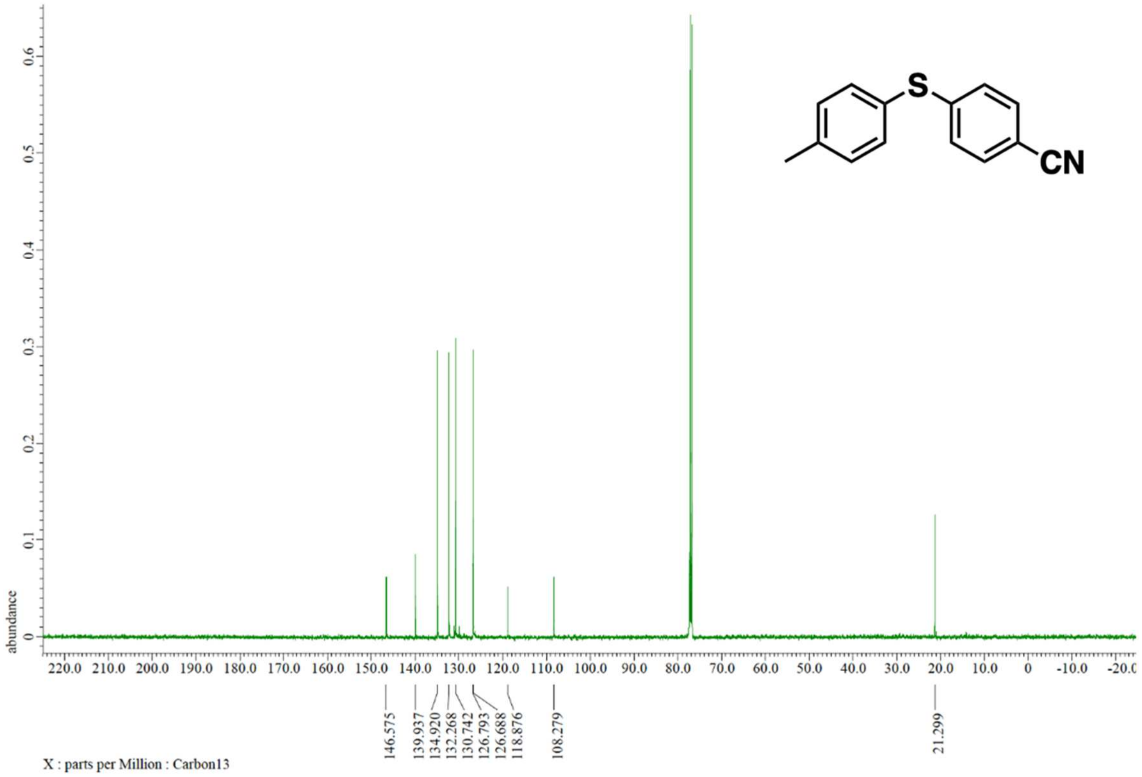

^{1}H NMR spectrum (500 MHz, $CDCl_3$) of **1aq**

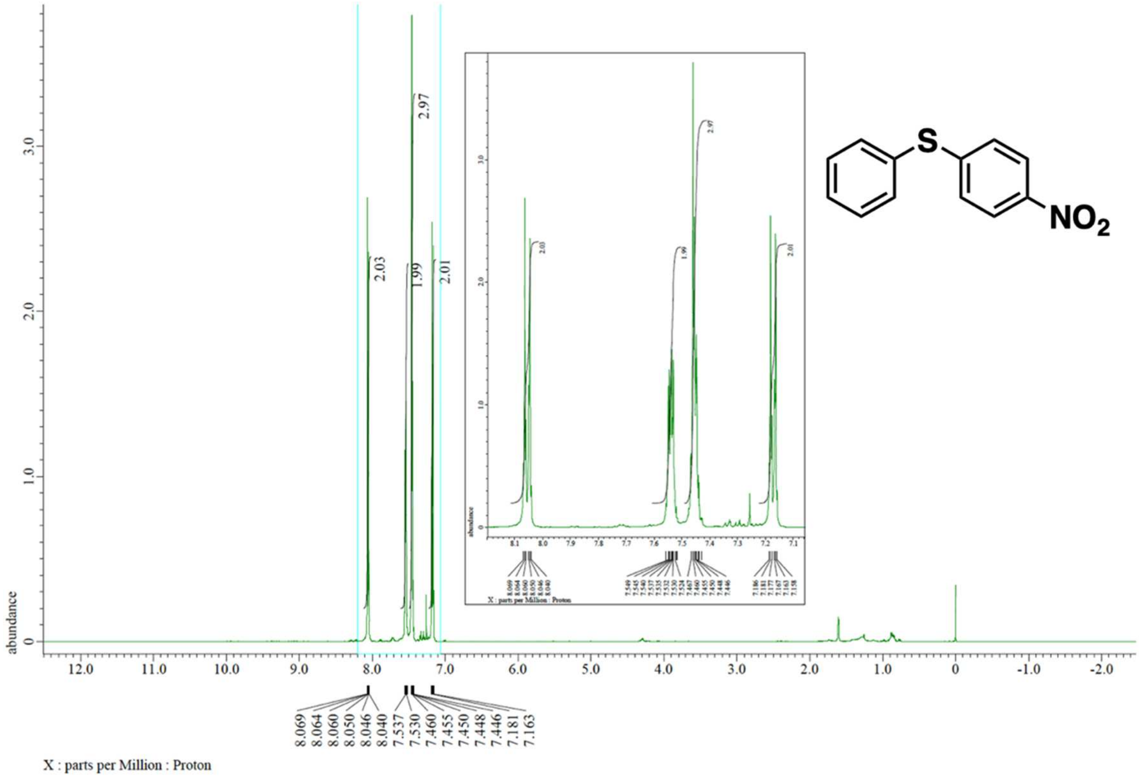

^{13}C NMR spectrum (125 MHz, $CDCl_3$) of **1aq**

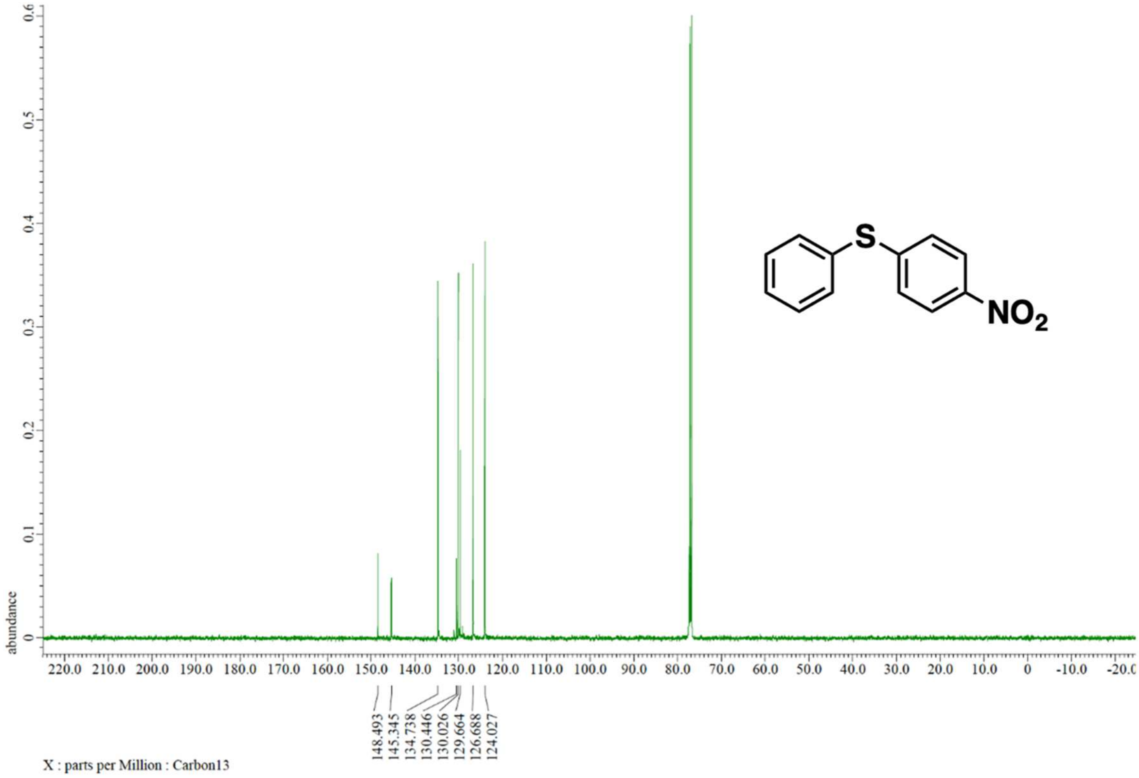

^{1}H NMR spectrum (500 MHz, $CDCl_3$) of **1br**

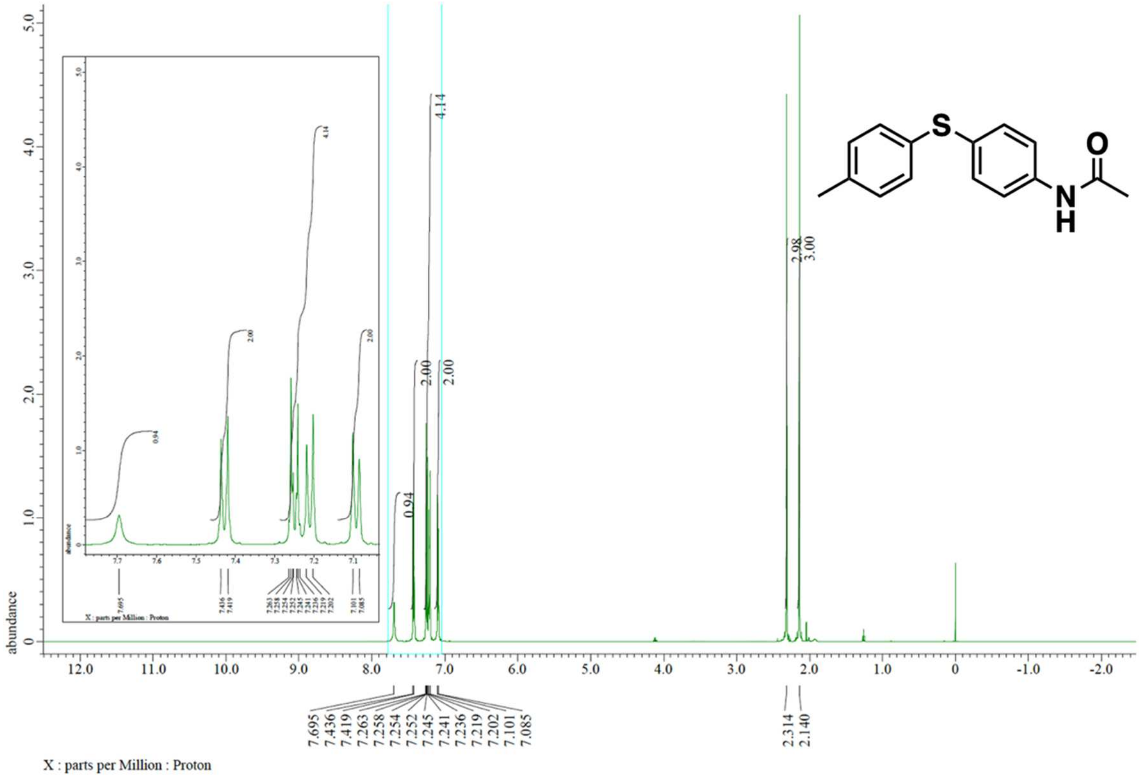

^{13}C NMR spectrum (125 MHz, $CDCl_3$) of **1br**

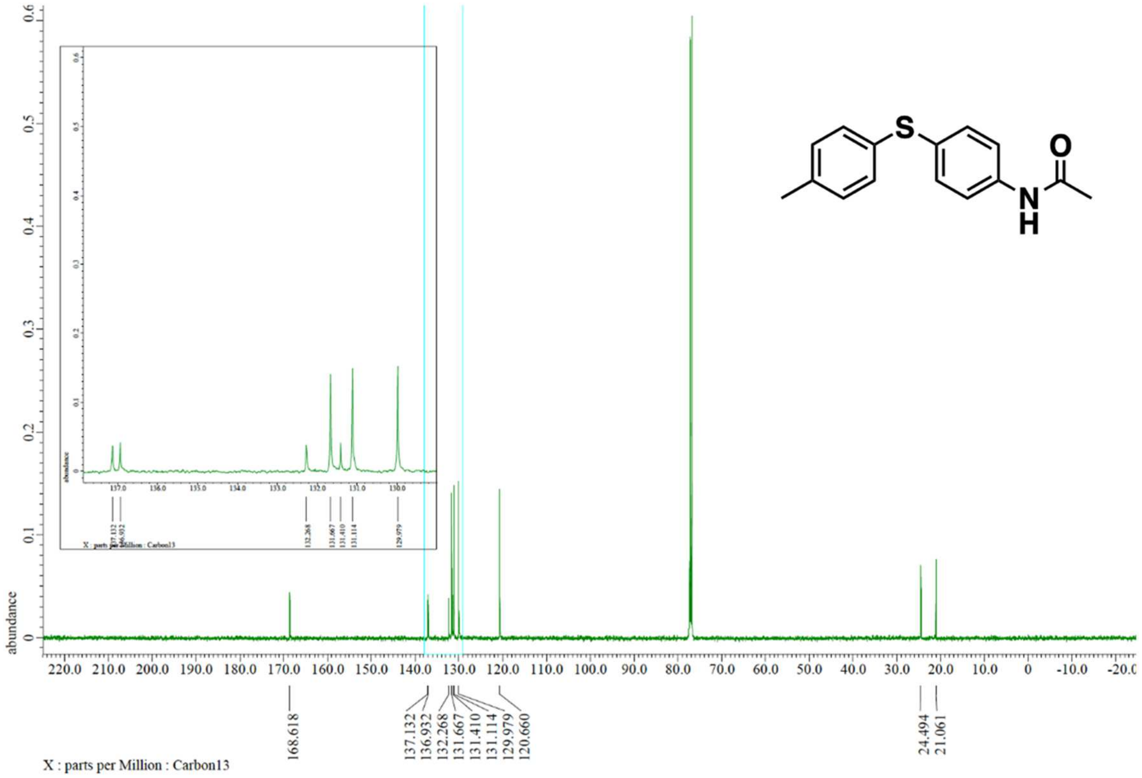

^{1}H NMR spectrum (500 MHz, $CDCl_3$) of **1bs**

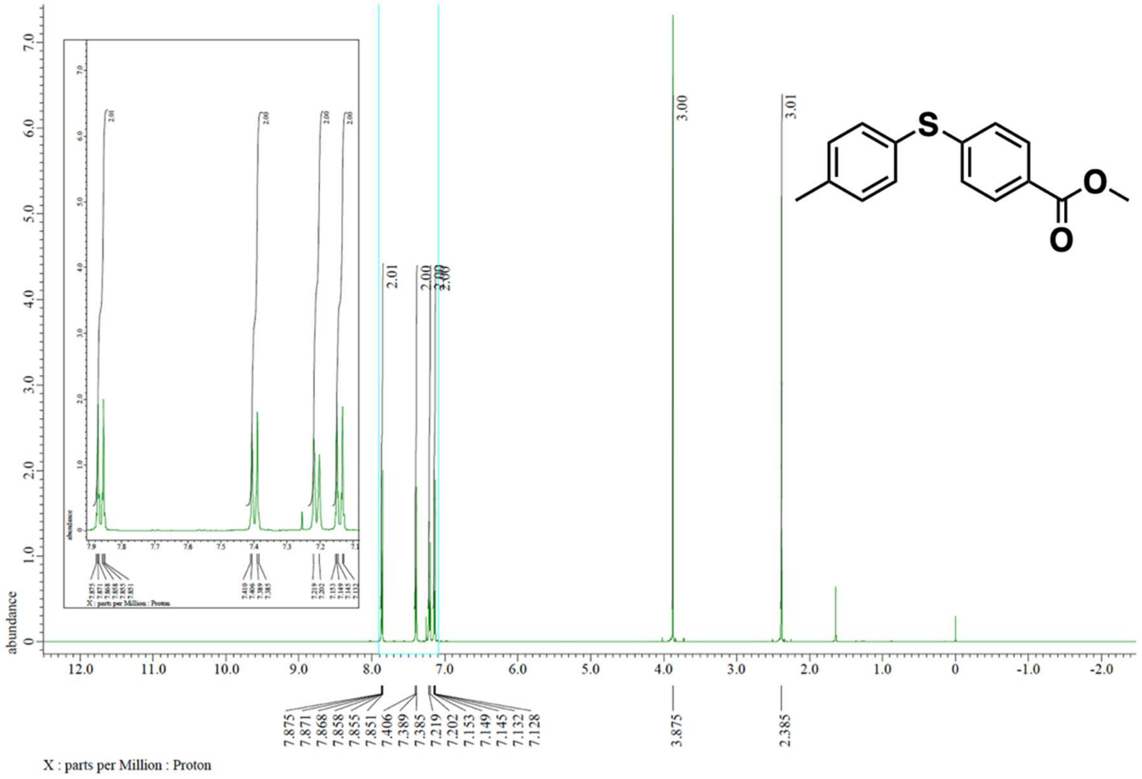

^{13}C NMR spectrum (125 MHz, $CDCl_3$) of **1bs**

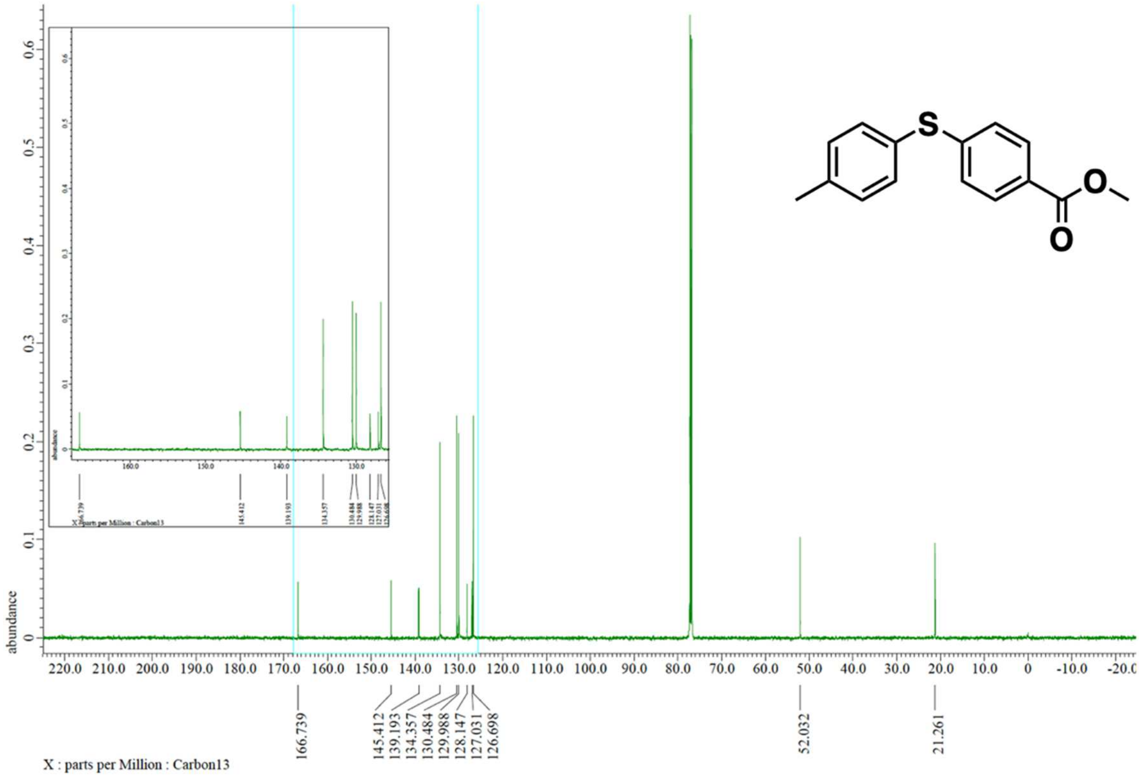

^{1}H NMR spectrum (500 MHz, $CDCl_3$) of **1bt**

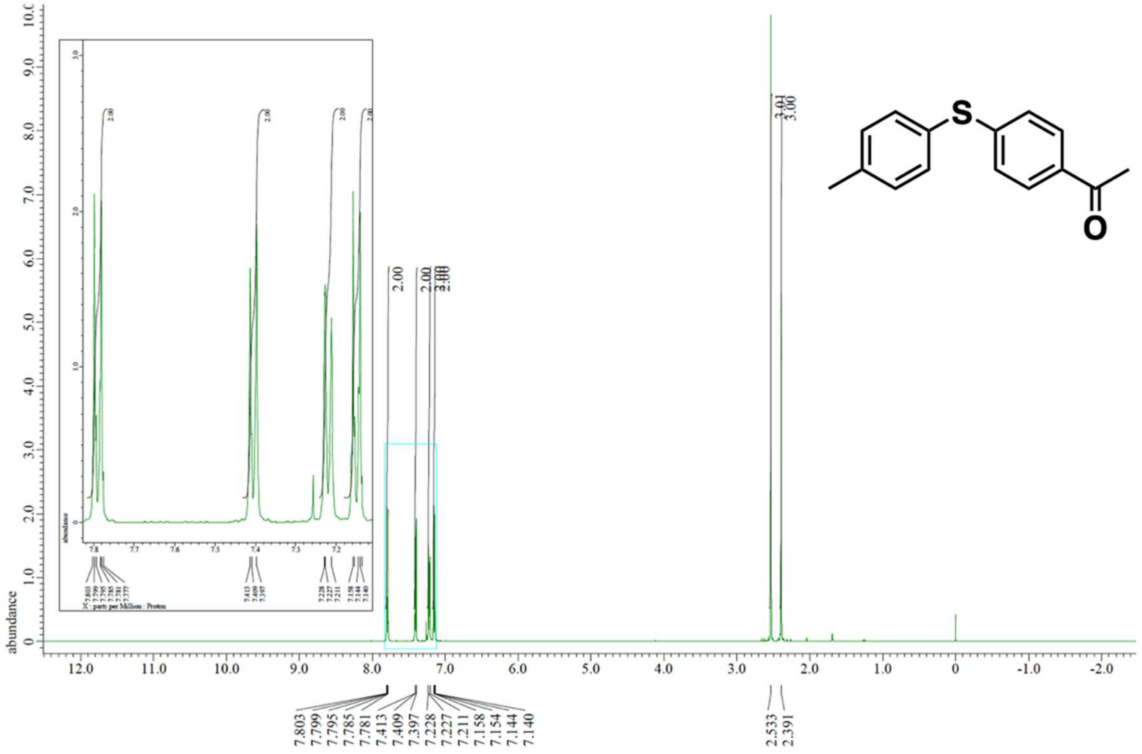

^{13}C NMR spectrum (125 MHz, $CDCl_3$) of **1bt**

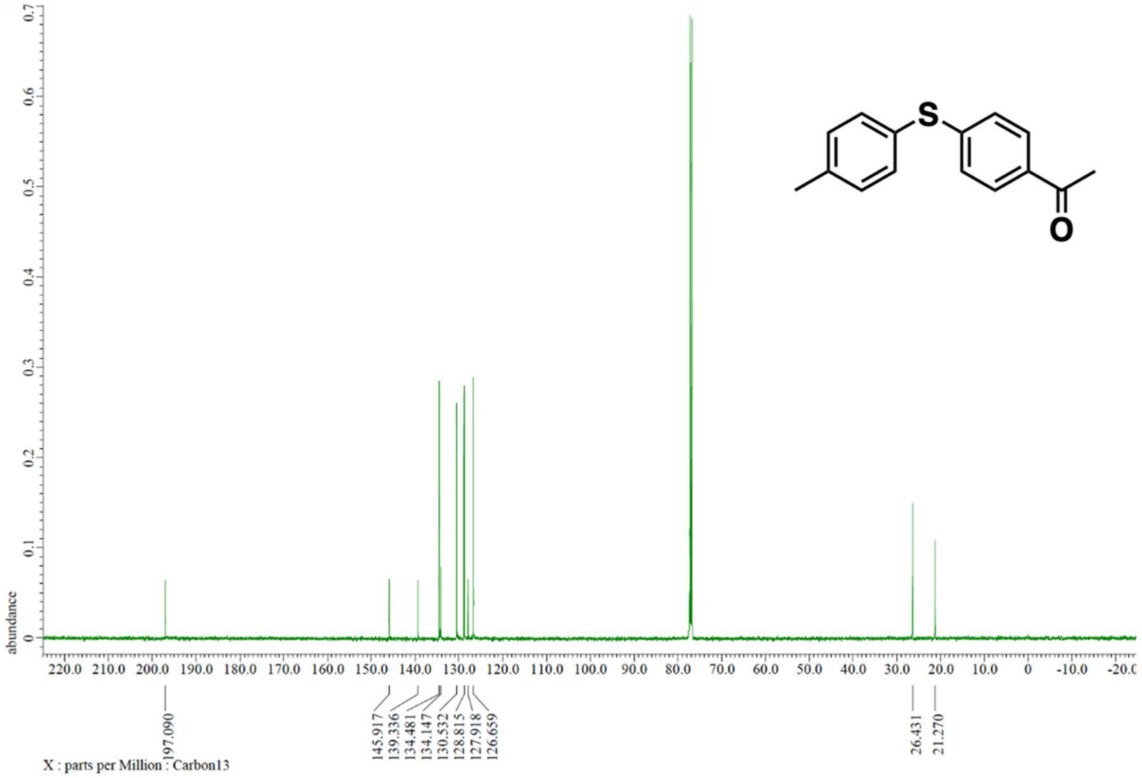

Cartesian Coordinates of Optimized Structures

1a

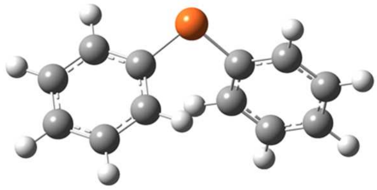

C	3.64652000	-0.02296400	-0.63281400
C	3.66346200	1.08878400	0.20185300
C	2.54102500	1.39414400	0.96867600
C	1.41119200	0.58836500	0.91326600
C	1.39003700	-0.52012500	0.06316300
C	2.50956000	-0.82144200	-0.71280900
S	-0.00019700	-1.63807500	0.00015500
C	-1.39013900	-0.51975500	-0.06304000
C	-2.51081500	-0.82238300	0.71077100
C	-3.64756900	-0.02365800	0.63058600
C	-3.66323000	1.08961000	-0.20209200
C	-2.53966600	1.39623300	-0.96673500
C	-1.40996400	0.59026000	-0.91115900
H	4.51668000	-0.26482200	-1.23890900
H	4.54836200	1.71838400	0.25548900
H	2.55008200	2.26022700	1.62681400
H	0.53942600	0.81684100	1.52302500
H	2.48229400	-1.67894100	-1.38221300
H	-2.48459900	-1.68108400	1.37868200
H	-4.51861600	-0.26652200	1.23500500
H	-4.54799600	1.71938600	-0.25583600
H	-2.54767000	2.26351700	-1.62330700
H	-0.53729500	0.81980300	-1.51922200

Pd_{20}

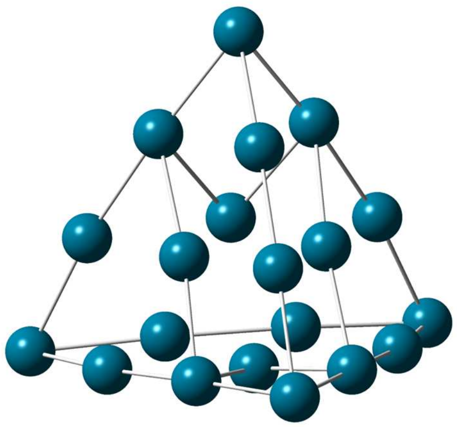

Pd	0.03855700	3.94712900	2.68423600
Pd	-0.03896200	-3.96626400	2.65646200
Pd	3.94617900	-0.02829000	-2.95914500
Pd	-3.94541600	0.04888200	-2.95970700
Pd	-1.41196000	0.00920500	0.96356400
Pd	1.41152200	-0.01529100	0.96340400
Pd	-0.01558000	-1.62592900	-0.93528700
Pd	0.01522500	1.63213600	-0.92465200
Pd	0.01285300	1.29973900	2.97665500
Pd	1.30135800	-0.00277000	-2.71456500
Pd	-1.30054700	0.02060300	-2.71531700
Pd	-0.01304500	-1.32112200	2.96769000
Pd	-1.52221800	2.68416200	0.96282900
Pd	2.87102600	-1.44540400	-0.95697500
Pd	1.52207000	-2.69046200	0.94476500
Pd	-2.87046800	1.45256700	-0.94814000
Pd	1.57448200	2.65769400	0.95996600
Pd	2.89939300	1.40023400	-0.94909700
Pd	-1.57519800	-2.66391100	0.94208300
Pd	-2.89927100	-1.39290600	-0.95877000

$Au_{16}Pd_4$

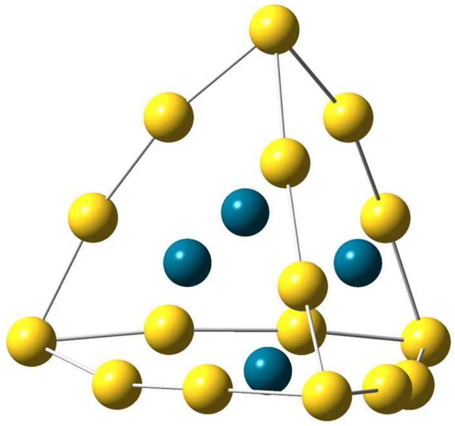

Au	-0.17181100	-4.16555700	-2.88299200
Au	-4.16623900	0.17112300	2.88310100
Au	0.17123300	4.16675600	-2.88114400
Au	4.16609600	-0.17277500	2.88297500
Pd	-0.07411800	-1.80217400	0.64363600
Pd	-1.80294500	0.07350700	-0.64437500
Pd	0.07428200	1.80236700	0.64347900
Pd	1.80307300	-0.07422700	-0.64396500
Au	-2.02498400	-3.01007800	-1.07192000
Au	2.02623700	3.01126600	-1.07080900
Au	3.17099600	1.77005200	1.07029400
Au	-3.17013600	-1.77100200	1.07053200
Au	1.77028400	-3.16919300	-1.07039900
Au	-1.77119800	3.16968100	-1.06947100
Au	-3.01252400	2.02414800	1.07097000
Au	3.01097200	-2.02559700	1.07145100
Au	-0.05594300	-1.39936700	-2.35206200
Au	0.05707600	1.40052300	-2.35076300
Au	-1.40034700	0.05659000	2.34995700
Au	1.40012000	-0.05626100	2.35099200

$Au_{18}Pd_2$

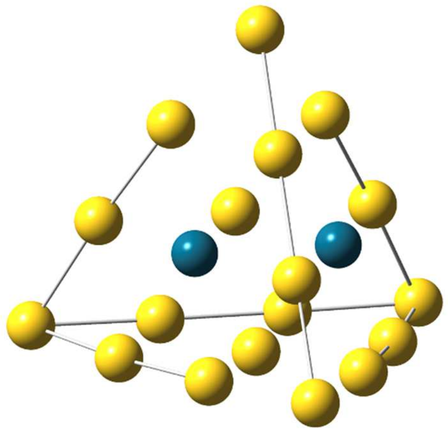

Au	-4.19441400	0.02824400	-2.94858800
Au	-0.01919600	-4.16763400	3.03047600
Au	0.02023700	4.15111100	3.05278600
Au	4.19393600	-0.01085700	-2.94961100
Au	-0.00810100	-1.55906600	-0.95245400
Pd	-1.55448700	0.00348200	1.02773700
Pd	1.55530000	-0.00985400	1.02654700
Au	0.00699000	1.56342600	-0.94335000
Au	-2.97264600	-1.51402300	-0.94515200
Au	1.68625500	2.85333300	1.09697800
Au	2.97235400	1.51934200	-0.93736400
Au	-1.68642900	-2.85996200	1.08192400
Au	-1.37823400	0.01564600	-3.03343100
Au	0.00704400	1.39658700	2.99784700
Au	-0.00541500	-1.41345100	2.99010800
Au	1.37715600	0.00219400	-3.03327000
Au	-2.95804100	1.54604600	-0.93587100
Au	-1.65919000	2.86856600	1.09794000
Au	1.65936500	-2.87438200	1.08115000
Au	2.95785500	-1.54141200	-0.94628300

Pd20_**1a**_C

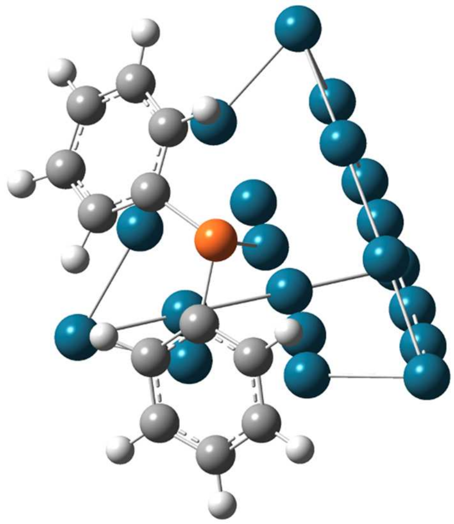

Pd	2.98667200	-0.00105400	3.68398600
Pd	-4.78833300	0.00646200	2.05953800
Pd	0.07004800	-3.98617200	-2.75702300
Pd	0.07814300	3.98545900	-2.75896200
Pd	-0.52413200	1.51806500	0.96423800
Pd	-0.52836000	-1.51896100	0.96116100
Pd	-1.66833300	0.00251200	-1.23068800
Pd	1.14805400	-0.00190100	-0.66400800
Pd	0.40984900	-0.00261900	3.08645400
Pd	0.08615700	-1.32659500	-2.85156200
Pd	0.09223000	1.32504900	-2.85164700
Pd	-2.17934500	0.00053500	2.61707100
Pd	2.21212100	1.51646000	1.58192500
Pd	-1.67532100	-2.80018600	-1.13882300
Pd	-3.27067900	-1.45844100	0.45420500
Pd	1.25310200	2.89956600	-0.61207200
Pd	2.20916500	-1.52040900	1.58412400
Pd	1.24897600	-2.90029400	-0.61212900
Pd	-3.26569200	1.46598000	0.45567600
Pd	-1.66684200	2.80357300	-1.13752800
H	3.22971300	-2.54722100	-2.61498300
S	3.34431700	-0.00319100	-1.63650000
C	3.41872700	-2.63432700	-1.54560300
H	3.23318600	2.54024800	-2.61544300

C	3.85268100	1.50545900	-0.81419200
C	3.42300400	2.62765100	-1.54624800
H	3.03972100	-4.77655300	-1.50558700
C	3.84952300	-1.51244000	-0.81370800
C	3.25075900	-3.89272700	-0.90709800
C	4.18942500	1.64384700	0.55925200
C	3.25673500	3.88643700	-0.90813500
H	4.75504900	0.88285400	1.08235500
H	3.04722500	4.77055600	-1.50673800
C	4.00720500	2.90293200	1.19488300
C	3.49676800	3.99833500	0.47345700
C	4.18553900	-1.65088300	0.56001000
H	4.38209900	3.04513600	2.20669500
C	3.49005000	-4.00456200	0.47449000
H	3.34364400	4.95055200	0.97339500
H	4.75223800	-0.89040700	1.08266500
C	4.00157700	-2.90950700	1.19592600
H	3.33555900	-4.95636800	0.97478400
H	4.37643500	-3.05227900	2.20767900

Pd20_**1a**_S

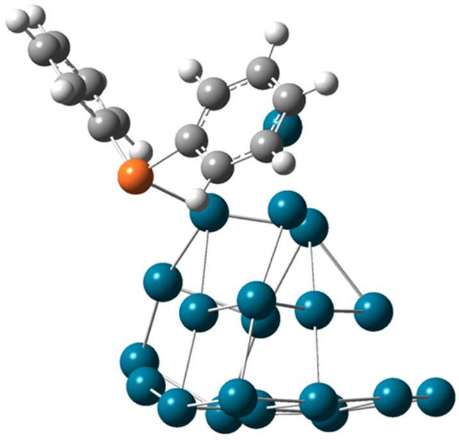

Pd	4.33878700	-1.35580400	2.99082200
Pd	0.22798300	4.55027900	0.81676100
Pd	-3.60060800	-1.94214400	2.05096300
Pd	1.18387300	-1.29869000	-4.13352900
Pd	2.16020100	0.72301400	-0.99516400
Pd	0.51174400	1.02232300	1.37700900
Pd	-0.70640100	0.56609200	-1.35805900
Pd	0.61754400	-1.56208300	0.04693800
Pd	3.35128700	0.52333400	1.31344600
Pd	-2.01114100	-2.48269900	-0.14511900
Pd	-0.58097300	-1.89995100	-2.30848500
Pd	2.25962500	2.89796800	0.67766900
Pd	3.50275300	-2.01545700	0.54268800
Pd	-2.60870700	0.18648300	0.52057400
Pd	-1.43506900	2.54648800	0.59115600
Pd	2.46647300	-1.71585500	-1.84722800
Pd	1.65786300	-1.30906100	2.50032000
Pd	-0.99302900	-1.06086100	2.16906000
Pd	0.45916900	3.03080300	-1.45980900
Pd	0.95082800	1.13477900	-3.28959100
H	-3.40649900	-4.26717300	-1.72548200
C	-3.84934900	-3.54444600	-1.04310400
H	-3.51645000	-1.84634900	-2.37806900
C	-3.91085500	-2.17413600	-1.41604000
C	-4.50835200	-3.98114500	0.12112500

H	-4.47046000	-5.03046900	0.40035900
C	-4.64490600	-1.26121900	-0.61861300
S	-4.44125700	0.48157900	-1.03765500
C	-5.25289000	-3.08019900	0.89661700
C	-5.77680100	1.25347800	-0.12266600
C	-5.32163800	-1.70300500	0.53218800
C	-7.05997700	1.23641700	-0.67071500
C	-5.52671300	1.88279500	1.09643200
H	-7.23471400	0.74736100	-1.62723600
H	-4.51427100	1.87862300	1.50573100
H	-5.88695400	-3.45302600	1.69888800
C	-8.10294800	1.84612100	0.01764600
C	-6.57641700	2.49936500	1.77209000
C	-7.86042800	2.47840800	1.23533300
H	-9.10589400	1.83381200	-0.40152600
H	-6.38694900	2.99297100	2.72192000
H	-5.99386900	-1.02738600	1.05979200
H	-8.67746400	2.95980300	1.76704300

Pd20_**1a**_T

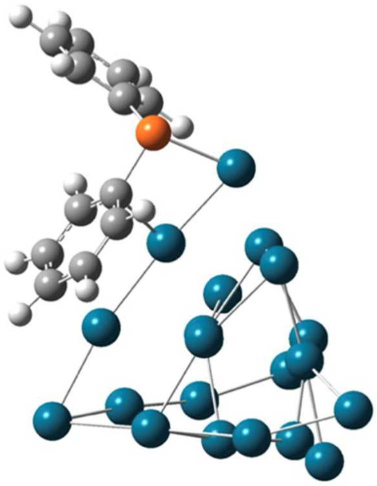

Pd	2.06254500	-3.69839200	2.00860500
Pd	-0.20129200	3.79058100	2.71889500
Pd	-3.55834400	-1.30667100	-2.25159400
Pd	2.98155500	0.67672600	-3.73956900
Pd	2.32810800	0.83272800	0.76469300
Pd	-0.12944900	-0.30358600	0.63571300
Pd	-0.30275500	1.55647600	-1.19534800
Pd	1.33564300	-2.51936600	-0.33414900
Pd	1.50703500	-1.19109700	2.80621200
Pd	-0.98597400	-0.46814700	-2.76213700
Pd	1.50224300	-0.48570800	-1.89112400
Pd	0.55539400	1.25917400	2.77928000
Pd	3.44629000	-1.57245900	0.90721600
Pd	-2.71254400	0.15926200	-0.24639900
Pd	-1.66013600	2.06729100	1.31518900
Pd	3.99796600	-0.01278700	-1.24469700
Pd	-0.53433200	-2.70930700	1.77316200
Pd	-1.29388800	-2.23771000	-0.81219200
Pd	1.05034200	3.09617400	0.46886200
Pd	2.38731100	2.26060400	-1.68771900
H	-6.28132800	-3.58662800	1.50510900
C	-6.65440200	-2.66841200	1.05875300
H	-4.89625200	-2.37065100	-0.17907500
C	-5.88044400	-1.99978100	0.11472400

C	-7.89730400	-2.16221200	1.42954300
H	-8.49813600	-2.68917600	2.16663900
C	-6.36644200	-0.82068300	-0.45028600
S	-5.40893400	0.07787400	-1.67455000
C	-8.37645300	-0.98578400	0.85778700
C	-4.51487500	1.26828600	-0.64841200
C	-7.61327500	-0.31037900	-0.08849100
C	-3.99285900	2.42143400	-1.33127600
C	-4.49355500	1.22130300	0.77447000
H	-4.02625400	2.44686200	-2.41918100
H	-5.01683400	0.43244400	1.31005800
H	-9.34893400	-0.59456900	1.14565500
C	-3.41960100	3.44228700	-0.62070000
C	-3.92838400	2.30221900	1.49883300
C	-3.35323800	3.40051600	0.80963300
H	-2.99791600	4.29670300	-1.14411200
H	-4.05469600	2.33556000	2.57887900
H	-7.97722000	0.60789300	-0.54515300
H	-3.09759100	4.30863300	1.35317000

$Au_{16}Pd_4$_**1a**_C

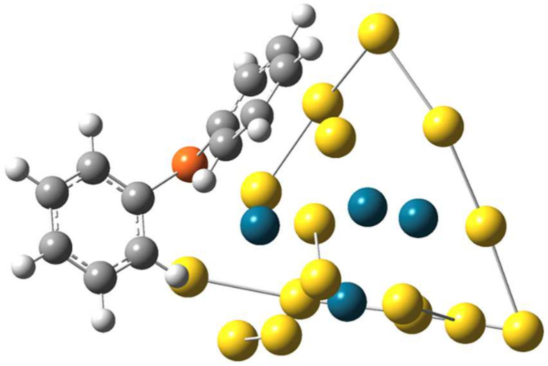

Au	-2.38871400	-3.12529800	2.98389900
Au	4.72151100	-2.19192500	-1.37179800
Au	1.28840900	4.28233600	2.46019000
Au	-2.75535300	1.10435200	-3.38827000
Pd	-0.08862500	-1.56216100	-0.61957800
Pd	1.37844000	-0.57368700	1.54448400
Pd	1.24239100	1.24184100	-0.74023600
Pd	-1.81247600	0.77485600	-0.61580400
Au	0.19170600	-3.19210700	1.77533600
Au	0.58178900	3.98772600	-0.19658500
Au	-0.69596200	2.79965100	-2.30325600
Au	2.44336700	-2.89853600	0.26555300
Au	-2.46341000	-2.92158900	0.15038300
Au	2.66745800	1.96922500	1.69869300
Au	3.78308900	-0.04405300	0.16827100
Au	-2.48333700	-1.54010300	-2.23316900
Au	-1.51996700	-0.72968000	1.76091300
Au	-0.23109300	1.84821800	1.60768300
Au	2.25821500	-1.08384400	-2.07121300
Au	-0.15509600	0.04899900	-2.96858200
H	-2.22640200	3.86771700	1.97803900
S	-3.64979000	2.07956200	0.29522100
C	-2.52814200	3.02475500	2.60082100
H	-4.41804100	-0.60481900	-0.58033900
C	-5.23153700	1.23585500	0.17932500
C	-5.32323900	-0.07162300	-0.28684900
H	-1.48127800	3.73009500	4.34140400

C	-3.35763100	2.02430500	2.07479000
C	-2.13098800	2.95514700	3.93930700
C	-6.37449400	1.93921400	0.56427800
C	-6.57024400	-0.68593700	-0.36394500
H	-6.28813400	2.96482300	0.91874700
H	-6.63816500	-1.70928200	-0.72672300
C	-7.61468800	1.31525700	0.49129300
C	-7.71319800	0.00454100	0.02691500
C	-3.82439000	0.99344200	2.88952800
H	-8.50814300	1.85726900	0.79114600
C	-2.57842900	1.91470400	4.75195500
H	-8.68611800	-0.47716800	-0.03223500
H	-4.47317100	0.21930500	2.48186000
C	-3.43674200	0.95138200	4.22982600
H	-2.26399800	1.86104800	5.79056100
H	-3.79147500	0.13630900	4.85677500

$Au_{18}Pd_2$_**1a**_C

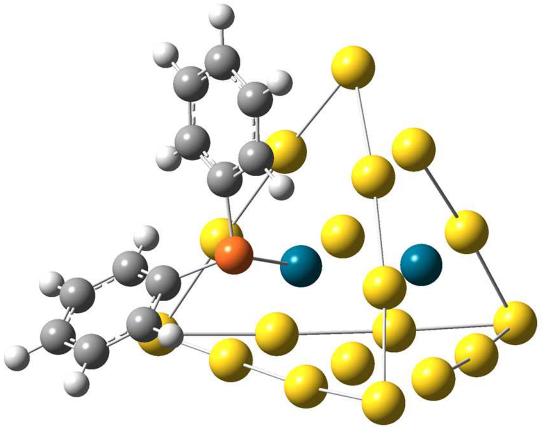

Au	-1.48819000	3.82169800	2.85584800
Au	5.37392800	-0.36888700	0.44013600
Au	-0.98578400	0.85242500	-4.94837800
Au	-1.94235800	-4.42331600	1.47970000
Au	0.77349900	-0.36535900	1.83642000
Au	1.11066300	1.65374600	-0.58591000
Pd	0.92856400	-1.33797200	-0.99991700
Pd	-1.52063900	-0.01960000	-0.30529500
Au	0.94753000	2.55610300	2.18070600
Au	-1.20534800	-1.13608400	-2.96544300
Au	-1.54380700	-2.86287500	-0.82693000
Au	3.21615500	1.18341600	1.39836500
Au	-1.62671500	1.02022700	2.57112000
Au	1.26505200	0.42565600	-3.22840500
Au	3.38109400	0.07964400	-1.46922600
Au	-1.81623400	-1.69773100	2.10518900
Au	-1.37607300	2.90909100	0.20339300
Au	-1.15848800	1.99170300	-2.40657400
Au	3.04424000	-1.87672400	0.86577800
Au	0.61970000	-3.19732400	1.14583500
H	-5.22772400	2.09214700	-2.23156400
S	-3.81127300	0.14389300	-0.84376100
C	-5.24369500	2.40821700	-1.19027800
H	-4.66632700	-2.21395200	-2.05091000
C	-4.66998700	-1.26464400	-0.11568000
C	-4.92881400	-2.31371600	-0.99858700

H	-6.30276700	4.23563400	-1.58048700
C	-4.64177300	1.61009800	-0.21586700
C	-5.84155600	3.60920800	-0.82125700
C	-4.98918900	-1.37872900	1.23603300
C	-5.50852300	-3.48606200	-0.52197000
H	-4.77093700	-0.57383400	1.93239500
H	-5.70279800	-4.30602000	-1.20879100
C	-5.58716700	-2.54806900	1.69834500
C	-5.84064500	-3.60308600	0.82524000
C	-4.63188000	2.01013400	1.11930900
H	-5.83525700	-2.63770800	2.75319800
C	-5.84626000	4.00560100	0.51307200
H	-6.29435600	-4.51859100	1.19652900
H	-4.11006700	1.41839300	1.87153900
C	-5.24597700	3.20432500	1.48124600
H	-6.31096800	4.94589800	0.79903700
H	-5.23045300	3.51708500	2.52310800

An initial structure of the **1a** oxidative adduct only on the Pd atom of $Au_{18}Pd_2$

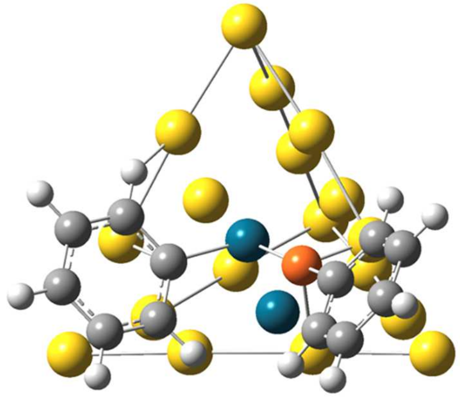

Au	-4.19441000	0.02824000	-2.94859000
Au	-0.01920000	-4.16763000	3.03048000
Au	0.02024000	4.15111000	3.05279000
Au	4.19394000	-0.01086000	-2.94961000
Au	-0.00810000	-1.55907000	-0.95245000
Pd	-1.55449000	0.00348000	1.02774000
Pd	1.55530000	-0.00985000	1.02655000
Au	0.00699000	1.56343000	-0.94335000
Au	-2.97265000	-1.51402000	-0.94515000
Au	1.68626000	2.85333000	1.09698000
Au	2.97235000	1.51934000	-0.93736000
Au	-1.68643000	-2.85996000	1.08192000
Au	-1.37823000	0.01565000	-3.03343000
Au	0.00704000	1.39659000	2.99785000
Au	-0.00541000	-1.41345000	2.99011000
Au	1.37716000	0.00219000	-3.03327000
Au	-2.95804000	1.54605000	-0.93587000
Au	-1.65919000	2.86857000	1.09794000
Au	1.65936000	-2.87438000	1.08115000
Au	2.95785000	-1.54141000	-0.94628000
H	4.70239000	3.94681000	1.68548000
H	5.34348000	-5.40604000	2.68746000
H	3.44841000	2.24783000	0.37976000
H	3.88807000	-3.87608000	1.38175000
C	4.00923000	3.28005000	2.19325000
C	3.30697000	2.32845000	1.45595000

C	4.80722000	-4.60763000	3.19524000
C	3.99202000	-3.75079000	2.45793000
C	3.82120000	3.39187000	3.56818000
H	4.35781000	4.14668000	4.13717000
C	4.95000000	-4.44187000	4.57016000
C	2.40066000	1.46657000	2.08442000
H	5.60015000	-5.10140000	5.13915000
C	3.30062000	-2.70859000	3.08640000
S	2.31279000	-1.58030000	2.13133000
C	2.94312000	2.52270000	4.21694000
C	2.23778000	1.55893000	3.48627000
C	4.24651000	-3.42621000	5.21892000
C	3.41986000	-2.56422000	4.48825000
H	2.81125000	2.57916000	5.29606000
H	4.32501000	-3.30614000	6.29805000
H	1.61584000	0.82434000	3.99697000
H	2.80443000	-1.82418000	4.99895000

An optimized structure of the **1a** oxidative adduct only on the Pd atom of $Au_{18}Pd_2$

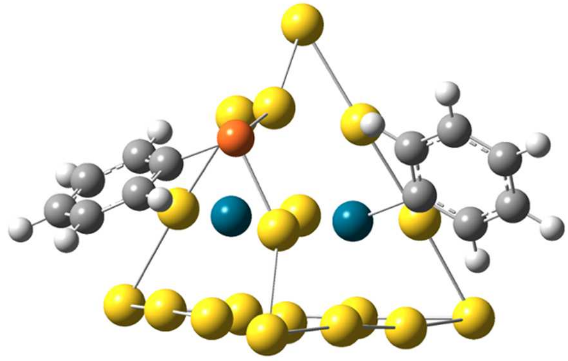

Au	-3.09601700	-4.19875500	0.39062900
Au	-1.16299100	3.23826300	3.81523900
Au	-0.20548300	1.67205700	-4.91249200
Au	4.90209800	-1.65188500	0.78506100
Au	0.16193700	-0.91907700	1.73807300
Pd	-1.53571800	0.41030600	-0.29759500
Pd	1.22537300	1.42229500	-0.09817300
Au	0.53486800	-1.46483800	-1.28278800
Au	-2.64044800	-1.63337400	1.46359300
Au	1.49145300	0.86350000	-2.84353100
Au	3.24339600	-0.36895400	-1.07419500
Au	-2.01964500	0.81802500	2.55958800
Au	-0.36512200	-3.56065500	0.55717800
Au	-0.99928500	2.43841000	-2.37105900
Au	-0.94146800	3.10966700	1.03689900
Au	2.26938700	-2.69715100	0.67983800
Au	-2.28642200	-2.19847300	-1.41739600
Au	-1.31371100	-0.33813200	-3.18924600
Au	0.95646600	1.73331300	2.75840400
Au	2.90537300	-0.02145400	1.90650200
H	5.51995900	4.16371200	0.65364300
H	-6.14579800	3.23151500	1.95235900
H	4.14770800	2.13370800	0.85596300
H	-4.14181300	4.43433900	1.13469200
C	4.54710600	4.13171100	0.16736600
C	3.77001100	2.97886000	0.28366600
C	-5.57676500	2.82352000	1.12034900

C	-4.45687900	3.50824300	0.65666200
C	4.08704800	5.22796200	-0.55619400
H	4.69877400	6.12276800	-0.64144500
C	-5.95322100	1.61497200	0.53995700
C	2.51614800	2.93480400	-0.32615200
H	-6.81523300	1.07179000	0.91861500
C	-3.71132200	2.98543900	-0.40192600
S	-2.22341100	3.89907000	-0.88271800
C	2.84255200	5.17027600	-1.17405100
C	2.05508700	4.02185300	-1.07012100
C	-5.21956600	1.10587000	-0.52810600
C	-4.10705100	1.79234600	-1.01000100
H	2.47238300	6.01586400	-1.74996200
H	-5.49951800	0.16196400	-0.99174500
H	1.08986000	3.99160800	-1.57596700
H	-3.55122200	1.39429800	-1.85959800

Ph spill-over (near another Pd)

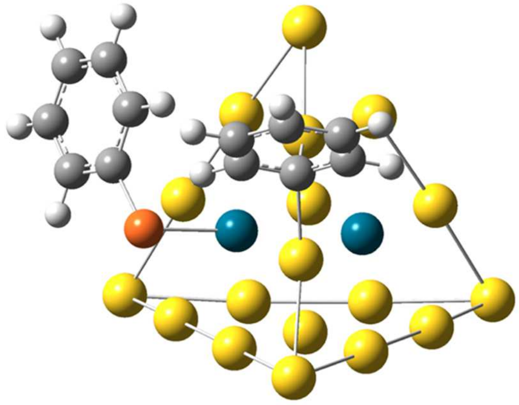

Au	-4.74558400	-1.42724800	-2.04460200
Au	3.28364900	0.35885300	-3.80547400
Au	1.14671500	-3.56753600	3.55084400
Au	-0.70844100	4.53248300	2.30664300
Au	-0.80021200	1.27355100	-1.32289300
Pd	-0.04993900	-1.54547500	-0.76331800
Pd	1.41896400	0.42422800	0.72578000
Au	-1.60057200	-0.16589600	1.37924700
Au	-2.05924100	-0.92988800	-2.75050000
Au	0.62696500	-0.84930500	3.15313000
Au	0.06660700	1.88082500	2.73936300
Au	0.58267400	-0.38147400	-3.32242700
Au	-3.58314500	0.67697400	-0.58029100
Au	2.01853300	-2.50226100	1.09641200
Au	2.76470100	-1.10207300	-1.46875500
Au	-2.22516300	2.60363600	0.89613700
Au	-2.80969100	-2.28193700	-0.20070900
Au	-0.85242300	-2.97815700	1.62060200
Au	2.00899600	1.82172400	-1.77560400
Au	0.63119500	3.29465100	0.13531100
H	5.12362700	-5.45692800	-1.48902800
H	5.58223600	3.11285900	-1.74619700
H	2.95965600	-4.24864000	-1.40838000
H	4.81528600	1.24266900	-0.31603100
C	5.04953000	-4.51157700	-0.95653600
C	3.83589600	-3.83499200	-0.91269800

C	5.00394700	3.32347600	-0.84860200
C	4.57443700	2.27020400	-0.04681600
C	6.16812800	-3.97904200	-0.31622300
H	7.11499000	-4.51308200	-0.35230200
C	4.67570200	4.63686100	-0.51463300
C	3.72960000	-2.60416400	-0.22436600
H	4.99680700	5.45725600	-1.15162700
C	3.80224700	2.52207700	1.09674800
S	3.16413800	1.18289700	2.07526400
C	6.08217700	-2.76989200	0.37169800
C	4.87425000	-2.08143700	0.42004400
C	3.94451100	4.89700600	0.64389900
C	3.51389400	3.84792700	1.45011400
H	6.95719300	-2.36301200	0.87328400
H	3.69715800	5.92068100	0.91593900
H	4.79636800	-1.13849000	0.96455800
H	2.92984000	4.04227300	2.34828000

Ph spill-over (far from another Pd)

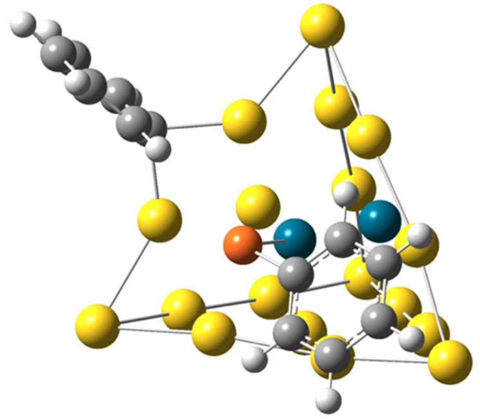

Au	-5.18560400	-1.35066700	-0.24962700
Au	1.01107800	2.72634400	-4.20315700
Au	2.70387900	-4.39849500	-0.06427300
Au	0.69356800	2.47583100	4.33883600
Au	-1.29511300	1.40386100	-0.03082000
Pd	-0.49040600	-1.00843800	-1.52224700
Pd	1.58296000	0.15584300	-0.01951500
Au	-0.68608500	-1.24226000	1.53074100
Au	-3.20457000	0.03566500	-1.68393800
Au	2.05629600	-2.20900600	1.52574500
Au	1.51408500	0.26812600	2.80016200
Au	-1.14383900	1.42213700	-2.92985500
Au	-3.37502900	-0.01662600	1.42373100
Au	2.19835600	-2.11854300	-1.66818600
Au	1.68001800	0.30060800	-2.90245400
Au	-1.38127500	1.25235700	2.87945400
Au	-2.60257600	-2.50519500	-0.20664300
Au	0.00109300	-3.48763500	-0.18936600
Au	1.01315400	2.76358400	-1.39430200
Au	0.75938100	2.87563900	1.56512900
H	4.28280200	6.14120000	0.22544500
H	6.26877000	-2.21813200	-1.95027700
H	3.48025100	3.79583700	0.28095900
H	4.79452000	-0.25108400	-1.60423600
C	3.21825300	5.93221800	0.14997800
C	2.76865600	4.61623000	0.18344200

C	5.95610100	-1.94817400	-0.94395200
C	5.12994800	-0.84405900	-0.75498900
C	2.30643100	6.97804900	0.01849300
H	2.66319500	8.00516800	-0.00749800
C	6.37455500	-2.70649100	0.14792100
C	1.38823200	4.33427400	0.08428600
H	7.01520300	-3.57201500	-0.00320900
C	4.70990500	-0.49324300	0.53387100
S	3.64790200	0.91765900	0.78081000
C	0.94010200	6.71778800	-0.08072500
C	0.47813900	5.40712600	-0.04876300
C	5.97287000	-2.35137800	1.43485300
C	5.14296900	-1.25171200	1.62954300
H	0.23363900	7.53813200	-0.18377200
H	6.29980300	-2.93715900	2.29092100
H	-0.58774000	5.20058800	-0.12849100
H	4.82005500	-0.97196600	2.63154400

SPh spill-over (near another Pd)

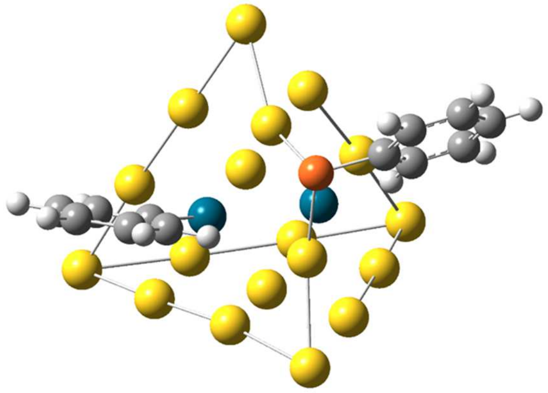

Au	-0.45252600	-5.22871600	-0.26738200
Au	2.21185500	1.60761600	-4.35133800
Au	2.03776400	1.14919200	4.60645000
Au	-4.89486600	1.89510700	0.03002700
Au	-1.06471200	-0.64568500	-1.60699700
Pd	1.39574000	-0.64849400	0.02277500
Pd	-0.02494300	1.89606000	0.05822500
Au	-1.13909900	-0.77639400	1.51901200
Au	0.65060300	-2.99274400	-1.58274500
Au	-0.10906300	1.62848100	2.87654600
Au	-2.50606500	1.81878000	1.49544300
Au	1.53450000	-0.75948900	-2.91477500
Au	-2.11506900	-2.97074900	-0.15756300
Au	2.68246300	1.24859300	1.90785800
Au	2.55712700	1.63140500	-1.58779000
Au	-3.56615400	-0.60310100	-0.05272000
Au	0.55384700	-3.14844600	1.34654500
Au	1.35161100	-1.08832700	2.96695400
Au	-0.11048300	1.86921700	-2.81044000
Au	-2.54922400	1.82885300	-1.51867500
H	2.18167700	6.21749500	1.38491600
H	5.28502900	-2.93227800	-0.28840300
H	1.91771400	3.77814200	1.25707100
H	3.72120500	-1.06780600	0.09975000
C	1.28381100	5.80595300	0.92873700
C	1.13670800	4.41990200	0.84964400

C	5.66425100	-1.91270500	-0.29644100
C	4.78239600	-0.86128300	-0.07850300
C	0.29090800	6.64788300	0.43927500
H	0.40725700	7.72684600	0.50584600
C	7.01598400	-1.66146800	-0.52367900
C	-0.00738500	3.88302100	0.25941100
H	7.70288400	-2.48672100	-0.69379400
C	5.25119200	0.45366900	-0.09249600
S	4.20277200	1.89907700	0.15974600
C	-0.85480200	6.10166800	-0.13123800
C	-1.01511400	4.71852500	-0.22175700
C	7.48225700	-0.35116900	-0.53169000
C	6.60463800	0.70894100	-0.31811700
H	-1.63961300	6.74989200	-0.51567200
H	8.53576700	-0.14602000	-0.70601800
H	-1.91797900	4.31127000	-0.67235400
H	6.96777700	1.73462300	-0.32655800

SPh spill-over (far from another Pd)

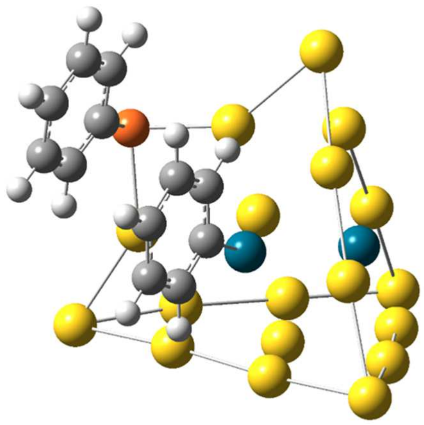

Au	-5.07757800	1.15511300	1.51835500
Au	2.36335700	4.54250000	-0.48129000
Au	-0.43261700	-2.14762300	-4.71849400
Au	1.62541200	-3.52515900	3.27688100
Au	-0.23021500	0.79253600	1.61182900
Pd	-0.99223900	1.14494100	-1.16260000
Pd	1.57828200	-0.39085200	-0.65894300
Au	-1.38471600	-1.56229800	0.00854700
Au	-2.59924800	2.35365200	0.93721900
Au	0.29210300	-2.68882000	-2.07114700
Au	1.05201600	-3.03083700	0.55718100
Au	-0.13291700	3.44433100	0.33591000
Au	-2.85162800	-0.35005600	2.27195600
Au	0.66085800	0.03192200	-3.30160900
Au	1.55366300	2.22915200	-1.87120500
Au	-0.58631700	-1.81846900	2.85942300
Au	-3.64820600	0.09944000	-0.70142800
Au	-2.10429000	-0.95211700	-2.75806800
Au	2.38463200	2.12671300	0.95107400
Au	2.20440600	-0.98078300	2.30015400
H	6.35827100	0.79291300	-1.96900600
H	7.19954500	-1.93196500	0.99965900
H	4.08323500	1.28708200	-1.21475200
H	4.93427300	-1.53134200	1.90917700
C	5.63976600	-0.02116600	-1.89684000
C	4.34441300	0.25940600	-1.46338400

C	6.87096800	-0.91264600	1.18875100
C	5.59639100	-0.69122200	1.69951000
C	6.01057400	-1.32107300	-2.22449100
H	7.02154800	-1.53202600	-2.56592100
C	7.71397500	0.15923800	0.91076800
C	3.41458600	-0.77658800	-1.35066800
H	8.71009600	-0.02021100	0.51342800
C	5.16198200	0.61512000	1.93103400
S	3.58745800	0.96713700	2.71271200
C	5.07978700	-2.34825600	-2.11252800
C	3.77718000	-2.08447400	-1.68424400
C	7.27643400	1.46193600	1.13691200
C	6.00231100	1.69483800	1.64199700
H	5.35321000	-3.37074800	-2.36529600
H	7.92873000	2.30443600	0.91910000
H	3.06088200	-2.89932100	-1.61462900
H	5.65425400	2.71162600	1.81705000
